# Supplementary material for: Impact on mental health care and on mental health service users of the COVID-19 pandemic: a mixed methods survey of UK mental health care staff
Source: Soc Psychiatry Psychiatr Epidemiol. 2020 Aug 28;56(1):25–37. doi: 10.1007/s00127-020-01927-4 (PMC7453694; doi:10.1007/s00127-020-01927-4)
Supplement: Supplementary file 1 — Supplementary file1 (DOCX 900 kb) [file 127_2020_1927_MOESM1_ESM.docx]

**
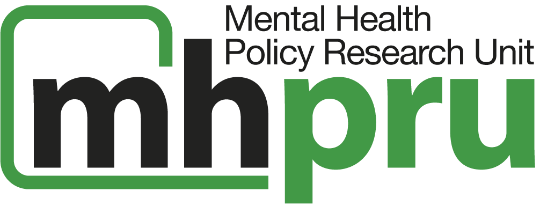

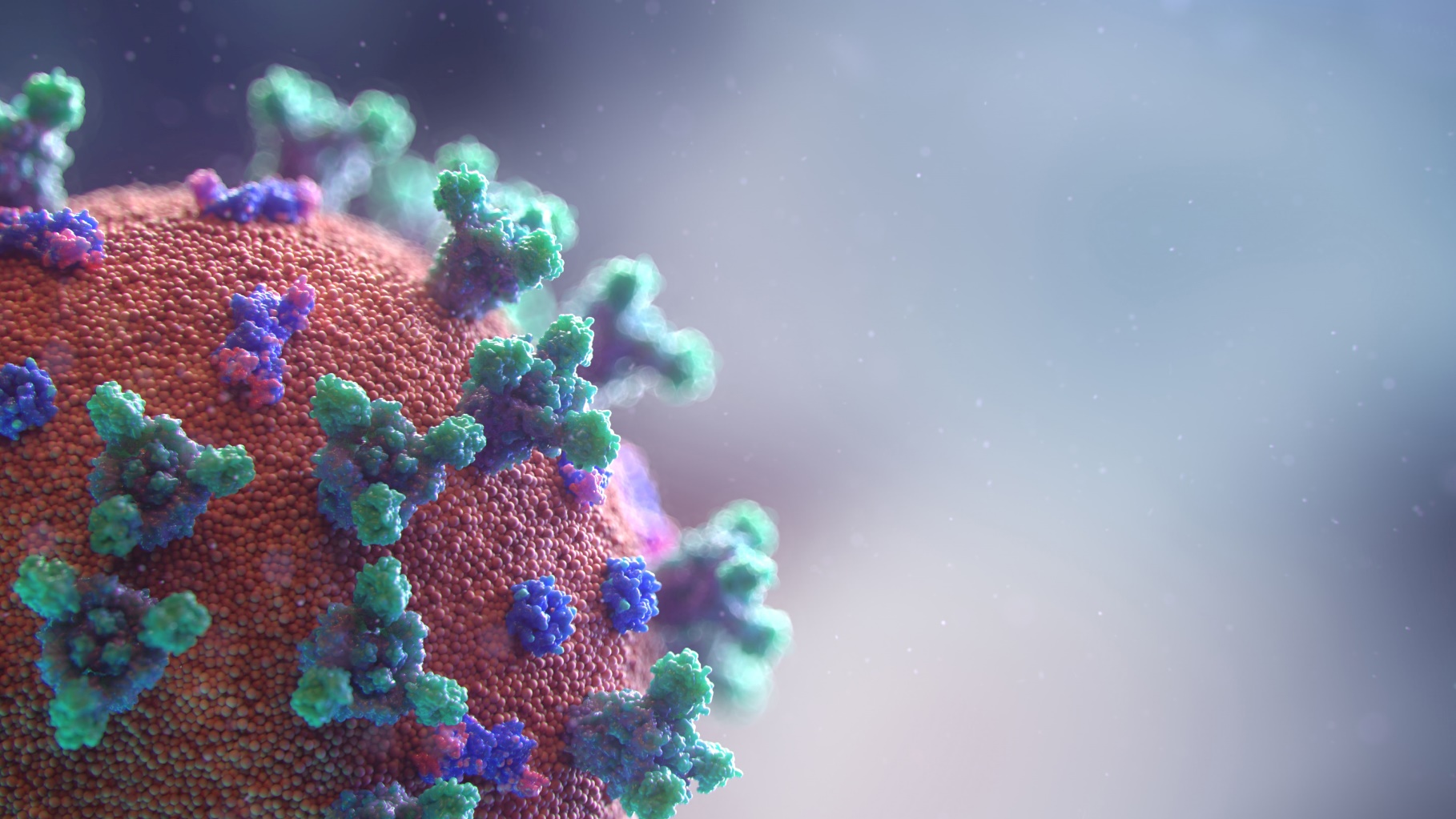
**

**COVID-19: What’s happening in mental health care?**

**Impact on mental health care and on mental health service users of the COVID-19 pandemic: a mixed methods survey of UK mental health care staff**

Supplementary report

12 June 2020

Authors and their institutions:

Sonia Johnson^1,2^, Christian Dalton-Locke*^1^, Norha Vera San Juan^3^, Una Foye^3^, Sian Oram^3^, Alexandra Papamichail^3^, Sabine Landau^3^, Rachel Rowan Olive^4^, Tamar Jeynes^4^, Prisha Shah^4^, Luke Sheridan Rains^1^, Brynmor Lloyd-Evans^1^, Sarah Carr^5^, Helen Killaspy^1,2^, Steve Gillard^6^, Alan Simpson^2,7^, and The COVID-19 Mental Health Policy Research Unit Group**

SJ and CDL are joint first authors

*Corresponding author ([c.dalton-locke@ucl.ac.uk](mailto:c.dalton-locke@ucl.ac.uk))

**List of group members: Andy Bell^8^, Francesca Bentivegna^1^, Joseph Botham^3^, Julian Edbrooke-Childs^9^, Lucy Goldsmith^6^, Lisa Grünwald^1,10^, Jasmine Harju-Seppänen^1,11^, Stephani Hatch^3^, Claire Henderson^3^, Louise Howard^3^, Rebecca Lane^9^, Sarah Ledden^1^, Monica Leverton^1^, Jo Lomani^6^, Natasha Lyons^1^, Paul McCrone^12^, Chukwuma U Ntephe^1,13^, Josephine Enyonam Ocloo^3^, David Osborn^1,2^, Steve Pilling^11^, Konstantina Poursanidou^4^, Hannah Rachel Scott^1^, Thomas Steare^1^, Ruth Stuart^3^, André Tomlin^14^, Kati Turner^6^, and Vasiliki Tzouvara^7^

1. NIHR Mental Health Policy Research Unit, Division of Psychiatry, University College London, London, UK
2. Camden and Islington NHS Foundation Trust, London, UK
3. NIHR Mental Health Policy Research Unit, Institute of Psychiatry, Psychology & Neuroscience, King’s College London, London, UK
4. Division of Psychiatry (NIHR Mental Health Policy Research Unit COVID-19 Co-Production Group), University College London, London, UK
5. School of Social Policy/ Institute for Mental Health, University of Birmingham, Birmingham, UK
6. Population Health Research Institute, St George’s, University of London, London, UK
7. Department of Mental Health Nursing, Florence Nightingale Faculty of Nursing, Midwifery & Palliative Care, King’s College London, London, UK
8. Centre for Mental Health, London, UK
9. Anna Freud Centre, London, UK
10. North East London NHS Foundation Trust, London, UK
11. Division of Psychology and Language Sciences, University College London, London, UK
12. Faculty of Education, Health and Human Sciences, University of Greenwich, London, UK
13. South London and Maudsley NHS Foundation Trust, London, UK
14. National Elf Service Ltd, Oxford, UK

Contents

[Introduction: 4](#_Toc47955754)

[Table 1: Participant demographics (n=2,180) 5](#_Toc47955755)

[Table 2: Current work challenges for all participants by setting*, in order of % rated ‘Very’ and ‘Extremely relevant’ for ‘All settings’ (n=2,180) 7](#_Toc47955756)

[Table 3: Current work challenges specific to participants working in inpatient services and crisis houses (questions only asked in these settings) in order of % rated ‘Very’ and ‘Extremely relevant’ (n=644) 12](#_Toc47955757)

[Table 4: Current work challenges specific to participants working in residential services, in order of % rated ‘Very’ and ‘Extremely relevant’ (n=79) 14](#_Toc47955758)

[Table 5: Current work challenges specific to participants working in crisis assessment services, in order of % rated ‘Very’ and ‘Extremely relevant’ combined (n=308) 16](#_Toc47955759)

[Table 6: Current work challenges specific to participants working in community teams and psychological treatment services, in order of % rated ‘Very’ and ‘Extremely relevant’ combined (n=1,268) 18](#_Toc47955760)

[Table 7: What are the main challenges for infection control for participants working in inpatient and residential services? (Open-ended question) 20](#_Toc47955761)

[Table 8: What are the main challenges for infection control for participants working in community settings? (Open-ended question) 24](#_Toc47955762)

[Table 9: Service activity* (responses from individual staff not individual services) (n=2,180) 27](#_Toc47955763)

[Table 10: Staff perspectives on service users’ and carers’ problems by service user group, in order of % rated ‘Very’ and ‘Extremely relevant’ combined for ‘All service user groups’* (n=2,180) 28](#_Toc47955764)

[Table 11: Groups of service users and carers as of particular concern during the COVID-19 pandemic period (open-ended questions) 32](#_Toc47955765)

[Table 12: Are you seeing any mental health problems that seem to arise directly from the current pandemic? (Open-ended question) 41](#_Toc47955766)

[Table 13: Sources of help in coping with COVID-19 at work by profession, in order of % rated ‘Very’ and ‘Extremely important’ combined for ‘All professions’ (n=2,180) 43](#_Toc47955767)

[Table 14: Sources of help by managerial role, in order of % rated ‘Very’ and ‘Extremely important’ combined for ‘All roles’ (n=2,180) 46](#_Toc47955768)

[Table 15: Staff-reported service changes 48](#_Toc47955769)

[Table 16: What are the main innovations and changes made to services as a result of the pandemic? (Open-ended questions) 50](#_Toc47955770)

[Table 17: Remote working views by setting* (n=1,443) 58](#_Toc47955771)

[Table 18: What is working well with remote working and technology use? (Open-ended question) 61](#_Toc47955772)

[Table 19: What’s Not Working with Remote Working? (Open-ended question) 65](#_Toc47955773)

[Table 20: Has any innovation or change been made in mental health care that you would like to remain in place after the pandemic subsides? (Open-ended question) 67](#_Toc47955774)

[Table 21: Are you particularly concerned about any potential long-term consequences from the current pandemic? (Open-ended question) 70](#_Toc47955775)

[Table 22: Top five rated current work challenges* for participants in each speciality** 75](#_Toc47955776)

[Table 23: Current work challenges specific to participants working with older adults (including dementia), in order of % rated ‘Very’ and ‘Extremely relevant’ combined (n=853) 77](#_Toc47955777)

[Table 24: Current work challenges specific to participants working with children and adolescents, in order of % rated ‘Very’ and ‘Extremely relevant’ combined (n=443) 78](#_Toc47955778)

[Table 25: Current work challenges for participants working in perinatal services, in order of rated % ‘Very’ and ‘Extremely relevant’ combined (n=363) 79](#_Toc47955779)

[Table 26: Current work challenges for participants working in forensic services, in order of % rated ‘Very’ and ‘Extremely relevant’ combined (n=365) 80](#_Toc47955780)

[Table 27: Current work challenges for participants working with people with intellectual disabilities, in order of % rated ‘Very’ and ‘Extremely relevant’ combined (n=648) 81](#_Toc47955781)

[Table 28: Current work challenges for participants working with people with eating disorders, in order of % rated ‘Very’ and ‘Extremely relevant’ combined (n=451) 82](#_Toc47955782)

[Table 29: Current work challenges for all participants by profession, in order of % rated ‘Very’ and ‘Extremely relevant’ combined for ‘All professions’ (n=2,180) 83](#_Toc47955783)

[Table 30: Current work challenges for all participants by managerial role, in order of % rated ‘Very’ and ‘Extremely relevant’ combined for ‘All roles’ (n=2,180) 88](#_Toc47955784)

# Introduction:

This report is a supplement to the article: ‘Impact on mental health care and on mental health service users of the COVID-19 pandemic: a mixed methods survey of UK mental health care staff’, DOI: 10.1007/s00127-020-01927-4. It presents in greater detail the results of a rapid online survey of UK mental health care staff on the impact of the COVID-19 pandemic and the associated restrictions on mental health care services, and on service users and carers. It was conducted by the [NIHR Mental Health Policy Research Unit](https://www.ucl.ac.uk/psychiatry/research/mental-health-policy-research-unit); it was launched 22 April 2020 and closed 12 May 2020.

The survey included a mixture of structured and open-ended questions and started with a set of questions for all participants, followed by branching questions to elicit further details from staff in specific settings and specialisms. A copy of the survey is available online (https://opinio.ucl.ac.uk/s?s=67819). The results from structured questions pertain to participants who completed at least one question from each of the three sections (Current work challenges, Service users and carers problems, and Sources of help) in the initial set of questions open to all participants (n=2,180). Results from the open-ended questions are based on all responses to relevant questions, using content analysis. For more details about the methods of this survey, including recruitment and analysis of the data, please see the article which this report supplements.

Some of the following results are presented by the type of ‘setting’ the participant works in. Each ‘setting’ is a group of services with similar work environments, grouped together primarily because infection control considerations are likely to be relatively similar. The following is a list of the settings used to describe work environments in this survey, and a short explanation of the types of service each setting includes:

- **Inpatient services and crisis houses:** Includes hospital and community based psychiatric wards, and crisis houses based in the community.
- **Residential services:** Includes all types of mental health supported accommodation.
- **Crisis assessment services:** Services whose main role is in assessing and managing crises in non-residential settings, including crisis/home treatment teams, liaison services and AMHP teams (Approved Mental Health Professional Teams).
- **Community teams:** Services mainly offering one-to-one mental health care in the community, including community mental health teams of all types, out-patient services, psychological treatment services, Individual Placement and Support services, voluntary services and any other non-crisis services that mainly provide one-to-one contacts.
- **Community groups:** Services mainly providing programmes at which service users attend together and mingle, including day or drop-in service, crisis cafes, recovery colleges, employment or activity services, or group programmes in the community.
- **Other:** All mental health works settings that do not fit within any of the above settings.

# Table 1: Participant demographics (n=2,180)

|  | | **n** | **%*** |
| --- | --- | --- | --- |
| Sector** (n=2,177) | NHS | 1,935 | 88.9 |
|  | Social care or other local government | 93 | 4.3 |
|  | Voluntary | 113 | 5.2 |
|  | Community or user-led organisation | 33 | 1.5 |
|  | Private | 86 | 4.0 |
| Current work setting** (n=2,174) | Inpatient | 623 | 28.7 |
|  | Crisis house | 31 | 1.4 |
|  | Residential service | 79 | 3.6 |
|  | Crisis assessment service | 308 | 14.2 |
|  | Community teams and psych. services | 1,268 | 58.3 |
|  | Community groups | 155 | 7.1 |
|  | Other | 249 | 11.5 |
| Is this your usual work setting? (n=2,172) | Yes | 2,024 | 93.2 |
|  | No - Locum/Trainee with no usual setting | 52 | 2.4 |
|  | No - Redeployed | 96 | 4.4 |
| Service user group** (n=2,174) | Working age adults | 1,521 | 70.0 |
|  | Older adults | 853 | 39.2 |
|  | People with drugs & alcohol problems | 456 | 21.0 |
|  | Children and adolescents | 443 | 20.4 |
|  | Forensic | 365 | 16.8 |
|  | Perinatal | 363 | 16.7 |
|  | People with intellectual disabilities | 648 | 29.8 |
|  | People with eating disorders | 451 | 20.7 |
|  | Other | 111 | 5.1 |
| Profession (n=2,172) | Psychologist | 347 | 16.0 |
|  | Nurse | 664 | 30.6 |
|  | Occupational therapist | 171 | 7.9 |
|  | Other qualified therapist | 189 | 8.7 |
|  | Peer support worker | 80 | 3.7 |
|  | Psychiatrist | 254 | 11.7 |
|  | Social worker | 97 | 4.5 |
|  | Manager, no professional qualification | 63 | 2.9 |
|  | Other | 307 | 14.1 |
| Are you a trainee/student? (n=2,148) | Yes | 146 | 6.8 |
|  | No | 2,002 | 93.2 |
| Role (n=2,176) | Manager or lead clinician | 826 | 38.0 |
|  | Not a manager or lead clinician | 1,350 | 62.0 |
| Years spent working in mental health (n=2,115) | Mean, SD | 14.9 | 10.5 |
| Country (n=2,175) | England | 1,814 | 83.4 |
|  | N Ireland | 22 | 1.0 |
|  | Scotland | 228 | 10.5 |
|  | Wales | 98 | 4.5 |
|  | Other | 13 | 0.6 |

| **Demographics (n=2,180)** |  | **n** | **%*** |
| --- | --- | --- | --- |
| Region: England (n=1,813) | East Midlands | 88 | 4.9 |
|  | East of England | 123 | 6.8 |
|  | London | 639 | 35.3 |
|  | North East | 70 | 3.9 |
|  | North West | 328 | 18.1 |
|  | South East | 265 | 14.6 |
|  | South West | 119 | 6.6 |
|  | West Midlands | 109 | 6.0 |
|  | Yorkshire and The Humber | 72 | 4.0 |
| Locality (n=2,161) | City/town >100,000 pop | 1,623 | 75.1 |
|  | City/town <100,000 pop | 374 | 17.3 |
|  | Rural | 164 | 7.6 |
| Gender (n=1,723) | Male | 341 | 19.8 |
|  | Female | 1,378 | 80.0 |
|  | Other | 4 | 0.2 |
| Age (n=1,714) | Under 25 | 64 | 3.7 |
|  | 25-34 | 385 | 22.5 |
|  | 35-44 | 411 | 24.0 |
|  | 45-54 | 535 | 31.2 |
|  | 55-64 | 298 | 17.4 |
|  | 65 or over | 21 | 1.2 |
| Ethnicity (n=1,647) | White | 1,433 | 87.0 |
|  | Asian | 92 | 5.6 |
|  | Black | 61 | 3.7 |
|  | Mixed/Multiple ethnic groups | 51 | 3.1 |
|  | Other | 10 | 0.6 |
| Caring responsibilities for children (n=1,745) | Yes | 628 | 36.0 |
|  | No | 1,117 | 64.0 |
| Caring responsibilities for elderly or disabled relatives or friends (n=1,737) | Yes | 390 | 22.5 |
|  | No | 1347 | 77.6 |
| Current working situation (n=1,747) | Sick or self-isolating | 32 | 1.8 |
|  | Working from home | 522 | 29.9 |
|  | Mixture of workplace and working from home | 578 | 33.1 |
|  | Mainly based at workplace | 615 | 35.2 |
| COVID-19 status (n=1,743) | Yes, confirmed | 29 | 1.7 |
|  | Yes, suspected | 454 | 26.1 |
|  | No, not suspected | 1,260 | 72.3 |
| COVID-19 status of household members (n=1,740) | Yes, confirmed | 16 | 0.9 |
|  | Yes, suspected | 394 | 22.6 |
|  | No, not suspected | 1,330 | 76.4 |
| *Percentages are of participants that provided an answer. The variable with the highest number of missing responses was ‘Ethnicity (n=1,647)’, where there were 533 missing responses (24.4%).  **Participants may work across more than one sector (e.g. NHS and voluntary), in more than one setting (e.g. an inpatient service and crisis assessment service) and with more than one patient group (e.g. working age adults and forensic). Percentages for these variables therefore do not add to 100%. | | | |

# Table 2: Current work challenges for all participants by setting*, in order of % rated ‘Very’ and ‘Extremely relevant’ for ‘All settings’ (n=2,180)

|  | | **Inpatient services and crisis houses (n=644)** | | **Residential services (n=79)** | | **Crisis assessment services (n=308)** | | **Community teams** (n=1,268)** | | **Community groups (n=155)** | | **Other (n=249)** | | **All settings (n= 2,180)** | |
| --- | --- | --- | --- | --- | --- | --- | --- | --- | --- | --- | --- | --- | --- | --- | --- |
|  |  | **n** | **%***** | **n** | **%** | **n** | **%** | **n** | **%** | **n** | **%** | **n** | **%** | **n** | **%** |
| Having to adapt too quickly to new ways of working | 1. Not relevant | 25 | 3.9 | 7 | 8.9 | 17 | 5.6 | 61 | 4.8 | 13 | 8.4 | 23 | 9.4 | 112 | 5.2 |
|  | 2. Slightly | 103 | 16.1 | 18 | 22.8 | 58 | 19.0 | 240 | 19.0 | 26 | 16.8 | 55 | 22.4 | 389 | 18.0 |
|  | 3. Moderately | 132 | 20.6 | 18 | 22.8 | 71 | 23.2 | 266 | 21.1 | 29 | 18.7 | 48 | 19.5 | 452 | 20.9 |
|  | 4. Very | 170 | 26.5 | 16 | 20.3 | 66 | 21.6 | 293 | 23.2 | 32 | 20.7 | 59 | 24.0 | 539 | 24.9 |
|  | 5. Extremely relevant | 211 | 32.9 | 20 | 25.3 | 94 | 30.7 | 401 | 31.8 | 55 | 35.5 | 61 | 24.8 | 673 | 31.1 |
| The risk I or my colleagues could be infected with COVID-19 at work | 1. Not relevant | 16 | 2.5 | 8 | 10.3 | 14 | 4.6 | 229 | 18.2 | 42 | 27.1 | 60 | 24.4 | 309 | 14.3 |
|  | 2. Slightly | 70 | 10.9 | 14 | 18.0 | 47 | 15.3 | 324 | 25.7 | 33 | 21.3 | 59 | 24.0 | 446 | 20.6 |
|  | 3. Moderately | 168 | 26.2 | 18 | 23.1 | 92 | 29.9 | 325 | 25.8 | 30 | 19.4 | 54 | 22.0 | 540 | 24.9 |
|  | 4. Very | 156 | 24.3 | 20 | 25.6 | 85 | 27.6 | 223 | 17.7 | 26 | 16.8 | 30 | 12.2 | 416 | 19.2 |
|  | 5. Extremely relevant | 231 | 36.0 | 18 | 23.1 | 70 | 22.7 | 160 | 12.7 | 24 | 15.5 | 43 | 17.5 | 456 | 21.0 |
| Having to respond to additional mental health needs that appear to result from COVID-19 | 1. Not relevant | 74 | 11.5 | 6 | 7.6 | 20 | 6.5 | 93 | 7.4 | 7 | 4.5 | 45 | 18.2 | 213 | 9.8 |
|  | 2. Slightly | 147 | 22.9 | 17 | 21.5 | 75 | 24.4 | 304 | 24.1 | 35 | 22.6 | 74 | 29.8 | 519 | 23.9 |
|  | 3. Moderately | 172 | 26.8 | 26 | 32.9 | 98 | 31.9 | 359 | 28.4 | 38 | 24.5 | 58 | 23.4 | 578 | 26.6 |
|  | 4. Very | 112 | 17.5 | 19 | 24.1 | 60 | 19.5 | 272 | 21.5 | 40 | 25.8 | 38 | 15.3 | 445 | 20.5 |
|  | 5. Extremely relevant | 137 | 21.3 | 11 | 13.9 | 54 | 17.6 | 235 | 18.6 | 35 | 22.6 | 33 | 13.3 | 416 | 19.2 |
| Pressures resulting from the need to support colleagues through the stresses associated with the pandemic | 1. Not relevant | 40 | 6.3 | 9 | 11.5 | 18 | 5.9 | 130 | 10.3 | 13 | 8.4 | 35 | 14.3 | 215 | 9.9 |
|  | 2. Slightly | 138 | 21.6 | 15 | 19.2 | 78 | 25.4 | 354 | 28.1 | 32 | 20.7 | 72 | 29.4 | 551 | 25.5 |
|  | 3. Moderately | 153 | 23.9 | 19 | 24.4 | 80 | 26.1 | 308 | 24.5 | 43 | 27.7 | 68 | 27.8 | 546 | 25.3 |
|  | 4. Very | 142 | 22.2 | 19 | 24.4 | 74 | 24.1 | 269 | 21.4 | 32 | 20.7 | 43 | 17.6 | 462 | 21.4 |
|  | 5. Extremely relevant | 167 | 26.1 | 16 | 20.5 | 57 | 18.6 | 197 | 15.7 | 35 | 22.6 | 27 | 11.0 | 388 | 18.0 |
| Service users no longer getting an acceptable service due to service reconfiguration because of COVID-19 | 1. Not relevant | 108 | 16.9 | 10 | 12.7 | 41 | 13.5 | 155 | 12.3 | 16 | 10.4 | 44 | 17.7 | 313 | 14.5 |
|  | 2. Slightly | 143 | 22.4 | 16 | 20.3 | 70 | 23.0 | 302 | 24.0 | 26 | 16.9 | 56 | 22.6 | 496 | 22.9 |
|  | 3. Moderately | 158 | 24.7 | 25 | 31.7 | 81 | 26.6 | 317 | 25.1 | 39 | 25.3 | 54 | 21.8 | 540 | 24.9 |
|  | 4. Very | 132 | 20.7 | 17 | 21.5 | 70 | 23.0 | 265 | 21.0 | 30 | 19.5 | 53 | 21.4 | 446 | 20.6 |
|  | 5. Extremely relevant | 98 | 15.3 | 11 | 13.9 | 42 | 13.8 | 222 | 17.6 | 43 | 27.9 | 41 | 16.5 | 371 | 17.1 |
| Having to learn to use new technologies too quickly and/or without sufficient training and support | 1. Not relevant | 126 | 19.6 | 27 | 34.6 | 56 | 18.4 | 158 | 12.5 | 26 | 17.0 | 61 | 24.7 | 355 | 16.4 |
|  | 2. Slightly | 152 | 23.7 | 18 | 23.1 | 71 | 23.3 | 306 | 24.3 | 27 | 17.7 | 67 | 27.1 | 517 | 23.9 |
|  | 3. Moderately | 151 | 23.5 | 15 | 19.2 | 72 | 23.6 | 282 | 22.4 | 42 | 27.5 | 42 | 17.0 | 485 | 22.4 |
|  | 4. Very | 114 | 17.8 | 16 | 20.5 | 58 | 19.0 | 266 | 21.1 | 28 | 18.3 | 50 | 20.2 | 420 | 19.4 |
|  | 5. Extremely relevant | 99 | 15.4 | 2 | 2.6 | 48 | 15.7 | 249 | 19.8 | 30 | 19.6 | 27 | 10.9 | 389 | 18.0 |
| **Current work challenges for participants by setting*, in order of % rated ‘Very’ and ‘Extremely relevant’ for ‘All settings’ (n=2,180)** | | **Inpatient services (incl. crisis house) (n=644)** | | **Residential services (n=79)** | | **Crisis assessment services (n=308)** | | **Community teams** (n=1,268)** | | **Community groups (n=155)** | | **Other (n=249)** | | **All settings (n= 2,180)** | |
|  |  | **n** | **%***** | **n** | **%** | **n** | **%** | **n** | **%** | **n** | **%** | **n** | **%** | **n** | **%** |
| The risk family and friends may be infected with COVID-19 through me | 1. Not relevant | 47 | 7.3 | 11 | 13.9 | 30 | 9.7 | 317 | 25.1 | 50 | 32.7 | 72 | 29.3 | 436 | 20.1 |
|  | 2. Slightly | 110 | 17.2 | 17 | 21.5 | 64 | 20.8 | 332 | 26.3 | 34 | 22.2 | 71 | 28.9 | 503 | 23.2 |
|  | 3. Moderately | 144 | 22.5 | 14 | 17.7 | 81 | 26.3 | 276 | 21.8 | 26 | 17.0 | 36 | 14.6 | 458 | 21.1 |
|  | 4. Very | 136 | 21.2 | 20 | 25.3 | 68 | 22.1 | 173 | 13.7 | 18 | 11.8 | 30 | 12.2 | 348 | 16.1 |
|  | 5. Extremely relevant | 204 | 31.8 | 17 | 21.5 | 65 | 21.1 | 166 | 13.1 | 25 | 16.3 | 37 | 15.0 | 423 | 19.5 |
| The risk that COVID-19 will spread between service users I'm working with | 1. Not relevant | 33 | 5.1 | 7 | 8.9 | 52 | 17.1 | 478 | 37.9 | 57 | 36.8 | 91 | 36.8 | 619 | 28.5 |
|  | 2. Slightly | 70 | 10.9 | 11 | 13.9 | 80 | 26.2 | 281 | 22.3 | 28 | 18.1 | 42 | 17.0 | 403 | 18.6 |
|  | 3. Moderately | 120 | 18.7 | 20 | 25.3 | 78 | 25.6 | 222 | 17.6 | 29 | 18.7 | 31 | 12.6 | 378 | 17.4 |
|  | 4. Very | 163 | 25.4 | 19 | 24.1 | 54 | 17.7 | 144 | 11.4 | 18 | 11.6 | 39 | 15.8 | 342 | 15.8 |
|  | 5. Extremely relevant | 257 | 40.0 | 22 | 27.9 | 41 | 13.4 | 136 | 10.8 | 23 | 14.8 | 44 | 17.8 | 427 | 19.7 |
| Being expected to use new technologies without reliable access to necessary tools and equipment | 1. Not relevant | 174 | 27.2 | 35 | 44.3 | 65 | 21.2 | 256 | 20.3 | 39 | 25.2 | 73 | 29.7 | 524 | 24.2 |
|  | 2. Slightly | 149 | 23.3 | 16 | 20.3 | 77 | 25.2 | 292 | 23.1 | 29 | 18.7 | 67 | 27.2 | 510 | 23.6 |
|  | 3. Moderately | 126 | 19.7 | 13 | 16.5 | 62 | 20.3 | 254 | 20.1 | 33 | 21.3 | 39 | 15.9 | 415 | 19.2 |
|  | 4. Very | 98 | 15.3 | 8 | 10.1 | 58 | 19.0 | 227 | 18.0 | 27 | 17.4 | 37 | 15.0 | 359 | 16.6 |
|  | 5. Extremely relevant | 93 | 14.5 | 7 | 8.9 | 44 | 14.4 | 233 | 18.5 | 27 | 17.4 | 30 | 12.2 | 357 | 16.5 |
| Problems resulting from lack of access to testing | 1. Not relevant | 103 | 16.0 | 21 | 26.6 | 59 | 19.4 | 405 | 32.2 | 63 | 41.2 | 93 | 37.5 | 614 | 28.4 |
|  | 2. Slightly | 112 | 17.5 | 15 | 19.0 | 76 | 25.0 | 266 | 21.1 | 23 | 15.0 | 48 | 19.4 | 427 | 19.7 |
|  | 3. Moderately | 136 | 21.2 | 8 | 10.1 | 70 | 23.0 | 242 | 19.2 | 32 | 20.9 | 36 | 14.5 | 416 | 19.2 |
|  | 4. Very | 130 | 20.3 | 12 | 15.2 | 52 | 17.1 | 169 | 13.4 | 16 | 10.5 | 32 | 12.9 | 327 | 15.1 |
|  | 5. Extremely relevant | 161 | 25.1 | 23 | 29.1 | 47 | 15.5 | 177 | 14.1 | 19 | 12.4 | 39 | 15.7 | 382 | 17.6 |
| Concern that physical health care received by service users I work with may not be adequate | 1. Not relevant | 126 | 19.7 | 21 | 26.6 | 52 | 17.2 | 287 | 22.8 | 39 | 25.3 | 78 | 31.6 | 505 | 23.4 |
|  | 2. Slightly | 140 | 21.8 | 15 | 19.0 | 64 | 21.1 | 279 | 22.2 | 23 | 14.9 | 42 | 17.0 | 461 | 21.3 |
|  | 3. Moderately | 140 | 21.8 | 15 | 19.0 | 76 | 25.1 | 299 | 23.8 | 37 | 24.0 | 57 | 23.1 | 492 | 22.8 |
|  | 4. Very | 122 | 19.0 | 16 | 20.3 | 66 | 21.8 | 230 | 18.3 | 31 | 20.1 | 38 | 15.4 | 407 | 18.8 |
|  | 5. Extremely relevant | 113 | 17.6 | 12 | 15.2 | 45 | 14.9 | 162 | 12.9 | 24 | 15.6 | 32 | 13.0 | 297 | 13.7 |
| Difficulty putting infection control measures into practice in the setting I work in | 1. Not relevant | 87 | 13.5 | 19 | 24.1 | 59 | 19.2 | 437 | 34.6 | 67 | 43.2 | 107 | 43.2 | 642 | 29.5 |
|  | 2. Slightly | 114 | 17.7 | 17 | 21.5 | 70 | 22.8 | 298 | 23.6 | 25 | 16.1 | 50 | 20.2 | 463 | 21.3 |
|  | 3. Moderately | 128 | 19.9 | 12 | 15.2 | 75 | 24.4 | 237 | 18.8 | 24 | 15.5 | 32 | 12.9 | 404 | 18.6 |
|  | 4. Very | 131 | 20.3 | 22 | 27.9 | 65 | 21.2 | 169 | 13.4 | 22 | 14.2 | 26 | 10.5 | 331 | 15.2 |
|  | 5. Extremely relevant | 184 | 28.6 | 9 | 11.4 | 38 | 12.4 | 122 | 9.7 | 17 | 11.0 | 33 | 13.3 | 333 | 15.3 |

| **Current work challenges for participants by setting*, in order of % rated ‘Very’ and ‘Extremely relevant’ for ‘All settings’ (n=2,180)** | | **Inpatient services (incl. crisis house) (n=644)** | | **Residential services (n=79)** | | **Crisis assessment services (n=308)** | | **Community teams** (n=1,268)** | | **Community groups (n=155)** | | **Other (n=249)** | | **All settings (n= 2,180)** | |
| --- | --- | --- | --- | --- | --- | --- | --- | --- | --- | --- | --- | --- | --- | --- | --- |
|  |  | **n** | **%***** | **n** | **%** | **n** | **%** | **n** | **%** | **n** | **%** | **n** | **%** | **n** | **%** |
| Greater workload than usual | 1. Not relevant | 124 | 19.3 | 21 | 26.6 | 86 | 27.9 | 343 | 27.1 | 37 | 23.9 | 83 | 33.3 | 587 | 26.9 |
|  | 2. Slightly | 97 | 15.1 | 7 | 8.9 | 61 | 19.8 | 247 | 19.5 | 20 | 12.9 | 36 | 14.5 | 381 | 17.5 |
|  | 3. Moderately | 180 | 28.0 | 19 | 24.1 | 64 | 20.8 | 342 | 27.0 | 36 | 23.2 | 68 | 27.3 | 590 | 27.1 |
|  | 4. Very | 148 | 23.0 | 16 | 20.3 | 56 | 18.2 | 199 | 15.7 | 30 | 19.4 | 46 | 18.5 | 375 | 17.2 |
|  | 5. Extremely relevant | 95 | 14.8 | 16 | 20.3 | 41 | 13.3 | 137 | 10.8 | 32 | 20.7 | 16 | 6.4 | 247 | 11.3 |
| Increased difficulty managing work-life balance, for example because of loss of childcare | 1. Not relevant | 311 | 48.4 | 49 | 62.8 | 132 | 43.3 | 481 | 38.1 | 66 | 42.9 | 137 | 55.0 | 940 | 43.2 |
|  | 2. Slightly | 101 | 15.7 | 8 | 10.3 | 55 | 18.0 | 225 | 17.8 | 17 | 11.0 | 50 | 20.1 | 381 | 17.5 |
|  | 3. Moderately | 75 | 11.7 | 10 | 12.8 | 45 | 14.8 | 203 | 16.1 | 23 | 14.9 | 25 | 10.0 | 301 | 13.9 |
|  | 4. Very | 74 | 11.5 | 4 | 5.1 | 39 | 12.8 | 171 | 13.5 | 18 | 11.7 | 19 | 7.6 | 261 | 12.0 |
|  | 5. Extremely relevant | 81 | 12.6 | 7 | 9.0 | 34 | 11.2 | 184 | 14.6 | 30 | 19.5 | 18 | 7.2 | 291 | 13.4 |
| Lack of protective clothing (PPE) and equipment needed for infection control | 1. Not relevant | 123 | 19.1 | 22 | 29.0 | 82 | 26.7 | 494 | 39.1 | 71 | 46.1 | 114 | 46.0 | 758 | 34.9 |
|  | 2. Slightly | 141 | 21.9 | 16 | 21.1 | 68 | 22.2 | 291 | 23.0 | 21 | 13.6 | 48 | 19.4 | 477 | 22.0 |
|  | 3. Moderately | 152 | 23.6 | 17 | 22.4 | 77 | 25.1 | 213 | 16.9 | 22 | 14.3 | 29 | 11.7 | 396 | 18.3 |
|  | 4. Very | 96 | 14.9 | 11 | 14.5 | 47 | 15.3 | 142 | 11.2 | 22 | 14.3 | 29 | 11.7 | 265 | 12.2 |
|  | 5. Extremely relevant | 131 | 20.4 | 10 | 13.2 | 33 | 10.8 | 124 | 9.8 | 18 | 11.7 | 28 | 11.3 | 274 | 12.6 |
| Safeguarding and other risk management processes cannot be adequately mobilised due to limited social care, legal or police response | 1. Not relevant | 184 | 28.8 | 21 | 26.6 | 58 | 19.1 | 327 | 25.9 | 44 | 28.4 | 97 | 39.0 | 609 | 28.1 |
|  | 2. Slightly | 148 | 23.1 | 21 | 26.6 | 81 | 26.6 | 335 | 26.6 | 40 | 25.8 | 64 | 25.7 | 550 | 25.4 |
|  | 3. Moderately | 141 | 22.0 | 22 | 27.9 | 79 | 26.0 | 280 | 22.2 | 26 | 16.8 | 34 | 13.7 | 471 | 21.7 |
|  | 4. Very | 98 | 15.3 | 7 | 8.9 | 58 | 19.1 | 203 | 16.1 | 27 | 17.4 | 30 | 12.1 | 333 | 15.4 |
|  | 5. Extremely relevant | 69 | 10.8 | 8 | 10.1 | 28 | 9.2 | 117 | 9.3 | 18 | 11.6 | 24 | 9.6 | 205 | 9.5 |
| Staff shortages (more than is usual in this setting) | 1. Not relevant | 131 | 20.5 | 23 | 30.3 | 66 | 21.6 | 435 | 34.5 | 50 | 32.5 | 105 | 42.2 | 690 | 31.8 |
|  | 2. Slightly | 146 | 22.8 | 16 | 21.1 | 84 | 27.5 | 362 | 28.7 | 39 | 25.3 | 65 | 26.1 | 572 | 26.4 |
|  | 3. Moderately | 149 | 23.3 | 11 | 14.5 | 79 | 25.9 | 239 | 19.0 | 24 | 15.6 | 35 | 14.1 | 419 | 19.3 |
|  | 4. Very | 98 | 15.3 | 11 | 14.5 | 45 | 14.8 | 132 | 10.5 | 22 | 14.3 | 26 | 10.4 | 258 | 11.9 |
|  | 5. Extremely relevant | 116 | 18.1 | 15 | 19.7 | 31 | 10.2 | 93 | 7.4 | 19 | 12.3 | 18 | 7.2 | 228 | 10.5 |
| Working longer hours than usual | 1. Not relevant | 219 | 34.0 | 28 | 35.4 | 108 | 35.3 | 482 | 38.0 | 52 | 33.6 | 115 | 46.2 | 842 | 38.7 |
|  | 2. Slightly | 95 | 14.8 | 16 | 20.3 | 56 | 18.3 | 241 | 19.0 | 21 | 13.6 | 40 | 16.1 | 390 | 17.9 |
|  | 3. Moderately | 153 | 23.8 | 13 | 16.5 | 64 | 20.9 | 287 | 22.7 | 28 | 18.1 | 54 | 21.7 | 487 | 22.4 |
|  | 4. Very | 99 | 15.4 | 10 | 12.7 | 36 | 11.8 | 145 | 11.4 | 23 | 14.8 | 25 | 10.0 | 255 | 11.7 |
|  | 5. Extremely relevant | 78 | 12.1 | 12 | 15.2 | 42 | 13.7 | 112 | 8.8 | 31 | 20.0 | 15 | 6.0 | 204 | 9.4 |

| **Current work challenges for participants by setting*, in order of % rated ‘Very’ and ‘Extremely relevant’ for ‘All settings’ (n=2,180)** | | **Inpatient services (incl. crisis house) (n=644)** | | **Residential services (n=79)** | | **Crisis assessment services (n=308)** | | **Community teams** (n=1,268)** | | **Community groups (n=155)** | | **Other (n=249)** | | **All settings (n= 2,180)** | |
| --- | --- | --- | --- | --- | --- | --- | --- | --- | --- | --- | --- | --- | --- | --- | --- |
|  |  | **n** | **%***** | **n** | **%** | **n** | **%** | **n** | **%** | **n** | **%** | **n** | **%** | **n** | **%** |
| Working in a different setting or with different clinical problems from usual | 1. Not relevant | 301 | 46.9 | 43 | 55.1 | 137 | 44.8 | 570 | 45.2 | 57 | 36.8 | 122 | 49.0 | 1,008 | 46.5 |
|  | 2. Slightly | 98 | 15.3 | 11 | 14.1 | 59 | 19.3 | 245 | 19.4 | 34 | 21.9 | 50 | 20.1 | 381 | 17.6 |
|  | 3. Moderately | 90 | 14.0 | 14 | 18.0 | 45 | 14.7 | 202 | 16.0 | 31 | 20.0 | 24 | 9.6 | 324 | 14.9 |
|  | 4. Very | 82 | 12.8 | 6 | 7.7 | 38 | 12.4 | 138 | 11.0 | 25 | 16.1 | 31 | 12.5 | 257 | 11.9 |
|  | 5. Extremely relevant | 71 | 11.1 | 4 | 5.1 | 27 | 8.8 | 105 | 8.3 | 8 | 5.2 | 22 | 8.8 | 198 | 9.1 |
| Feeling less able to do my job than usual because my own well-being has suffered through the stresses of the pandemic | 1. Not relevant | 199 | 31.1 | 28 | 35.9 | 109 | 35.6 | 349 | 27.7 | 52 | 33.6 | 93 | 37.8 | 651 | 30.1 |
|  | 2. Slightly | 197 | 30.8 | 21 | 26.9 | 90 | 29.4 | 411 | 32.6 | 39 | 25.2 | 85 | 34.6 | 681 | 31.4 |
|  | 3. Moderately | 118 | 18.4 | 14 | 18.0 | 63 | 20.6 | 250 | 19.8 | 32 | 20.7 | 39 | 15.9 | 413 | 19.1 |
|  | 4. Very | 61 | 9.5 | 12 | 15.4 | 23 | 7.5 | 145 | 11.5 | 13 | 8.4 | 15 | 6.1 | 240 | 11.1 |
|  | 5. Extremely relevant | 65 | 10.2 | 3 | 3.9 | 21 | 6.9 | 105 | 8.3 | 19 | 12.3 | 14 | 5.7 | 181 | 8.4 |
| Feeling under pressure from managers or colleagues to be less cautious about infection control than I would like | 1. Not relevant | 305 | 47.5 | 40 | 50.6 | 166 | 54.1 | 805 | 63.5 | 101 | 65.6 | 163 | 66.3 | 1,282 | 59.0 |
|  | 2. Slightly | 97 | 15.1 | 17 | 21.5 | 52 | 16.9 | 179 | 14.1 | 15 | 9.7 | 36 | 14.6 | 322 | 14.8 |
|  | 3. Moderately | 79 | 12.3 | 8 | 10.1 | 28 | 9.1 | 118 | 9.3 | 14 | 9.1 | 21 | 8.5 | 216 | 9.9 |
|  | 4. Very | 73 | 11.4 | 11 | 13.9 | 32 | 10.4 | 78 | 6.2 | 11 | 7.1 | 9 | 3.7 | 163 | 7.5 |
|  | 5. Extremely relevant | 88 | 13.7 | 3 | 3.8 | 29 | 9.5 | 87 | 6.9 | 13 | 8.4 | 17 | 6.9 | 190 | 8.7 |
| Not enough of the team I'm working with are permanently employed in this setting (lots of bank/locum and redeployed staff) | 1. Not relevant | 260 | 40.6 | 40 | 50.6 | 164 | 53.4 | 908 | 72.0 | 111 | 71.6 | 201 | 81.1 | 1,376 | 63.5 |
|  | 2. Slightly | 137 | 21.4 | 16 | 20.3 | 73 | 23.8 | 189 | 15.0 | 16 | 10.3 | 19 | 7.7 | 351 | 16.2 |
|  | 3. Moderately | 98 | 15.3 | 5 | 6.3 | 39 | 12.7 | 85 | 6.7 | 14 | 9.0 | 15 | 6.1 | 191 | 8.8 |
|  | 4. Very | 74 | 11.5 | 11 | 13.9 | 15 | 4.9 | 39 | 3.1 | 9 | 5.8 | 7 | 2.8 | 129 | 6.0 |
|  | 5. Extremely relevant | 72 | 11.2 | 7 | 8.9 | 16 | 5.2 | 40 | 3.2 | 5 | 3.2 | 6 | 2.4 | 121 | 5.6 |
| Pressure to accept redeployment to a setting where I don't feel happy to work | 1. Not relevant | 452 | 70.5 | 62 | 78.5 | 223 | 72.6 | 823 | 65.4 | 108 | 70.1 | 183 | 73.8 | 1,472 | 67.9 |
|  | 2. Slightly | 76 | 11.9 | 5 | 6.3 | 32 | 10.4 | 201 | 16.0 | 17 | 11.0 | 32 | 12.9 | 300 | 13.8 |
|  | 3. Moderately | 42 | 6.6 | 6 | 7.6 | 21 | 6.8 | 110 | 8.7 | 14 | 9.1 | 20 | 8.1 | 175 | 8.1 |
|  | 4. Very | 29 | 4.5 | 5 | 6.3 | 15 | 4.9 | 51 | 4.1 | 7 | 4.6 | 6 | 2.4 | 97 | 4.5 |
|  | 5. Extremely relevant | 42 | 6.6 | 1 | 1.3 | 16 | 5.2 | 74 | 5.9 | 8 | 5.2 | 7 | 2.8 | 124 | 5.7 |

| **Current work challenges for participants by setting*, in order of % rated ‘Very’ and ‘Extremely relevant’ for ‘All settings’ (n=2,180)** | | **Inpatient services (incl. crisis house) (n=644)** | | **Residential services (n=79)** | | **Crisis assessment services (n=308)** | | **Community teams** (n=1,268)** | | **Community groups (n=155)** | | **Other (n=249)** | | **All settings (n= 2,180)** | |
| --- | --- | --- | --- | --- | --- | --- | --- | --- | --- | --- | --- | --- | --- | --- | --- |
|  |  | **n** | **%***** | **n** | **%** | **n** | **%** | **n** |  | **n** | **%** | **n** | **%** | **n** | **%** |
| Problems commuting safely to work and back | 1. Not relevant | 469 | 72.8 | 52 | 65.8 | 240 | 77.9 | 1,000 | 78.9 | 119 | 76.8 | 190 | 76.3 | 1,684 | 77.3 |
|  | 2. Slightly | 52 | 8.1 | 9 | 11.4 | 30 | 9.7 | 121 | 9.5 | 12 | 7.7 | 25 | 10.0 | 190 | 8.7 |
|  | 3. Moderately | 52 | 8.1 | 8 | 10.1 | 19 | 6.2 | 60 | 4.7 | 10 | 6.5 | 16 | 6.4 | 126 | 5.8 |
|  | 4. Very | 38 | 5.9 | 7 | 8.9 | 11 | 3.6 | 43 | 3.4 | 7 | 4.5 | 8 | 3.2 | 89 | 4.1 |
|  | 5. Extremely relevant | 33 | 5.1 | 3 | 3.8 | 8 | 2.6 | 44 | 3.5 | 7 | 4.5 | 10 | 4.0 | 91 | 4.2 |
| *A participant may work in more than one setting (e.g. an inpatient service and a crisis assessment service) but will provide only one answer per challenge. | | | | | | | | | | | | | | | |
| **Includes psychological treatment services.  ***Percentages are of those who answered. The highest number of missing responses for a challenge was 18 (0.8% of total participants, n=2,180). This was for challenges: 'Pressures resulting from the need to support colleagues through the stresses associated with the pandemic' and 'Concern that physical health care received by service users I work with may not be adequate', where for each challenge n=2,162. | | | | | | | | | | | | | | | |

# Table 3: Current work challenges specific to participants working in inpatient services and crisis houses (questions only asked in these settings) in order of % rated ‘Very’ and ‘Extremely relevant’ (n=644)

|  | | **n** | **%*** |
| --- | --- | --- | --- |
| Lack of activities and facilities/increased boredom and agitation during COVID-19 pandemic (n=533) | 1. Not relevant | 38 | 7.1 |
|  | 2. Slightly | 56 | 10.5 |
|  | 3. Moderately | 101 | 19.0 |
|  | 4. Very | 114 | 21.4 |
|  | 5. Extremely relevant | 224 | 42.0 |
| Difficulty discharging people because services usually available in community are closed or less available (n=528) | 1. Not relevant | 83 | 15.7 |
|  | 2. Slightly | 54 | 10.2 |
|  | 3. Moderately | 88 | 16.7 |
|  | 4. Very | 124 | 23.5 |
|  | 5. Extremely relevant | 179 | 33.9 |
| Difficulty maintaining infection control because inpatients/residents are too unwell to follow procedures (n=532) | 1. Not relevant | 67 | 12.6 |
|  | 2. Slightly | 62 | 11.7 |
|  | 3. Moderately | 110 | 20.7 |
|  | 4. Very | 104 | 19.6 |
|  | 5. Extremely relevant | 189 | 35.5 |
| Challenges protecting people at high risk of severe COVID-19 infection adequately (n=529) | 1. Not relevant | 83 | 15.7 |
|  | 2. Slightly | 88 | 16.6 |
|  | 3. Moderately | 92 | 17.4 |
|  | 4. Very | 114 | 21.6 |
|  | 5. Extremely relevant | 152 | 28.7 |
| Difficulty discharging inpatients/residents because they will lack usual support from family and friends (n=524) | 1. Not relevant | 100 | 19.1 |
|  | 2. Slightly | 74 | 14.1 |
|  | 3. Moderately | 90 | 17.2 |
|  | 4. Very | 106 | 20.2 |
|  | 5. Extremely relevant | 154 | 29.4 |
| Concerns about deprivation of liberty in enforcing infection control (e.g. by isolating patients in their rooms) (n=534) | 1. Not relevant | 87 | 16.3 |
|  | 2. Slightly | 88 | 16.5 |
|  | 3. Moderately | 109 | 20.4 |
|  | 4. Very | 98 | 18.4 |
|  | 5. Extremely relevant | 152 | 28.5 |
| Difficulty maintaining infection control because inpatients/residents with confirmed/suspected COVID-19 cannot be effectively segregated from others in this environment (n=534) | 1. Not relevant | 118 | 22.1 |
|  | 2. Slightly | 90 | 16.9 |
|  | 3. Moderately | 92 | 17.2 |
|  | 4. Very | 94 | 17.6 |
|  | 5. Extremely relevant | 140 | 26.2 |
| Greater difficulty carrying out usual observations and/or managing disturbed behaviour in the context of COVID-19 (n=528) | 1. Not relevant | 99 | 18.8 |
|  | 2. Slightly | 105 | 19.9 |
|  | 3. Moderately | 122 | 23.1 |
|  | 4. Very | 86 | 16.3 |
|  | 5. Extremely relevant | 116 | 22.0 |
| Pressure to discharge more quickly than usual because of the pandemic (n=534) | 1. Not relevant | 166 | 31.1 |
|  | 2. Slightly | 94 | 17.6 |
|  | 3. Moderately | 71 | 13.3 |
|  | 4. Very | 78 | 14.6 |
|  | 5. Extremely relevant | 125 | 23.4 |
| Risk to staff because of lack of PPE (Personal Protective Equipment) (n=532) | 1. Not relevant | 111 | 20.9 |
|  | 2. Slightly | 116 | 21.8 |
|  | 3. Moderately | 103 | 19.4 |
|  | 4. Very | 69 | 13.0 |
|  | 5. Extremely relevant | 133 | 25.0 |

| **Current work challenges specific to participants working in inpatient services (including crisis houses), in order of % rated ‘Very’ and ‘Extremely relevant’ (n=644)** | | **n** | **%*** |
| --- | --- | --- | --- |
| Difficulty discharging people because of concerns that they may be an infection risk in their usual home (n=529) | 1. Not relevant | 178 | 33.7 |
|  | 2. Slightly | 85 | 16.1 |
|  | 3. Moderately | 98 | 18.5 |
|  | 4. Very | 72 | 13.6 |
|  | 5. Extremely relevant | 96 | 18.2 |
| Reduced access to advocacy and appeal processes under the Mental Health Act (n=532) | 1. Not relevant | 131 | 24.6 |
|  | 2. Slightly | 112 | 21.1 |
|  | 3. Moderately | 123 | 23.1 |
|  | 4. Very | 77 | 14.5 |
|  | 5. Extremely relevant | 89 | 16.7 |
| Difficulty meeting the physical health needs of people with confirmed/suspected COVID-19 on the ward (n=526) | 1. Not relevant | 150 | 28.5 |
|  | 2. Slightly | 101 | 19.2 |
|  | 3. Moderately | 118 | 22.4 |
|  | 4. Very | 82 | 15.6 |
|  | 5. Extremely relevant | 75 | 14.3 |
| Difficulty obtaining relevant advice or arranging assessment by or transfer to general medical services for physically unwell patients/residents (n=532) | 1. Not relevant | 197 | 37.0 |
|  | 2. Slightly | 98 | 18.4 |
|  | 3. Moderately | 97 | 18.2 |
|  | 4. Very | 82 | 15.4 |
|  | 5. Extremely relevant | 58 | 10.9 |
| *Percentages are of those who answered. The highest number of missing responses for a challenge was 100 (15.5% of total participants working in inpatient services, n=644). This was for challenge 'Difficulty discharging inpatients/residents because they will lack usual support from family and friends' where n=524. | | | |

# Table 4: Current work challenges specific to participants working in residential services, in order of % rated ‘Very’ and ‘Extremely relevant’ (n=79)

|  | | **n** | **%*** |
| --- | --- | --- | --- |
| More challenging environment because residents cannot go out and engage in activities as usual (n=63) | 1. Not relevant | 4 | 6.4 |
|  | 2. Slightly | 7 | 11.1 |
|  | 3. Moderately | 4 | 6.4 |
|  | 4. Very | 18 | 28.6 |
|  | 5. Extremely relevant | 30 | 47.6 |
| Not being able to have as much contact as usual with residents due to staff shortages or changes in service offered (n=63) | 1. Not relevant | 8 | 12.7 |
|  | 2. Slightly | 8 | 12.7 |
|  | 3. Moderately | 12 | 19.1 |
|  | 4. Very | 14 | 22.2 |
|  | 5. Extremely relevant | 21 | 33.3 |
| Difficulty maintaining infection control because people cannot be effectively segregated from one another in this environment (n=64) | 1. Not relevant | 5 | 7.8 |
|  | 2. Slightly | 14 | 21.9 |
|  | 3. Moderately | 12 | 18.8 |
|  | 4. Very | 16 | 25.0 |
|  | 5. Extremely relevant | 17 | 26.6 |
| Not being able to have as much contact as usual with residents due to quarantine precautions (n=64) | 1. Not relevant | 16 | 25.0 |
|  | 2. Slightly | 8 | 12.5 |
|  | 3. Moderately | 8 | 12.5 |
|  | 4. Very | 14 | 21.9 |
|  | 5. Extremely relevant | 18 | 28.1 |
| Lack of support due to reduction in other services in the community e.g. primary care, social care, voluntary sector services (n=62) | 1. Not relevant | 10 | 16.1 |
|  | 2. Slightly | 11 | 17.7 |
|  | 3. Moderately | 10 | 16.1 |
|  | 4. Very | 18 | 29.0 |
|  | 5. Extremely relevant | 13 | 21.0 |
| Difficulty managing communal areas of accommodation safely (n=62) | 1. Not relevant | 6 | 9.7 |
|  | 2. Slightly | 11 | 17.7 |
|  | 3. Moderately | 16 | 25.8 |
|  | 4. Very | 14 | 22.6 |
|  | 5. Extremely relevant | 15 | 24.2 |
| Difficulty maintaining infection control because residents don’t understand or are too unwell to follow procedures (n=63) | 1. Not relevant | 5 | 7.9 |
|  | 2. Slightly | 12 | 19.1 |
|  | 3. Moderately | 17 | 27.0 |
|  | 4. Very | 13 | 20.6 |
|  | 5. Extremely relevant | 16 | 25.4 |
| Lack of support because of closure of or reduction in community mental health services (n=62) | 1. Not relevant | 12 | 19.4 |
|  | 2. Slightly | 14 | 22.6 |
|  | 3. Moderately | 14 | 22.6 |
|  | 4. Very | 14 | 22.6 |
|  | 5. Extremely relevant | 8 | 12.9 |
| Challenges supporting residents who are very worried about COVID-19 infection (n=64) | 1. Not relevant | 11 | 17.2 |
|  | 2. Slightly | 19 | 29.7 |
|  | 3. Moderately | 14 | 21.9 |
|  | 4. Very | 13 | 20.3 |
|  | 5. Extremely relevant | 7 | 10.9 |

| **Current work challenges specific to participants working in residential services, in order of % rated ‘Very’ and ‘Extremely relevant’ (n=79)** | | **n** | **%*** |
| --- | --- | --- | --- |
| Difficulty getting appropriate medical care for residents who are ill with COVID-19 infections (n=64) | 1. Not relevant | 35 | 54.7 |
|  | 2. Slightly | 9 | 14.1 |
|  | 3. Moderately | 6 | 9.4 |
|  | 4. Very | 8 | 12.5 |
|  | 5. Extremely relevant | 6 | 9.4 |
| Lack of confidence and support in managing residents who are ill with COVID-19 infections (n=63) | 1. Not relevant | 25 | 39.7 |
|  | 2. Slightly | 14 | 22.2 |
|  | 3. Moderately | 13 | 20.6 |
|  | 4. Very | 5 | 7.9 |
|  | 5. Extremely relevant | 6 | 9.5 |
| *Percentages are of those who answered. The highest number of missing responses for a challenge was 15 (19.0% of participants working in residential services, n=79). This was for three separate challenges where n=62. | | | |

# Table 5: Current work challenges specific to participants working in crisis assessment services, in order of % rated ‘Very’ and ‘Extremely relevant’ combined (n=308)

|  | | **n** | **%*** |
| --- | --- | --- | --- |
| Not being able to signpost or refer to other services in your area (primary care, social care, voluntary sector services) (n=250) | 1. Not relevant | 22 | 8.8 |
|  | 2. Slightly | 41 | 16.4 |
|  | 3. Moderately | 51 | 20.4 |
|  | 4. Very | 52 | 20.8 |
|  | 5. Extremely relevant | 84 | 33.6 |
| Lack of continuing care from community mental health services (n=249) | 1. Not relevant | 39 | 15.7 |
|  | 2. Slightly | 47 | 18.9 |
|  | 3. Moderately | 59 | 23.7 |
|  | 4. Very | 45 | 18.1 |
|  | 5. Extremely relevant | 59 | 23.7 |
| Difficulty managing crises at home when no or few face to face contacts (n=249) | 1. Not relevant | 51 | 20.5 |
|  | 2. Slightly | 45 | 18.1 |
|  | 3. Moderately | 61 | 24.5 |
|  | 4. Very | 53 | 21.3 |
|  | 5. Extremely relevant | 39 | 15.7 |
| Difficulty assessing clients by phone or video call (n=249) | 1. Not relevant | 42 | 16.9 |
|  | 2. Slightly | 62 | 24.9 |
|  | 3. Moderately | 63 | 25.3 |
|  | 4. Very | 43 | 17.3 |
|  | 5. Extremely relevant | 39 | 15.7 |
| Lack of a base where clients can be seen face to face (n=252) | 1. Not relevant | 98 | 38.9 |
|  | 2. Slightly | 43 | 17.1 |
|  | 3. Moderately | 32 | 12.7 |
|  | 4. Very | 37 | 14.7 |
|  | 5. Extremely relevant | 42 | 16.7 |
| Difficulties engaging clients in remote appointments (n=250) | 1. Not relevant | 45 | 18.0 |
|  | 2. Slightly | 66 | 26.4 |
|  | 3. Moderately | 63 | 25.2 |
|  | 4. Very | 39 | 15.6 |
|  | 5. Extremely relevant | 37 | 14.8 |
| Lack of facility to continue mental health service safely in A and E departments (n=251) | 1. Not relevant | 86 | 34.3 |
|  | 2. Slightly | 48 | 19.1 |
|  | 3. Moderately | 41 | 16.3 |
|  | 4. Very | 37 | 14.7 |
|  | 5. Extremely relevant | 39 | 15.5 |
| Technological difficulties with remote appointments (n=251) | 1. Not relevant | 54 | 21.5 |
|  | 2. Slightly | 75 | 29.9 |
|  | 3. Moderately | 48 | 19.1 |
|  | 4. Very | 33 | 13.2 |
|  | 5. Extremely relevant | 41 | 16.3 |
| Problems ensuring clients have medication and that it is monitored regularly (n=251) | 1. Not relevant | 71 | 28.3 |
|  | 2. Slightly | 67 | 26.7 |
|  | 3. Moderately | 49 | 19.5 |
|  | 4. Very | 38 | 15.1 |
|  | 5. Extremely relevant | 26 | 10.4 |

| **Current work challenges specific to participants working in crisis assessment services, in order of % rated ‘Very’ and ‘Extremely relevant’ combined (n=308)** | | **n** | **%*** |
| --- | --- | --- | --- |
| Limited access to general hospital to provide liaison services (n=251) | 1. Not relevant | 102 | 40.6 |
|  | 2. Slightly | 55 | 21.9 |
|  | 3. Moderately | 39 | 15.5 |
|  | 4. Very | 29 | 11.6 |
|  | 5. Extremely relevant | 26 | 10.4 |
| Difficulty providing a good service for people who have harmed themselves (n=251) | 1. Not relevant | 84 | 33.5 |
|  | 2. Slightly | 68 | 27.1 |
|  | 3. Moderately | 51 | 20.3 |
|  | 4. Very | 26 | 10.4 |
|  | 5. Extremely relevant | 22 | 8.8 |
| Increased demand for crisis assessments (n=253) | 1. Not relevant | 90 | 35.6 |
|  | 2. Slightly | 62 | 24.5 |
|  | 3. Moderately | 55 | 21.7 |
|  | 4. Very | 26 | 10.3 |
|  | 5. Extremely relevant | 20 | 7.9 |
| Increased practical difficulties in organising Mental Health Act assessments (n=250) | 1. Not relevant | 77 | 30.8 |
|  | 2. Slightly | 64 | 25.6 |
|  | 3. Moderately | 66 | 26.4 |
|  | 4. Very | 22 | 8.8 |
|  | 5. Extremely relevant | 21 | 8.4 |
| *Percentages are of those who answered. The highest number of missing responses for a challenge was 59 (19.2% of participants working in crisis assessment services, n=308). This was for three separate challenges where n=249. | | | |

# Table 6: Current work challenges specific to participants working in community teams and psychological treatment services, in order of % rated ‘Very’ and ‘Extremely relevant’ combined (n=1,268)

|  | | **n** | **%*** |
| --- | --- | --- | --- |
| Not being able to depend on other services that are normally available in the community (primary care, social care, voluntary sector services) (n=1,065) | 1. Not relevant | 93 | 8.7 |
|  | 2. Slightly | 189 | 17.8 |
|  | 3. Moderately | 268 | 25.2 |
|  | 4. Very | 251 | 23.6 |
|  | 5. Extremely relevant | 264 | 24.8 |
| Difficulty providing sufficient support with reduced numbers of face to face contacts (n=1,069) | 1. Not relevant | 134 | 12.5 |
|  | 2. Slightly | 207 | 19.4 |
|  | 3. Moderately | 248 | 23.2 |
|  | 4. Very | 240 | 22.5 |
|  | 5. Extremely relevant | 240 | 22.5 |
| Technological difficulties with remote appointments (n=1,068) | 1. Not relevant | 95 | 8.9 |
|  | 2. Slightly | 254 | 23.8 |
|  | 3. Moderately | 289 | 27.1 |
|  | 4. Very | 211 | 19.8 |
|  | 5. Extremely relevant | 219 | 20.5 |
| Difficulties engaging clients in remote appointments (n=1,065) | 1. Not relevant | 103 | 9.7 |
|  | 2. Slightly | 254 | 23.9 |
|  | 3. Moderately | 305 | 28.6 |
|  | 4. Very | 207 | 19.4 |
|  | 5. Extremely relevant | 196 | 18.4 |
| Difficulty assessing clients by phone or video call (n=1,065) | 1. Not relevant | 113 | 10.6 |
|  | 2. Slightly | 266 | 25.0 |
|  | 3. Moderately | 285 | 26.8 |
|  | 4. Very | 198 | 18.6 |
|  | 5. Extremely relevant | 203 | 19.1 |
| Increased pressure because of our clients’ practical and social problems, such as access to food and money (n=1,070) | 1. Not relevant | 131 | 12.2 |
|  | 2. Slightly | 276 | 25.8 |
|  | 3. Moderately | 274 | 25.6 |
|  | 4. Very | 227 | 21.2 |
|  | 5. Extremely relevant | 162 | 15.1 |
| Increased pressure due to clients deteriorating (n=1,067) | 1. Not relevant | 116 | 10.9 |
|  | 2. Slightly | 284 | 26.6 |
|  | 3. Moderately | 352 | 33.0 |
|  | 4. Very | 181 | 17.0 |
|  | 5. Extremely relevant | 134 | 12.6 |
| Challenges supporting clients in residential settings (e.g. due to infection control challenges) (n=1,060) | 1. Not relevant | 471 | 44.4 |
|  | 2. Slightly | 133 | 12.6 |
|  | 3. Moderately | 160 | 15.1 |
|  | 4. Very | 136 | 12.8 |
|  | 5. Extremely relevant | 160 | 15.1 |
| Less access than usual to crisis care outside hospital (n=1,063) | 1. Not relevant | 331 | 31.1 |
|  | 2. Slightly | 253 | 23.8 |
|  | 3. Moderately | 190 | 17.9 |
|  | 4. Very | 151 | 14.2 |
|  | 5. Extremely relevant | 138 | 13.0 |
| Our reluctance to admit clients to psychiatric hospital because of COVID-19 risk (n=1,061) | 1. Not relevant | 474 | 44.7 |
|  | 2. Slightly | 174 | 16.4 |
|  | 3. Moderately | 153 | 14.4 |
|  | 4. Very | 119 | 11.2 |
|  | 5. Extremely relevant | 141 | 13.3 |

| **Current work challenges specific to participants working in community teams and psychological treatment services, in order of % rated ‘Very’ and ‘Extremely relevant’ combined (n=1,268)** | | **n** | **%*** |
| --- | --- | --- | --- |
| Greater difficulty than usual in arranging hospital admission (n=1,067) | 1. Not relevant | 526 | 49.3 |
|  | 2. Slightly | 161 | 15.1 |
|  | 3. Moderately | 164 | 15.4 |
|  | 4. Very | 106 | 9.9 |
|  | 5. Extremely relevant | 110 | 10.3 |
| Increased practical difficulties in organising Mental Health Act assessments (n=1,063) | 1. Not relevant | 577 | 54.3 |
|  | 2. Slightly | 177 | 16.7 |
|  | 3. Moderately | 138 | 13.0 |
|  | 4. Very | 90 | 8.5 |
|  | 5. Extremely relevant | 81 | 7.6 |
| Problems ensuring clients have medication (n=1,065) | 1. Not relevant | 401 | 37.7 |
|  | 2. Slightly | 304 | 28.5 |
|  | 3. Moderately | 190 | 17.8 |
|  | 4. Very | 94 | 8.8 |
|  | 5. Extremely relevant | 76 | 7.1 |
| Problems ensuring safe continuation of medication that requires administration or monitoring in person e.g. depots, clozapine, lithium (n=1,060) | 1. Not relevant | 568 | 53.6 |
|  | 2. Slightly | 202 | 19.1 |
|  | 3. Moderately | 148 | 14.0 |
|  | 4. Very | 57 | 5.4 |
|  | 5. Extremely relevant | 85 | 8.0 |
| Increased demand due to large numbers of referrals (n=1,069) | 1. Not relevant | 518 | 48.5 |
|  | 2. Slightly | 239 | 22.4 |
|  | 3. Moderately | 182 | 17.0 |
|  | 4. Very | 62 | 5.8 |
|  | 5. Extremely relevant | 68 | 6.4 |
| Increased work to engage and support homeless people in the area (n=1,059) | 1. Not relevant | 747 | 70.5 |
|  | 2. Slightly | 115 | 10.9 |
|  | 3. Moderately | 83 | 7.8 |
|  | 4. Very | 52 | 4.9 |
|  | 5. Extremely relevant | 62 | 5.9 |
| *Percentages are of those who answered. The highest number of missing responses for a challenge was 208 (16.4% of participants working in community teams and psychological treatment services, n=1,268). This was for challenges: 'Challenges supporting clients in residential settings (e.g. due to infection control challenges)' and 'Problems ensuring safe continuation of medication that requires administration or monitoring in person e.g. depots, clozapine, lithium' where n=1,060. | | | |

# Table 7: What are the main challenges for infection control for participants working in inpatient and residential services? (Open-ended question)

Summary

This table presents themes from a content analysis of all responses to open-ended questions relevant to infection control by staff working in inpatient settings, crisis houses and residential services. The predominant areas of challenge for infection control described by participants were the lack of clarity around guidance, impediments to following the guidance and the conflict between infection control and providing good quality care and managing the ward environment. Conflicting information in relation to guidance, either due to its evolving nature or inconsistency between sources (for example between Public Health England and local guidance) was reported as an impediment to clear infection control processes. Some guidance was seen as not tailored to mental health settings, where infection control is not usually a prominent consideration, and some reported that clear management regarding guidance to be followed was not available. In relation to patients and care received, impediments were that inpatients or residents who were distressed, very unwell and or cognitively impaired did not always understand or succeed in adhering to infection control measures. Face masks were described as presenting obstacles to clear communication and establishing relationships with patients. Marked challenge for infection control were also raised in relation to emergency situations, such as restraints, where there may not be time to don PPE or where PPE may present a risk in itself. In these instances, it is also impossible for staff to social distance and there is an increased risk of aerosol generation (e.g. through spitting), resulting in risks to both staff and patients. The equipment and facilities available in mental health services were identified as challenges for infection control. Some staff reported limited to access to PPE, and a frequent problem was layout of wards not allowing for social distancing. Finally, participants described additional challenges to adhering to guidelines, including variations in how able staff seemed to stick closely to guidance, and challenges of managing movement off and on the wards due to patient turnover and staff moving around the hospital.

| **Theme** | **Frequency** | **Theme description** | **Indicative quotes** |
| --- | --- | --- | --- |
| Conflicts between infection control and providing good quality care | 98 | A significant challenge was wearing PPE when working with patients or residents, reported to impact significantly on non-verbal-communication and therapeutic relationships. Staff also expressed their concerns for increased mental health difficulties, in particular the impact of wearing PPE on patient anxiety or paranoia and the impact of social distancing on patient psychological wellbeing. Participants also raised ethical concerns over secluding patients who are not following isolation protocols, and that medication use may have increased in this environment.  Working with individuals going through mental health relapse, working with older adults with cognitive impairments or dementia and working with forensic patients were said to be particular areas of challenges. One participant expressed that the measures in place for infection control are impacting “the quality of service” delivered. Another participant expressed concern over patients being discharged prematurely as a result of the pandemic, sharing “we were not able to do the best for our service users”. | *“Wearing a mask on a mental health ward has a lot of other challenges and potential dangers linked to it. For patients coming on to the ward who are scared and confused already it adds to this making aggression more likely and probably resulting in more use of medications which are bad for people's health as well. it is really hard to build rapport with a mask in place which impacts on people's recovery and engagement with staff putting their mental health at risk which could also have life threatening impacts.”* |
| People who cannot or do not readily follow guidance | 89 | Participants frequently described difficulties related to patients’ or residents’ awareness, understanding, adherence to or remembering guidance, for example standing too close to staff and other patients or not consenting to testing/ isolation. Some suggested there was an increased risk of assault or aggression resulting from attempts to enforce guidance. People with dementia, cognitive impairments or brain injuries, adolescents, and people with substance misuse problems were identified as especially likely to have difficulty understanding and following guidance. | *“in mental health patients do not always understand or comply. There is an increased risk of assault and aggression”* |
| Guidance that conflicts or changes | 76 | Participants frequently described problems resulting from guidance conflicting, changing too rapidly, or being unclear. repeatedly expressed concern around guidance. In some instances, this is a result of confusion around the guidance changing frequently (e.g. around using face masks). In others, this was due to guidance either conflicting earlier guidance, not being applicable to the setting or being issued with the resources being available to comply with them e.g. wearing uniforms). Participants attributed some of the confusion to “information overload”, too much information/variations of information from different sources. Other participants felt that the guidance was not led by science, e.g. “I feel that the guidance on use of masks has been driven by their availability rather than need”. | *“The rules change too quickly. Until last week, we were being asked to take home and wash protective goggles, for example. This week, policy has changed to single use only.”* |

| **Theme** | **Frequency** | **Theme description** | **Indicative quotes** |
| --- | --- | --- | --- |
| Ward layout that doesn’t allow physical distancing | 72 | Many participants raised challenges related to ward layout and the physical environment, for example with narrow corridors and small communal spaces. Some described social distancing in ward environments as “impossible”. This was especially in cases where patients share rooms or in a secure hospital. Concerns were also raised in regard to having both Covid-19 positive and negative patients on the same ward. Some of the guidance issued was felt not to fit with these environments. | *“The ward environment does not practically allow it. This includes communal areas and ward offices.”* |
| Lack of space in offices for physical distances | 70 | Participants also described difficulties in achieving social distancing in staff areas with colleagues. In many cases, this was a result of small office spaces which are not wide enough. Where participants were working on a reduced rota, the concerns around the sustainability of working in such small numbers was raised. Multiple participants also highlighted that areas, such as the nursing office, are too small for social distancing to be possible. | *“It is impossible to social distance in this environment because the corridors/rooms are too small.”* |
| PPE availability | 66 | Many participants described either a delay in accessing PPE or an insufficient supply. Participants also raised access to PPE as an issue, either it being stockpiled by other staff or only one member of staff having a key to the storage cupboard. This was also a challenge in relation to adhering to guidance, “Changing PPE often, we don't have enough/get told off if we use too much as they are using cameras/stock-checks to ensure what is being used.” Others said that they had not received training in its safe use, and that in general mental health staff tend have very limited training in infection control.  The availability and practicality of uniforms and scrubs was raised by participants, particularly in relation to delays in accessing these or in how they fit staff. Some of the PPE available was also said to be inadequate, for example wearing food gloves that tore easily or too small aprons. Other participants shared challenges of wearing own clothes and needing to wash these frequently at 60 degrees. | *“PPE is the main issue - guidance is confused, changes daily and is predicated on the assumption that we have adequate stocks to follow it.”*  *“We either haven't had it or not enough of it to use correctly (sessional use). We have needed to reuse masks which has given the impression of undermining the purpose of PPE.”* |
| Facilities around PPE e.g. disposal and removal of PPE. | 43 | Some participants also described lack of suitable facilities for changing into PPE, including appropriate spaces for changing and disposal bins, and sufficient availability of sinks or hand sanitiser. | *“Mental health wards not set up the same as physical wards for infection control/PPE, e.g. handwashing facilities, facilities for changing PPE.”* |

| **Theme** | **Frequency** | **Theme description** | **Indicative quotes** |
| --- | --- | --- | --- |
| Staff adhering to guidance | 38 | Some participants wrote about situations in which colleagues were not adhering to infection control regulations. Sometimes this was felt to relate to lack of understanding or motivation to follow it. It was also noted that participants expressed challenges for infection control relating to adhering to guidance. In some instances, this related to a lack of staff adherence or understanding of the guidance. Other impediments were that that donning PPE was found cumbersome and time-consuming. Furthermore, staffing levels, whether understaffing or overstaffing were identified by participants as challenges to adhering to infection control guidance. | *“Some colleagues are placing themselves and others at risk by not observing this on a regular basis, thereby impacting those of us who do adhere to guidelines and policies.”* |
| Lack of guidance about some situations | 24 | Participants expressed concern and confusion around the lack of guidance in certain situations, particularly around testing and how the guidance can apply to mental health settings. For example, participants felt that the rules regarding isolation and testing on wards, including for situations when patients refused to be tested. Processes for working in forensic units, or with people who are very distressed or disturbed were reported to require more clarity. | *“No discussion in national media about how PHE guidance can be followed with patients who are experiencing extreme psychiatric distress. Makes a lot of assumptions about people staying two metres apart and not spitting etc.”* |
| Difficulty controlling movement around hospitals | 22 | Some participants expressed concern regarding infection control and movement around hospital units. This was in relation both to staff and equipment moving between wards and patients exiting and entering the ward. Particular challenges for staff moving between wards included time taken to don PPE and the increased exposure and chance of infecting others. Relatively high turnover of patients on wards and some movements between then resulted in risks involving patients spreading this. Community leave was often severely restricted, raising concerns about patients’ rights and the therapeutic environment. | *“Cross contamination from other wards has been a worry for many staff.”*  *“Difficult due to staff footfall and high turnover of patients.”* |

# Table 8: What are the main challenges for infection control for participants working in community settings? (Open-ended question)

Summary

Again, we present here a content analysis of all responses to open-ended questions relevant to infection control, in this table from staff working in a range of community and outpatient settings. The layout and crowded nature of office buildings was the most frequently cited obstacle to social distancing. Other problems related to lack of relevant guidance, guidance that appeared impractical or inappropriate, and shifting or unclear guidance. A few participants reported feeling under pressure from managers in ways that reduced safety, for example that they felt under some pressure to travel to work in the office when this seemed unnecessary. A conflict was expressed by many between following guidance and providing care of high quality. Use of PPE in clinical meetings was felt to impede communication and service user engagement, due to the use of PPE as well as having remote interactions, rather than in person. A further concern related to service users who are unable or unwilling to adhere to infection control. Many of the participants stated they experienced difficulties in obtaining PPE, and that there were issues regarding the facilities surrounding PPE and sanitisation. Community mental health settings were seen as given low priority regarding supplies of PPE, while others reported that some of their colleagues seemed not to take social distancing requirements sufficiently seriously.

| **Theme** | **Frequency** | **Theme description** | **Indicative quotes** |
| --- | --- | --- | --- |
| Lack of space in office buildings for physical distancing | 191 | Many participants reported that their workspace does not enable them to socially distance. This is due to reasons such as a lack of space, shared communal areas, open plan offices, narrow corridors and hot desking. | *“Our office is so small that it is impossible to maintain social distance whilst working… there is no space and no way we can always maintain the distance.”* |
| PPE availability | 97 | Many of the participants described difficulties in obtaining PPE, and that they occasionally had to put themselves at a risk of infection as a result. | *“Lack of PPE so staff putting selves in danger to do essential visits”* |

| **Theme** | **Frequency** | **Theme description** | **Indicative quotes** |
| --- | --- | --- | --- |
| Conflicts between infection control and providing care as needed | 72 | A conflict was perceived between PPE use and providing necessary care, especially when home visits were a necessity (for example for assessments under the Mental Health Act). PPE use and maintaining social distancing was seen as particular challenging on home visits. This was balanced against the limitations of engaging service users and conducting assessments remotely. | *“We have basic PPE and local practice guidance however when undertaking MHA assessments, our dynamic risk assessments are vital to respond to patients, ourselves and others. Hard to maintain social distancing at times.”* |
| Facilities around PPE e.g. for donning and doffing, other facilities needed for infection control | 70 | Some participants reported a lack of facilities for putting on and disposing of PPE and for sanitisation in community service settings. This included a lack of access to hand gels and cleaning supplies. | *“Difficulty with PPE e.g. where to dispose of dirty PPE as not all community bases have clinic rooms/clinical waste disposal facilities - do we dispose of used PPE at home after community visits, or drive it to another hospital to dispose of in their clinical waste bins? - this has not been properly thought through for community teams.”* |
| Impractical, unclear, or inappropriate advice and guidance | 54 | A further reported impediment was advice that was felt to be impractical or inappropriate. Some felt that guidance was being issued on infection control settings that was primarily tailored to hospital settings. Others felt they were inappropriately instructed to deliver remote care in situations when this was not practical, or to come to the office when this was unnecessary. | *“I also feel a culture of we’re NHS heroes has developed rather than thinking about - is it absolutely essential to travel into work today?”*  *“It is unsafe in my opinion to insist on all patients being reviewed remotely: many need to be examined (side effects, general physical health, proper mental state examination), and relying on informants is unsafe.”* |
| Communication and engagement | 51 | PPE was described as having a negative impact on rapport, making communication and engagement more difficult. This was balanced against needs to engage face to face with some clients who were unlikely to be engaged by any other means. | *“PPE acts as a barrier not only to infection but also to being able to connect with your client”*  *“Many of my clients are chaotic, and several refuse to answer their phones or read their letters. I regularly have to see people at home”* |
| **Theme** | **Frequency** | **Theme description** | **Indicative quotes** |
| Guidance that conflicts or changes, or is hard to understand and absorb | 46 | Participations reported that the guidance frequently changed, or that they had received conflicting guidance | *“Constantly changing landscape; feels like they are making it up as they go along and most probably in relation to available PPE....”* |
| Lack of guidance about situations in the community | 32 | Some participants reported that they had received a lack of guidance or clear management in relation to some situations they encountered. | *“We have got generally very poor, unclear and inconsistent guidance from our managers which have made much of our work challenging professionally and distressing personally.”* |

# Table 9: Service activity* (responses from individual staff not individual services) (n=2,180)

|  | | **Increased by more than 20%** | | **Increased by between 11 and 20%** | | **Within 10% of usual over past year** | | **Decreased by between 11 and 20%** | | **Decreased by more than 20%** | | **Not sure** | |
| --- | --- | --- | --- | --- | --- | --- | --- | --- | --- | --- | --- | --- | --- |
|  |  | **n** | **%**** | **n** | **%**** | **n** | **%**** | **n** | **%**** | **n** | **%**** | **n** | **%***** |
| Inpatient services (incl. crisis house) (n=644) | What has happened to your monthly admission rate during the period of the pandemic? (n=428) | 26 | 10.2 | 32 | 12.5 | 60 | 23.4 | 55 | 21.5 | 83 | 32.4 | 172 | 40.2 |
|  | What has happened to the rate of compulsory admissions under the Mental Health Act in the setting where you work during the period of the pandemic? (n=413) | 27 | 13.0 | 33 | 15.9 | 88 | 42.3 | 33 | 15.9 | 27 | 13.0 | 205 | 49.6 |
| Crisis assessment services (n=308) | What has happened to the size of your team's caseload currently compared with the team's typical caseload? (n=202) | 14 | 9.5 | 9 | 6.1 | 48 | 32.7 | 26 | 17.7 | 50 | 34.0 | 55 | 27.2 |
|  | What has happened to the rate of referrals to your service? (n=201) | 8 | 5.3 | 13 | 8.6 | 38 | 25.0 | 32 | 21.1 | 61 | 40.1 | 49 | 24.4 |
| Community teams & psychological treatment services (n=1,268) | What has happened to the rate of referrals to your team during the period of the pandemic? (n=999) | 36 | 5.5 | 62 | 9.5 | 168 | 25.7 | 128 | 19.6 | 259 | 39.7 | 346 | 34.6 |
|  | What has happened to the number of client contacts you have each week? (n=997) | 137 | 16.0 | 145 | 16.9 | 251 | 29.2 | 134 | 15.6 | 192 | 22.4 | 138 | 13.8 |
| *A participant may work in more than one service (e.g. an inpatient service and a crisis assessment service). | | | | | | | | | | | | | |
| **Percentages are of those who answered and did not select 'Not sure'. The highest rate of missing response was 35.9%. This was for 'Inpatient services (incl. crisis house) (n=644): What has happened to the rate of compulsory admissions under the Mental Health Act in the setting where you work during the period of the pandemic?', where 231 responses were missing.  ***Percentages are of those who answered, including ‘Not sure’. | | | | | | | | | | | | | |

# Table 10: Staff perspectives on service users’ and carers’ problems by service user group, in order of % rated ‘Very’ and ‘Extremely relevant’ combined for ‘All service user groups’* (n=2,180)

|  | | **Adults of working age (n=1,521)** | | **Older adults (incl. dementia) (n=853)** | | **Drug & alcohol problems (n=456)** | | **Children & adolescents (n=443)** | | **Forensic (n=365)** | | **Perinatal (n=363)** | | **Intellectual disabilities (n=648)** | | **Eating disorders (n=451)** | | **Other (n=111)** | | **All service user groups (n=2,180)** | |
| --- | --- | --- | --- | --- | --- | --- | --- | --- | --- | --- | --- | --- | --- | --- | --- | --- | --- | --- | --- | --- | --- |
|  |  | **n** | **%**** | **n** | **%** | **n** | **%** | **n** | **%** | **n** | **%** | **n** | **%** | **n** | **%** | **n** | **%** | **n** | **%** | **n** | **%** |
| Lack of access to usual support networks of family and friends | 1. Not rel. | 45 | 3.0 | 31 | 3.7 | 18 | 4.0 | 16 | 3.6 | 16 | 4.4 | 8 | 2.2 | 28 | 4.3 | 16 | 3.6 | 8 | 7.2 | 71 | 3.3 |
|  | 2. Slightly | 88 | 5.8 | 55 | 6.5 | 26 | 5.8 | 39 | 8.9 | 26 | 7.2 | 20 | 5.5 | 32 | 5.0 | 32 | 7.1 | 8 | 7.2 | 129 | 5.9 |
|  | 3. Mod. | 259 | 17.1 | 125 | 14.7 | 85 | 18.8 | 82 | 18.6 | 55 | 15.2 | 46 | 12.7 | 110 | 17.1 | 70 | 15.6 | 14 | 12.6 | 362 | 16.7 |
|  | 4. Very | 484 | 32.0 | 248 | 29.2 | 134 | 29.7 | 140 | 31.8 | 108 | 29.9 | 109 | 30.1 | 203 | 31.5 | 123 | 27.5 | 32 | 28.8 | 671 | 30.9 |
|  | 5. Ex. rel. | 637 | 42.1 | 391 | 46.0 | 189 | 41.8 | 163 | 37.1 | 156 | 43.2 | 179 | 49.5 | 272 | 42.2 | 207 | 46.2 | 49 | 44.1 | 938 | 43.2 |
| Loneliness due to or made worse by social distancing, self-isolation and/or shielding | 1. Not rel. | 54 | 3.6 | 33 | 3.9 | 16 | 3.5 | 22 | 5.0 | 22 | 6.0 | 12 | 3.3 | 23 | 3.6 | 19 | 4.2 | 7 | 6.3 | 88 | 4.0 |
|  | 2. Slightly | 103 | 6.8 | 47 | 5.5 | 36 | 7.9 | 57 | 12.9 | 35 | 9.6 | 20 | 5.5 | 54 | 8.3 | 37 | 8.2 | 9 | 8.1 | 177 | 8.1 |
|  | 3. Mod. | 282 | 18.5 | 138 | 16.2 | 75 | 16.5 | 99 | 22.4 | 63 | 17.3 | 56 | 15.4 | 121 | 18.7 | 77 | 17.1 | 20 | 18.0 | 411 | 18.9 |
|  | 4. Very | 443 | 29.1 | 235 | 27.6 | 127 | 27.9 | 118 | 26.6 | 95 | 26.0 | 118 | 32.5 | 175 | 27.0 | 117 | 25.9 | 28 | 25.2 | 606 | 27.8 |
|  | 5. Ex. rel. | 639 | 42.0 | 400 | 46.9 | 202 | 44.3 | 147 | 33.2 | 150 | 41.1 | 157 | 43.3 | 275 | 42.4 | 201 | 44.6 | 47 | 42.3 | 898 | 41.2 |
| Lack of usual work and activities | 1. Not rel. | 79 | 5.2 | 60 | 7.1 | 26 | 5.8 | 20 | 4.6 | 28 | 7.8 | 12 | 3.4 | 40 | 6.2 | 30 | 6.7 | 8 | 7.2 | 131 | 6.1 |
|  | 2. Slightly | 127 | 8.4 | 80 | 9.5 | 40 | 8.9 | 48 | 10.9 | 33 | 9.1 | 29 | 8.1 | 57 | 8.9 | 44 | 9.9 | 7 | 6.3 | 185 | 8.6 |
|  | 3. Mod. | 285 | 18.9 | 153 | 18.1 | 73 | 16.2 | 85 | 19.4 | 65 | 18.0 | 72 | 20.1 | 99 | 15.4 | 67 | 15.1 | 21 | 18.9 | 417 | 19.3 |
|  | 4. Very | 455 | 30.1 | 254 | 30.1 | 148 | 32.9 | 133 | 30.3 | 95 | 26.3 | 122 | 34.1 | 190 | 29.5 | 134 | 30.1 | 32 | 28.8 | 619 | 28.6 |
|  | 5. Ex. rel. | 564 | 37.4 | 298 | 35.3 | 163 | 36.2 | 153 | 34.9 | 140 | 38.8 | 123 | 34.4 | 258 | 40.1 | 170 | 38.2 | 43 | 38.7 | 813 | 37.6 |
| Worries about getting COVID-19 infection | 1. Not rel. | 22 | 1.5 | 16 | 1.9 | 6 | 1.3 | 10 | 2.3 | 4 | 1.1 | 3 | 0.8 | 13 | 2.0 | 7 | 1.6 | 2 | 1.8 | 38 | 1.8 |
|  | 2. Slightly | 173 | 11.4 | 87 | 10.3 | 46 | 10.1 | 61 | 13.9 | 37 | 10.2 | 34 | 9.4 | 74 | 11.4 | 57 | 12.7 | 17 | 15.3 | 252 | 11.6 |
|  | 3. Mod. | 351 | 23.2 | 193 | 22.8 | 105 | 23.1 | 136 | 30.9 | 81 | 22.3 | 86 | 23.7 | 165 | 25.5 | 110 | 24.4 | 24 | 21.6 | 542 | 25.0 |
|  | 4. Very | 464 | 30.7 | 252 | 29.8 | 143 | 31.4 | 125 | 28.4 | 103 | 28.3 | 116 | 32.0 | 189 | 29.2 | 125 | 27.8 | 34 | 30.6 | 628 | 29.0 |
|  | 5. Ex. rel. | 503 | 33.3 | 299 | 35.3 | 155 | 34.1 | 108 | 24.6 | 139 | 38.2 | 124 | 34.2 | 207 | 31.9 | 151 | 33.6 | 34 | 30.6 | 706 | 32.6 |
| Lack of access to usual support from other services (primary care, social care, voluntary sector) | 1. Not rel. | 84 | 5.5 | 58 | 6.8 | 27 | 5.9 | 26 | 5.9 | 35 | 9.6 | 12 | 3.3 | 41 | 6.3 | 28 | 6.2 | 11 | 9.9 | 141 | 6.5 |
|  | 2. Slightly | 156 | 10.3 | 73 | 8.6 | 44 | 9.7 | 48 | 10.9 | 38 | 10.4 | 32 | 8.8 | 65 | 10.1 | 43 | 9.5 | 11 | 9.9 | 208 | 9.6 |
|  | 3. Mod. | 325 | 21.5 | 201 | 23.6 | 107 | 23.5 | 109 | 24.7 | 81 | 22.2 | 87 | 24.0 | 137 | 21.2 | 100 | 22.2 | 21 | 18.9 | 491 | 22.6 |
|  | 4. Very | 445 | 29.4 | 223 | 26.2 | 120 | 26.4 | 127 | 28.8 | 98 | 26.9 | 110 | 30.3 | 166 | 25.7 | 122 | 27.1 | 31 | 27.9 | 603 | 27.8 |
|  | 5. Ex. rel. | 505 | 33.3 | 296 | 34.8 | 157 | 34.5 | 131 | 29.7 | 113 | 31.0 | 122 | 33.6 | 238 | 36.8 | 158 | 35.0 | 37 | 33.3 | 730 | 33.6 |
| Worries about family getting COVID-19 infection | 1. Not rel. | 40 | 2.6 | 29 | 3.4 | 13 | 2.9 | 11 | 2.5 | 9 | 2.5 | 4 | 1.1 | 20 | 3.1 | 13 | 2.9 | 3 | 2.7 | 60 | 2.8 |
|  | 2. Slightly | 189 | 12.5 | 108 | 12.7 | 56 | 12.3 | 65 | 14.7 | 40 | 11.0 | 31 | 8.6 | 72 | 11.1 | 52 | 11.6 | 20 | 18.2 | 274 | 12.6 |
|  | 3. Mod. | 371 | 24.5 | 196 | 23.0 | 115 | 25.2 | 131 | 29.6 | 79 | 21.7 | 100 | 27.6 | 172 | 26.6 | 119 | 26.4 | 27 | 24.6 | 539 | 24.9 |
|  | 4. Very | 412 | 27.2 | 233 | 27.4 | 118 | 25.9 | 121 | 27.3 | 98 | 26.9 | 98 | 27.1 | 170 | 26.3 | 111 | 24.7 | 24 | 21.8 | 587 | 27.1 |
|  | 5. Ex. rel. | 502 | 33.2 | 286 | 33.6 | 154 | 33.8 | 115 | 26.0 | 138 | 37.9 | 129 | 35.6 | 213 | 32.9 | 155 | 34.4 | 36 | 32.7 | 709 | 32.7 |

| **Staff perspectives on service users’ and carers’ problems by patient group, in order of % rated ‘Very’ and ‘Extremely relevant’ combined for ‘All patient groups’* (n=2,180)** | | **Adults of working age (n=1,521)** | | **Older adults (incl. dementia) (n=853)** | | **Drug & alcohol problems (n=456)** | | **Children & adolescents (n=443)** | | **Forensic (n=365)** | | **Perinatal (n=363)** | | **Intellectual disabilities (n=648)** | | **Eating disorders (n=451)** | | **Other (n=111)** | | **All service user groups (n=2,180)** | |
| --- | --- | --- | --- | --- | --- | --- | --- | --- | --- | --- | --- | --- | --- | --- | --- | --- | --- | --- | --- | --- | --- |
|  |  | **n** | **%**** | **n** | **%** | **n** | **%** | **n** | **%** | **n** | **%** | **n** | **%** | **n** | **%** | **n** | **%** | **n** | **%** | **n** | **%** |
| Increased difficulties for families/carers | 1. Not rel. | 122 | 8.1 | 76 | 9.0 | 37 | 8.2 | 28 | 6.4 | 43 | 11.9 | 18 | 5.0 | 49 | 7.6 | 33 | 7.4 | 12 | 10.9 | 178 | 8.2 |
|  | 2. Slightly | 210 | 13.9 | 108 | 12.8 | 70 | 15.5 | 43 | 9.8 | 50 | 13.8 | 53 | 14.7 | 74 | 11.5 | 60 | 13.5 | 19 | 17.3 | 269 | 12.5 |
|  | 3. Mod. | 381 | 25.3 | 191 | 22.6 | 115 | 25.5 | 111 | 25.3 | 84 | 23.2 | 99 | 27.5 | 158 | 24.6 | 113 | 25.3 | 25 | 22.7 | 525 | 24.3 |
|  | 4. Very | 411 | 27.3 | 224 | 26.5 | 118 | 26.2 | 118 | 26.9 | 89 | 24.6 | 104 | 28.9 | 168 | 26.1 | 125 | 28.0 | 29 | 26.4 | 574 | 26.6 |
|  | 5. Ex. rel. | 384 | 25.5 | 247 | 29.2 | 111 | 24.6 | 139 | 31.7 | 96 | 26.5 | 86 | 23.9 | 194 | 30.2 | 115 | 25.8 | 25 | 22.7 | 615 | 28.5 |
| Lack of access to usual support from NHS mental health services | 1. Not rel. | 138 | 9.1 | 80 | 9.4 | 39 | 8.6 | 43 | 9.7 | 57 | 15.6 | 26 | 7.2 | 66 | 10.2 | 40 | 8.9 | 13 | 11.7 | 219 | 10.1 |
|  | 2. Slightly | 193 | 12.7 | 108 | 12.7 | 57 | 12.5 | 63 | 14.3 | 49 | 13.4 | 39 | 10.8 | 90 | 14.0 | 65 | 14.4 | 8 | 7.2 | 283 | 13.0 |
|  | 3. Mod. | 368 | 24.3 | 210 | 24.7 | 107 | 23.5 | 116 | 26.2 | 82 | 22.5 | 101 | 27.9 | 151 | 23.4 | 103 | 22.9 | 33 | 29.7 | 550 | 25.3 |
|  | 4. Very | 370 | 24.4 | 199 | 23.4 | 117 | 25.7 | 109 | 24.7 | 77 | 21.1 | 96 | 26.5 | 147 | 22.8 | 105 | 23.3 | 30 | 27.0 | 519 | 23.9 |
|  | 5. Ex. rel. | 447 | 29.5 | 254 | 29.9 | 135 | 29.7 | 111 | 25.1 | 100 | 27.4 | 100 | 27.6 | 191 | 29.6 | 137 | 30.4 | 27 | 24.3 | 601 | 27.7 |
| Relapse and deterioration in mental health triggered by COVID-19 stresses | 1. Not rel. | 110 | 7.2 | 100 | 11.7 | 33 | 7.2 | 35 | 7.9 | 38 | 10.4 | 15 | 4.1 | 50 | 7.7 | 31 | 6.9 | 8 | 7.2 | 197 | 9.0 |
|  | 2. Slightly | 276 | 18.2 | 137 | 16.1 | 76 | 16.7 | 91 | 20.5 | 70 | 19.2 | 52 | 14.3 | 131 | 20.2 | 85 | 18.9 | 24 | 21.6 | 409 | 18.8 |
|  | 3. Mod. | 396 | 26.0 | 204 | 23.9 | 109 | 23.9 | 125 | 28.2 | 71 | 19.5 | 114 | 31.4 | 161 | 24.9 | 110 | 24.4 | 24 | 21.6 | 564 | 25.9 |
|  | 4. Very | 369 | 24.3 | 197 | 23.1 | 119 | 26.1 | 103 | 23.3 | 80 | 21.9 | 95 | 26.2 | 160 | 24.7 | 113 | 25.1 | 30 | 27.0 | 511 | 23.4 |
|  | 5. Ex. rel. | 370 | 24.3 | 215 | 25.2 | 119 | 26.1 | 89 | 20.1 | 106 | 29.0 | 87 | 24.0 | 146 | 22.5 | 112 | 24.8 | 25 | 22.5 | 499 | 22.9 |
| High personal risk of severe consequences of COVID-19 infection (e.g. due to physical health comorbidities) | 1. Not rel. | 181 | 12.0 | 106 | 12.5 | 45 | 10.0 | 109 | 24.8 | 47 | 13.0 | 39 | 10.9 | 91 | 14.2 | 61 | 13.7 | 14 | 12.6 | 320 | 14.8 |
|  | 2. Slightly | 260 | 17.2 | 120 | 14.2 | 81 | 17.9 | 111 | 25.2 | 48 | 13.2 | 70 | 19.5 | 106 | 16.5 | 78 | 17.5 | 24 | 21.6 | 371 | 17.1 |
|  | 3. Mod. | 370 | 24.5 | 178 | 21.0 | 102 | 22.6 | 87 | 19.8 | 78 | 21.5 | 95 | 26.5 | 147 | 22.9 | 93 | 20.8 | 25 | 22.5 | 489 | 22.6 |
|  | 4. Very | 345 | 22.8 | 200 | 23.6 | 119 | 26.3 | 76 | 17.3 | 87 | 24.0 | 89 | 24.8 | 145 | 22.6 | 118 | 26.4 | 19 | 17.1 | 470 | 21.7 |
|  | 5. Ex. rel. | 355 | 23.5 | 244 | 28.8 | 105 | 23.2 | 57 | 13.0 | 103 | 28.4 | 66 | 18.4 | 154 | 24.0 | 97 | 21.7 | 29 | 26.1 | 518 | 23.9 |
| Increase in reliance on family/family tensions | 1. Not rel. | 186 | 12.3 | 125 | 14.8 | 64 | 14.2 | 39 | 8.8 | 83 | 22.8 | 24 | 6.7 | 81 | 12.6 | 53 | 11.9 | 15 | 13.5 | 292 | 13.5 |
|  | 2. Slightly | 292 | 19.4 | 153 | 18.1 | 72 | 16.0 | 70 | 15.9 | 72 | 19.8 | 66 | 18.4 | 110 | 17.1 | 76 | 17.0 | 29 | 26.1 | 404 | 18.7 |
|  | 3. Mod. | 407 | 27.0 | 219 | 25.9 | 120 | 26.6 | 117 | 26.5 | 74 | 20.3 | 104 | 29.1 | 178 | 27.6 | 116 | 26.0 | 26 | 23.4 | 576 | 26.6 |
|  | 4. Very | 351 | 23.3 | 198 | 23.4 | 103 | 22.8 | 112 | 25.4 | 67 | 18.4 | 99 | 27.7 | 151 | 23.5 | 115 | 25.7 | 23 | 20.7 | 495 | 22.9 |
|  | 5. Ex. rel. | 273 | 18.1 | 151 | 17.9 | 92 | 20.4 | 103 | 23.4 | 68 | 18.7 | 65 | 18.2 | 124 | 19.3 | 87 | 19.5 | 18 | 16.2 | 397 | 18.4 |
| Difficulty understanding or following current government requirements on social distancing, self-isolation and/or shielding | 1. Not rel. | 189 | 12.5 | 108 | 12.7 | 49 | 10.9 | 80 | 18.1 | 34 | 9.4 | 45 | 12.4 | 75 | 11.7 | 62 | 13.9 | 18 | 16.4 | 289 | 13.3 |
|  | 2. Slightly | 301 | 19.9 | 165 | 19.4 | 82 | 18.2 | 108 | 24.4 | 53 | 14.6 | 90 | 24.9 | 128 | 19.9 | 111 | 24.9 | 17 | 15.5 | 438 | 20.2 |
|  | 3. Mod. | 401 | 26.5 | 195 | 23.0 | 132 | 29.3 | 116 | 26.2 | 82 | 22.7 | 110 | 30.4 | 162 | 25.2 | 108 | 24.2 | 39 | 35.5 | 559 | 25.8 |
|  | 4. Very | 272 | 18.0 | 175 | 20.6 | 89 | 19.8 | 75 | 16.9 | 84 | 23.2 | 58 | 16.0 | 126 | 19.6 | 79 | 17.7 | 19 | 17.3 | 385 | 17.8 |
|  | 5. Ex. rel. | 349 | 23.1 | 206 | 24.3 | 98 | 21.8 | 64 | 14.5 | 109 | 30.1 | 59 | 16.3 | 153 | 23.8 | 86 | 19.3 | 17 | 15.5 | 497 | 22.9 |

| **Staff perspectives on service users’ and carers’ problems by patient group, in order of % rated ‘Very’ and ‘Extremely relevant’ combined for ‘All patient groups’* (n=2,180)** | | **Adults of working age (n=1,521)** | | **Older adults (incl. dementia) (n=853)** | | **Drug & alcohol problems (n=456)** | | **Children & adolescents (n=443)** | | **Forensic (n=365)** | | **Perinatal (n=363)** | | **Intellectual disabilities (n=648)** | | **Eating disorders (n=451)** | | **Other (n=111)** | | **All service user groups (n=2,180)** | |
| --- | --- | --- | --- | --- | --- | --- | --- | --- | --- | --- | --- | --- | --- | --- | --- | --- | --- | --- | --- | --- | --- |
|  |  | **n** | **%**** | **n** | **%** | **n** | **%** | **n** | **%** | **n** | **%** | **n** | **%** | **n** | **%** | **n** | **%** | **n** | **%** | **n** | **%** |
| Difficulty engaging with remote appointments by phone or via digital platforms | 1. Not rel. | 172 | 11.4 | 109 | 12.8 | 52 | 11.5 | 31 | 7.0 | 56 | 15.4 | 25 | 6.9 | 74 | 11.5 | 47 | 10.5 | 10 | 9.1 | 236 | 10.9 |
|  | 2. Slightly | 293 | 19.4 | 152 | 17.9 | 77 | 17.1 | 104 | 23.6 | 60 | 16.5 | 84 | 23.1 | 122 | 19.0 | 94 | 21.0 | 26 | 23.6 | 426 | 19.6 |
|  | 3. Mod. | 446 | 29.5 | 241 | 28.4 | 140 | 31.0 | 142 | 32.2 | 108 | 29.7 | 101 | 27.8 | 199 | 31.0 | 127 | 28.4 | 29 | 26.4 | 648 | 29.8 |
|  | 4. Very | 310 | 20.5 | 175 | 20.6 | 85 | 18.9 | 97 | 22.0 | 61 | 16.8 | 76 | 20.9 | 124 | 19.3 | 97 | 21.7 | 20 | 18.2 | 439 | 20.2 |
|  | 5. Ex. rel. | 293 | 19.4 | 172 | 20.3 | 97 | 21.5 | 67 | 15.2 | 79 | 21.7 | 77 | 21.2 | 123 | 19.2 | 83 | 18.5 | 25 | 22.7 | 423 | 19.5 |
| Increased risk from abusive domestic relationships | 1. Not rel. | 326 | 21.6 | 226 | 26.8 | 92 | 20.3 | 93 | 21.1 | 122 | 33.5 | 46 | 12.8 | 161 | 25.0 | 91 | 20.3 | 31 | 27.9 | 555 | 25.6 |
|  | 2. Slightly | 240 | 15.9 | 149 | 17.6 | 61 | 13.4 | 76 | 17.3 | 54 | 14.8 | 52 | 14.4 | 119 | 18.5 | 71 | 15.9 | 17 | 15.3 | 360 | 16.6 |
|  | 3. Mod. | 306 | 20.2 | 166 | 19.6 | 87 | 19.2 | 97 | 22.1 | 48 | 13.2 | 70 | 19.4 | 120 | 18.6 | 87 | 19.4 | 22 | 19.8 | 429 | 19.8 |
|  | 4. Very | 301 | 19.9 | 134 | 15.9 | 96 | 21.2 | 84 | 19.1 | 62 | 17.0 | 90 | 25.0 | 111 | 17.2 | 97 | 21.7 | 18 | 16.2 | 384 | 17.7 |
|  | 5. Ex. rel. | 340 | 22.5 | 170 | 20.1 | 118 | 26.0 | 90 | 20.5 | 78 | 21.4 | 102 | 28.3 | 134 | 20.8 | 102 | 22.8 | 23 | 20.7 | 437 | 20.2 |
| Diminished access to physical health care for problems other than COVID-19 | 1. Not rel. | 203 | 13.4 | 109 | 12.8 | 65 | 14.3 | 86 | 19.5 | 51 | 14.1 | 38 | 10.5 | 103 | 15.9 | 56 | 12.4 | 20 | 18.0 | 324 | 14.9 |
|  | 2. Slightly | 371 | 24.5 | 199 | 23.4 | 98 | 21.6 | 133 | 30.1 | 89 | 24.5 | 84 | 23.2 | 153 | 23.7 | 117 | 25.9 | 25 | 22.5 | 543 | 25.0 |
|  | 3. Mod. | 418 | 27.6 | 226 | 26.6 | 121 | 26.7 | 107 | 24.2 | 87 | 24.0 | 109 | 30.1 | 161 | 24.9 | 118 | 26.2 | 28 | 25.2 | 570 | 26.2 |
|  | 4. Very | 295 | 19.5 | 180 | 21.2 | 92 | 20.3 | 74 | 16.7 | 60 | 16.5 | 80 | 22.1 | 136 | 21.0 | 92 | 20.4 | 17 | 15.3 | 418 | 19.2 |
|  | 5. Ex. rel. | 230 | 15.2 | 136 | 16.0 | 78 | 17.2 | 42 | 9.5 | 76 | 20.9 | 51 | 14.1 | 94 | 14.5 | 68 | 15.1 | 21 | 18.9 | 317 | 14.6 |
| Having to stay at home in poor circumstances, or not having a home to go to | 1. Not rel. | 345 | 22.8 | 240 | 28.3 | 97 | 21.4 | 102 | 23.3 | 123 | 33.8 | 70 | 19.3 | 173 | 26.8 | 111 | 24.8 | 30 | 27.0 | 568 | 26.2 |
|  | 2. Slightly | 265 | 17.5 | 153 | 18.0 | 70 | 15.5 | 83 | 19.0 | 58 | 15.9 | 78 | 21.6 | 119 | 18.4 | 81 | 18.1 | 19 | 17.1 | 401 | 18.5 |
|  | 3. Mod. | 328 | 21.6 | 168 | 19.8 | 89 | 19.7 | 85 | 19.4 | 52 | 14.3 | 72 | 19.9 | 136 | 21.1 | 85 | 19.0 | 24 | 21.6 | 448 | 20.7 |
|  | 4. Very | 297 | 19.6 | 157 | 18.5 | 101 | 22.3 | 91 | 20.8 | 62 | 17.0 | 74 | 20.4 | 117 | 18.1 | 84 | 18.8 | 15 | 13.5 | 394 | 18.2 |
|  | 5. Ex. rel. | 281 | 18.5 | 130 | 15.3 | 96 | 21.2 | 77 | 17.6 | 69 | 19.0 | 68 | 18.8 | 101 | 15.6 | 86 | 19.2 | 23 | 20.7 | 358 | 16.5 |
| Difficulty getting food, money or other basic resources | 1. Not rel. | 192 | 12.7 | 128 | 15.2 | 61 | 13.6 | 77 | 17.6 | 79 | 21.9 | 35 | 9.7 | 107 | 16.7 | 61 | 13.7 | 21 | 18.9 | 349 | 16.1 |
|  | 2. Slightly | 339 | 22.5 | 193 | 22.9 | 84 | 18.7 | 103 | 23.6 | 63 | 17.5 | 79 | 21.8 | 139 | 21.7 | 102 | 22.9 | 24 | 21.6 | 493 | 22.8 |
|  | 3. Mod. | 440 | 29.2 | 232 | 27.5 | 127 | 28.2 | 121 | 27.7 | 89 | 24.7 | 110 | 30.4 | 184 | 28.7 | 121 | 27.2 | 30 | 27.0 | 606 | 28.0 |
|  | 4. Very | 291 | 19.3 | 166 | 19.7 | 92 | 20.4 | 85 | 19.5 | 62 | 17.2 | 83 | 22.9 | 125 | 19.5 | 96 | 21.6 | 20 | 18.0 | 392 | 18.1 |
|  | 5. Ex. rel. | 247 | 16.4 | 125 | 14.8 | 86 | 19.1 | 51 | 11.7 | 68 | 18.8 | 55 | 15.2 | 87 | 13.6 | 65 | 14.6 | 16 | 14.4 | 322 | 14.9 |
| Effects of COVID-19-related trauma | 1. Not rel. | 288 | 19.0 | 186 | 22.0 | 72 | 15.9 | 78 | 17.8 | 63 | 17.4 | 53 | 14.7 | 122 | 18.9 | 81 | 18.1 | 21 | 19.1 | 440 | 20.3 |
|  | 2. Slightly | 409 | 27.0 | 226 | 26.7 | 119 | 26.3 | 139 | 31.7 | 91 | 25.1 | 106 | 29.4 | 172 | 26.7 | 113 | 25.3 | 28 | 25.5 | 595 | 27.5 |
|  | 3. Mod. | 379 | 25.1 | 188 | 22.2 | 120 | 26.5 | 105 | 23.9 | 95 | 26.2 | 88 | 24.4 | 173 | 26.9 | 114 | 25.5 | 27 | 24.6 | 533 | 24.6 |
|  | 4. Very | 251 | 16.6 | 140 | 16.6 | 78 | 17.2 | 66 | 15.0 | 57 | 15.8 | 60 | 16.7 | 94 | 14.6 | 81 | 18.1 | 23 | 20.9 | 342 | 15.8 |
|  | 5. Ex. rel. | 186 | 12.3 | 106 | 12.5 | 64 | 14.1 | 51 | 11.6 | 56 | 15.5 | 53 | 14.7 | 83 | 12.9 | 58 | 13.0 | 11 | 10.0 | 255 | 11.8 |

| **Staff perspectives on service users’ and carers’ problems by patient group, in order of % rated ‘Very’ and ‘Extremely relevant’ combined for ‘All patient groups’* (n=2,180)** | | **Adults of working age (n=1,521)** | | **Older adults (incl. dementia) (n=853)** | | **Drug & alcohol problems (n=456)** | | **Children & adolescents (n=443)** | | **Forensic (n=365)** | | **Perinatal (n=363)** | | **Intellectual disabilities (n=648)** | | **Eating disorders (n=451)** | | **Other (n=111)** | | **All service user groups (n=2,180)** | |
| --- | --- | --- | --- | --- | --- | --- | --- | --- | --- | --- | --- | --- | --- | --- | --- | --- | --- | --- | --- | --- | --- |
|  |  | **n** | **%**** | **n** | **%** | **n** | **%** | **n** | **%** | **n** | **%** | **n** | **%** | **n** | **%** | **n** | **%** | **n** | **%** | **n** | **%** |
| Risk of increased drug and alcohol use or gambling | 1. Not rel. | 342 | 22.6 | 271 | 31.9 | 77 | 17.0 | 163 | 37.2 | 99 | 27.2 | 85 | 23.6 | 183 | 28.4 | 121 | 27.0 | 34 | 30.6 | 672 | 31.0 |
|  | 2. Slightly | 282 | 18.7 | 162 | 19.1 | 73 | 16.1 | 82 | 18.7 | 53 | 14.6 | 71 | 19.7 | 133 | 20.7 | 77 | 17.2 | 26 | 23.4 | 429 | 19.8 |
|  | 3. Mod. | 387 | 25.6 | 176 | 20.7 | 105 | 23.2 | 91 | 20.8 | 75 | 20.6 | 96 | 26.7 | 147 | 22.8 | 99 | 22.1 | 21 | 18.9 | 471 | 21.7 |
|  | 4. Very | 272 | 18.0 | 128 | 15.1 | 97 | 21.4 | 58 | 13.2 | 65 | 17.9 | 55 | 15.3 | 99 | 15.4 | 78 | 17.4 | 12 | 10.8 | 320 | 14.8 |
|  | 5. Ex. rel. | 228 | 15.1 | 113 | 13.3 | 101 | 22.3 | 44 | 10.1 | 72 | 19.8 | 53 | 14.7 | 82 | 12.7 | 74 | 16.5 | 18 | 16.2 | 275 | 12.7 |
| Lack of access to or of equitable provision of physical healthcare for COVID-19 | 1. Not rel. | 400 | 26.4 | 224 | 26.4 | 129 | 28.5 | 148 | 33.4 | 101 | 28.0 | 90 | 24.9 | 188 | 29.1 | 133 | 29.5 | 40 | 36.0 | 605 | 27.8 |
|  | 2. Slightly | 398 | 26.3 | 213 | 25.1 | 93 | 20.5 | 114 | 25.7 | 75 | 20.8 | 101 | 27.9 | 156 | 24.1 | 117 | 25.9 | 22 | 19.8 | 559 | 25.7 |
|  | 3. Mod. | 353 | 23.3 | 203 | 23.9 | 111 | 24.5 | 107 | 24.2 | 87 | 24.1 | 90 | 24.9 | 156 | 24.1 | 97 | 21.5 | 25 | 22.5 | 505 | 23.2 |
|  | 4. Very | 209 | 13.8 | 125 | 14.7 | 70 | 15.5 | 43 | 9.7 | 45 | 12.5 | 48 | 13.3 | 84 | 13.0 | 64 | 14.2 | 11 | 9.9 | 286 | 13.2 |
|  | 5. Ex. rel. | 155 | 10.2 | 85 | 10.0 | 50 | 11.0 | 31 | 7.0 | 53 | 14.7 | 33 | 9.1 | 63 | 9.7 | 40 | 8.9 | 13 | 11.7 | 218 | 10.0 |
| Loss of liberty and rights due to changes in implementation of mental health legislation | 1. Not rel. | 654 | 43.2 | 393 | 46.5 | 187 | 41.3 | 229 | 51.9 | 127 | 35.0 | 173 | 48.1 | 284 | 44.0 | 209 | 46.4 | 54 | 49.1 | 985 | 45.5 |
|  | 2. Slightly | 298 | 19.7 | 173 | 20.5 | 97 | 21.4 | 79 | 17.9 | 74 | 20.4 | 81 | 22.5 | 133 | 20.6 | 91 | 20.2 | 21 | 19.1 | 431 | 19.9 |
|  | 3. Mod. | 261 | 17.3 | 124 | 14.7 | 73 | 16.1 | 61 | 13.8 | 59 | 16.3 | 45 | 12.5 | 103 | 16.0 | 62 | 13.8 | 17 | 15.5 | 337 | 15.6 |
|  | 4. Very | 155 | 10.2 | 89 | 10.5 | 50 | 11.0 | 34 | 7.7 | 43 | 11.9 | 36 | 10.0 | 67 | 10.4 | 49 | 10.9 | 11 | 10.0 | 209 | 9.7 |
|  | 5. Ex. rel. | 145 | 9.6 | 66 | 7.8 | 46 | 10.2 | 38 | 8.6 | 60 | 16.5 | 25 | 6.9 | 58 | 9.0 | 39 | 8.7 | 7 | 6.4 | 203 | 9.4 |
| Lack of access to medication and to processes for administering and monitoring it | 1. Not rel. | 414 | 27.3 | 235 | 27.6 | 112 | 24.6 | 143 | 32.4 | 133 | 36.5 | 79 | 21.9 | 203 | 31.4 | 115 | 25.6 | 39 | 35.1 | 661 | 30.4 |
|  | 2. Slightly | 456 | 30.1 | 242 | 28.4 | 134 | 29.5 | 130 | 29.4 | 87 | 23.9 | 116 | 32.2 | 181 | 28.0 | 136 | 30.3 | 30 | 27.0 | 632 | 29.1 |
|  | 3. Mod. | 362 | 23.9 | 212 | 24.9 | 114 | 25.1 | 95 | 21.5 | 76 | 20.9 | 98 | 27.2 | 143 | 22.1 | 108 | 24.1 | 25 | 22.5 | 496 | 22.8 |
|  | 4. Very | 163 | 10.8 | 94 | 11.0 | 49 | 10.8 | 41 | 9.3 | 37 | 10.2 | 40 | 11.1 | 67 | 10.4 | 49 | 10.9 | 12 | 10.8 | 218 | 10.0 |
|  | 5. Ex. rel. | 120 | 7.9 | 69 | 8.1 | 46 | 10.1 | 33 | 7.5 | 31 | 8.5 | 27 | 7.5 | 52 | 8.1 | 41 | 9.1 | 5 | 4.5 | 165 | 7.6 |
| Problems with police or other authorities because of lack of understanding of /ability to stick to current government requirements | 1. Not rel. | 659 | 43.6 | 393 | 46.2 | 166 | 36.6 | 208 | 47.3 | 135 | 37.0 | 176 | 48.6 | 283 | 43.9 | 204 | 45.4 | 56 | 50.5 | 1,010 | 46.5 |
|  | 2. Slightly | 369 | 24.4 | 188 | 22.1 | 125 | 27.6 | 124 | 28.2 | 89 | 24.4 | 91 | 25.1 | 158 | 24.5 | 108 | 24.1 | 18 | 16.2 | 508 | 23.4 |
|  | 3. Mod. | 266 | 17.6 | 154 | 18.1 | 79 | 17.4 | 54 | 12.3 | 61 | 16.7 | 53 | 14.6 | 108 | 16.8 | 76 | 16.9 | 22 | 19.8 | 357 | 16.5 |
|  | 4. Very | 116 | 7.7 | 63 | 7.4 | 46 | 10.2 | 29 | 6.6 | 47 | 12.9 | 22 | 6.1 | 56 | 8.7 | 36 | 8.0 | 10 | 9.0 | 156 | 7.2 |
|  | 5. Ex. rel. | 103 | 6.8 | 52 | 6.1 | 37 | 8.2 | 25 | 5.7 | 33 | 9.0 | 20 | 5.5 | 39 | 6.1 | 25 | 5.6 | 5 | 4.5 | 139 | 6.4 |
| *Participants may work with more than one patient group (e.g. with adults of working age and forensic). | | | | | | | | | | | | | | | | | | | | | |
| **Percentages are of those who answered. The highest number of missing responses for a challenge was 19 (0.9% of total participants, n=2,180). This was for challenge 'Increased difficulties for families/carers', where n=2,161. | | | | | | | | | | | | | | | | | | | | | |
| ‘Not rel.’=’Not relevant’, ‘Mod.’=Moderately, ‘Ex. rel.’=’Extremely relevant’. | | | | | | | | | | | | | | | | | | | | | |

# Table 11: Groups of service users and carers as of particular concern during the COVID-19 pandemic period (open-ended questions)

Summary

A wide range of responses were obtained as below. Some were especially concerned about impacts on people with particular conditions, people with dementia, learning disabilities and autism, followed by older adults, people with psychosis, perinatal and postnatal women, people with “personality disorder” diagnoses, and those with OCD or anxiety. Socio-economic difficulties and socio-economic vulnerabilities also concern participants; the impact of isolation/loneliness was by far the most prominent subtheme within this theme followed by concerns about people who experience domestic violence and abuse. For some groups, impacts on the service system seem to have made delivering good care particularly challenging: this applied especially to people with whom contact has been lost and to inpatients.

| **Theme** | **Frequency** | **Theme description** | **Indicative Quotes** |
| --- | --- | --- | --- |
| Main theme: Service users and carers with conditions on which there is a specific impact | 1,094 |  |  |
| Subtheme: Concerns about people who are cognitively impaired, including people with dementia and learning disabilities | 334 | Participants raised concerns about people with dementia regarding lack of capacity to understand social distancing and adhere to guidance thus exposing themselves to high risk; some dementia patients seem to interpret measures as punishment. Increased stress, exacerbation of symptoms and increased tension/ aggression reported when patients are asked to follow guidance. The impact of isolation on their mental health was also a focus.  A particular area of concern raised is the shutting down of memory assessment services, respite, day centres and support for those patients e.g. cognitive stimulation therapy, perhaps leading to deterioration of functioning.  Participants also reported being concerned about people with learning disabilities and autism; lack of understanding of the situation, guidance and social distancing was the main concern due to high risk of infection for themselves and others. The importance of routine and structure for those patients was also highlighted. Increased anxiety, restlessness, impulsivity and increase in challenging behaviours were also noted. The difficulties of engaging this group of patients remotely, as well as difficulties with securing and monitoring medication were underscored. The families of people with learning disabilities and challenging behaviour were a further significant concern. | *“I work in a memory service and our client group is predominately adults over the age of 70 with a diagnosis of dementia. Some of our clients live on their own and so due to social distancing and the current lockdown they're not able to have the same level of social support that they had prior to the pandemic. They're no longer able to attend groups such as Cognitive Stimulation Therapy, no longer able to go out to access their local community, and thus are at risk of their functioning deteriorating more quickly because of a lack of stimulation and routine. Also, it is difficult to engage clients with digital technologies because they either do not* *have the equipment…. or need support from someone to be able to use it”*  *“People with dementia - not understanding the risks associated with COVID/social distancing; also, these people being put in to respite and either contracting COVID-19 or losing independence/deteriorating and respite becoming permanent”*  *“Those clients with a Learning Disability who are experiencing difficulty in making adequate contact and understanding loss of freedom of movement due to government restrictions (eg loss of Section 17 leave previously awarded by the MoJ)”*  *“Patients with learning disability and autism who depend wholly on carers/ family for all their needs, loss of routine activities and limited alternate ones to meet their needs resulting in distress/ increase of challenging behaviour”*  *“Carers become unwell/need to socially isolate resulting in very limited support available to a supported living home, resulting in an increase (hopefully transient) in psychotropic medication to manage patient's distress and challenging behaviour. Difficulty arranging changes to medication regime and monitoring them “* |
| **Theme** | **Frequency** | **Theme description** | **Indicative Quotes** |
| Subtheme: Elderly | 185 | Concerns about older adults related to older people’s physical health vulnerability to COVID-19, increased isolation and its impact on mental health which is already a problem for this group. For those living independently, access to shopping and food supplies is an area of concern. | *“Elderly people who are isolated at home and are not seeing their relatives as they might do normally. Loss of their independence and the increased risk of negative consequence on their physical health if they are not supported and take chances (go shopping for example)”*  “*Patients who are non-concordant with medications are also a worry due to decreased monitoring by community mental health teams.”* |
| Subtheme: Concerns about people who have psychosis and may be paranoid/unable to understand situation/relapsing because of it | 154 | Participants reported being concerned about people with psychosis. Main concerns were lack of understanding of the pandemic, guidance and infection control and risk which place them at risk of becoming infected and spreading the virus; the impact of isolation and the potential reluctance to reintegrate to the community after the crisis is also mentioned. Increases in psychotic symptoms and relapse were reported, plus rising anxiety as a result of delusions about contracting the virus and/or harming others. Difficulties reaching patients through technology due to lack of digital skills and fears of using technology to communicate with others was mentioned. | *“I also worry about individuals with psychotic disorders who tend to socially isolate anyway, who may in the longer term be reluctant to reintegrate into the community, particularly if prior supports are reduced”*  *“Clients who have psychosis or other mental health problems that prevent them from understanding or acknowledging social distancing and hygiene guidelines”*  *“Service users with psychosis or underlying paranoia finding the situation particularly difficult, finding increase in psychotic symptoms and anxiety Having to attend wearing PPE seems to increase fear”* |
| Subtheme: People with anxiety disorders, people with OCD or health anxiety on whom COVID-19 may have a particular impact | 94 | Participants expressed concerns about people with anxiety disorders, especially those experiencing OCD and health anxiety. OCD and contamination obsessions seem to be reinforced by the current climate; increased health anxiety was also noted as well as the negative impact of reduced access to coping mechanisms due to self-isolation. One participant also referred to social anxiety as a concern due to loss of social inclusion activities. | *“I am concerned about the people with anxiety issues or health anxieties who I believe are vulnerable to exacerbation of their symptoms”*  *“My clients with OCD are struggling and I am seeing a lot of progress being undone due to sudden halting of graded exposure work and inability to apply usual strategies to challenge compulsive thoughts especially around contamination”*  *“OCD contamination obsessions increasing”* |

| **Theme** | **Frequency** | **Theme description** | **Indicative Quotes** |
| --- | --- | --- | --- |
| Subtheme: People who harm themselves/ may have “personality disorder” diagnosis/ emotional instability/ repeated self-harm | 85 | Participants expressed concerns about people with personality disorders; increased stress and anxiety was reported, impact of lockdown on emotional regulation, compounded by lack of face to face support and crisis services.  Concerns were also raised about people who self-harm and have suicidal ideation; patients were finding it hard to resist the urge to self-harm due to isolation and lack of copying strategies. Major challenges are safety planning, assessing risk and monitoring patients effectively online; another challenge is keeping patients engaged especially when they experience apathy.  Seeking crisis support or help following an incident of self-harm was also a significant problem, with service users advised against or reluctant to attend A & E. | *“Personality disorders - high risk of self- harm/ suicide & crisis admissions unavailable - therapy offered remotely”*  *“I am worried about many of my clients with EUPD who are experiencing increased anxiety / stress and cut off from usual positive coping strategies and support sources so increased risk of self-harm”*  *“I work with people with severe personality disorder and feeling lonely or having an absence of other perspectives can lead clients to take risks as they feel desperate to end the current uncertainty one way or another”*  *“Borderline patients who need crisis care. They are advised not to go to A&E, Samaritans overloaded and therefore long waiting times and chaos in CMHT in first weeks of crisis”* |
| Subtheme: Perinatal women who have much less access currently to support than usual | 78 | Participants were concerned about the impact of isolation on the mental health of perinatal and postnatal women, lack of access to routine care/professionals and partners/families around the time of birth, and delays in accessing mental health services. Some participants also reported increased stress and anxiety in this group and expressed worries about the lack of face to face assessment of the babies. | *“Pregnant women with severe mental health problems who are most at risk due to the shielding and isolation and reduced appointments and contact with professionals. Concerns around lack of or no social supports or previous means of coping with stress.”*  *“Perinatal - both antenatal and postnatal. For example, current protocol is only to allow a partner at the delivery of a baby not during latent phase or postnatally and yet these are often the stages when women experiencing anxiety can have trauma triggered because of lack of staff to mediate experiences and therefore dependent on partners to be present. There has not been an increase in midwifery care to compensate for this restriction. Postnatally - there has been a reduction in health visitors because of re-deployment at a time and circumstances when women and partners may feel most alone as separated from their families.”* |

| **Theme** | **Frequency** | **Theme description** | **Indicative Quotes** |
| --- | --- | --- | --- |
| Subtheme: Drug and alcohol dependent people | 76 | Participants were concerned about this group due to lack of awareness of the virus and a tendency for some not to not to follow the government’s guidance on social distancing. According to professionals, some people with drug and alcohol problems were continuing to socialise, posing a great risk to themselves particularly due to their fragile physical health. Some participants also commented that it is getting harder for those people to resist urges, fears about accidental overdose for those not currently supervised due to lack of resources in pharmacies and patients taking additional risks to access substances. | *“Those at risk of accidental overdose who are no longer on supervised methadone due to lack of resources at pharmacies (I have already heard of deaths).”*  *“Risk of death is greater due to changing drug landscape, crime is increasing as remaining supplies become expensive and safeguarding concerns become more imminent as children are now at home all day. The role is more complex as needs and risks are greater but we lack leadership and adapting has been clumsy. We expect to see more deaths. We also see ambivalence to COVID in this population and they continue to socialise regularly. They deal with bigger risks every day.”* |
| Subtheme: People with eating disorders – disruption of treatment and of access to food and usual | 52 | Patients with eating disorders were seen as a group of concern due to low BMI and physical health vulnerability, risk of relapse, difficulties in monitoring this group and conducting health checks online (weighting and blood tests). Changes in mealtime and social routines, and in food supply also had impacts. | *“Eating Disorders - risks due to GP surgeries declining to complete physical health monitoring (weight/bloods) with very low weight, severely malnourished patients insufficient monitoring in place”*  *“People with Eating disorders not being able to get the food due to supermarket problems - This can lead to exacerbation of the eating disorder”* |

| **Theme** | **Frequency** | **Theme description** | **Indicative Quotes** |
| --- | --- | --- | --- |
| Subtheme: Forensic patients | 36 | Participants reported negative impact of isolation, increased stress, loss of leave and support leading to self-harm and/or aggression towards staff and others, relapse of mental health and increased risk. | *“People/young people suffering from acute mental health conditions who are inpatients in medium-secure forensic settings. Some of them had been moving forward to transition back to the community and on a graduated plan of leave back to their family/community and discharge This has now been put on hold because of lockdown and risk of introducing virus into the unit, and therefore risk to staff and service users alike. Other young people had just started to have s17 MHA leave after a long period of assessment and treatment, so this has put them back in the process and in some cases caused a relapse in mental health/symptoms and risk”*  *“Forensic patients have limited leave anyway but having had five [COVID-19] positive patients on the ward this was taken away altogether. Patients have deteriorated due to being locked inside at all times, living with positive peers and not being able to see family or friends. There is also a lack of awareness around it and so positive patients have continued to come out of their rooms despite being told to self- isolate, they have shared clothes, used communal bathrooms, etc. We have had more violent incidents on staff which have resulted from staff telling patients they are unable to go out on leave”* |
| Main theme: Concerns related to service users’ social circumstances/social characteristics | 651 |  |  |

| **Theme** | **Frequency** | **Theme description** | **Indicative Quotes** |
| --- | --- | --- | --- |
| Subtheme: People who live alone/ are currently socially isolated and lonely/ not receiving usual visits from carers because of crisis | 208 | Participants are particularly concerned about people who live alone and are socially isolated. The impact of isolation on their mental health was underscored as a risk factor particularly for those people who were already isolated before the current crisis. Another area of concern is access to food and essentials for those isolated who are COVID-19 positive. This group are also often digitally isolated with no access/ lack of knowledge about technology and social media. Participants noted that the mental health of these people is deteriorating. | *“Loneliness and social isolation are associated with suicidal ideation and being with no support system (if no family/friends), feeling lonely can increase suicidal thoughts, self-injurious behaviours”*  *“Clients who live alone, already socially isolated Visits from MH workers may be pretty much the only people they see”*  *“Clients who had daily structure and used volunteer organisation are now isolated. Those on their own are struggling to keep any structure and motivation and mental health is deteriorating. Many do not have internet so rely on telephone contact.”* |
| Subtheme: People who are in situations where there is domestic violence/abuse | 164 | Worries about patients experiencing domestic violence and abuse were predominant among participants. Although the majority of participants did not elaborate on this aspect, those who did reported difficulties in monitoring the risk for victims of domestic due to lack of face to face, access, lack of refuge provision and social care support, while one participant reported an increase in mental health difficulties, self-harm and suicidal attempts after episodes of violence. | *“Lack of refuge provision for women looking to flee domestic abuse. No provision available for women with complex needs seeking refuge. Women having to stay in the house with their abuser, unable to access their phones or other sources of help and support”*  *“Women in relationships with DV and families where there are child protection concerns. Current practice is not allowing us to see these families as often as before and it is difficult to monitor the risk for women and their families at this time”* |
| Subtheme: Children who are at risk | 92 | Participants expressed worries for children with safeguarding risks and the risk for those children not meeting other adults (e.g. teachers, counsellors) outside home. | *“Young people with difficult family circumstances - social care is already stretched in the area where I work and it’s extremely difficult to get a young person a social care assessment generally but most social workers are working from home and so accessing social care is a little harder - not impossible but harder”*  *“Young people with difficult family situations or who will be more exposed/vulnerable to domestic violence. Vulnerable young people who cannot access usual support through school”* |
| **Theme** | **Frequency** | **Theme description** | **Indicative Quotes** |
| Subtheme: People living in poverty/ poor housing | 69 | People living in poverty/ poor housing concerned participants; unemployment, access to food and difficulties with accessing benefits were also noted by participants. | *“I work in a specialist parental mental health services working with predominately single mothers, in families where children and parents have complex mental health needs. The impact of years of austerity and a severe reduction in available support services, coupled with universal credit and other benefit issues resulting in poverty was already adversely impacting on the families we work with and their social and economic opportunities, their health and their quality of life. The full impact of COVID-19 and Government restrictions is not yet fully known but it has hit these families really hard. All are struggling with food and utility poverty; the children are home from school and mental health is deteriorating”*  *“Clients with limited access to safe and adequate housing and food provisions”* |
| Subtheme: Carers under increased stress | 63 | Participants were concerned about carers of people with dementia and, less predominantly, carers of people with learning difficulties. Among staff concerns for carers were increased stress and anxiety, burn out, lack of support due to services being closed, emotional and physical exhaustion, increased aggression towards carers, and increased tension and relationship problems, all due to the burden of caring. | *“People with dementia and their carers, who do not currently have access to day care. This provided Carer respite and opportunities for* *Carer to shop without their loved one who cognitively could not manage shopping even without restrictions. Unless physical health conditions co-exist, do not meet COVID vulnerability status for gov support to shop. Majority of local advertised support is internet based and the age group do not use the social media platform for awareness of this”*  *“Very concerned about carers of those suffering with Dementia. Usual support networks have stopped, so stress levels and levels of depression are very high.”* |
| Subtheme: Homeless people | 55 | Participants expressed worries about homeless people and stressed the difficulties in finding accommodation for this group, the risk to their health due to COVID-19, and challenges in accessing food. | *“Homeless or more chaotic clients- harder to track down and support, harder to find them accommodation, harder to put support in place from social care agencies”*  *“Homeless hostels are getting no physical healthcare and no COVID co-hosting and many will die when COVID reaches this community. They are also not getting access to much mental health care.”* |
| **Theme** | **Frequency** | **Theme description** | **Indicative Quotes** |
| Main theme: Concerns related to specific impacts of service disruptions in some groups | 199 |  |  |
| Subtheme: Specific difficulties relating to inpatients e.g. difficulty discharging or precipitate discharge, impacts of increased isolation on ward | 97 | The main difficulties relating to inpatients concerned problems with infection control in the wards, premature discharges due to the closure of units to create space for COVID-19 and the compound effect of the lack of face to face community support following discharge. Delays in discharges and the impact of isolation on those patients who should have been discharged as they do not have access to visitors/ families also noted. Some also referred to lower standards of care being offered due to overreliance on bank staff or the wards not being safely staffed. Some expressed concerns about admissions being prevented. | *“Concerns about the inpatient group as a whole as it is difficult to maintain social distancing between patients and difficult to isolate patients from others if required”*    *“Our in-patients have not been able to go home throughout the lock down, and some have only seen either their mother or father for over a month. One patient has had no visitors, as parents were both tested positive.”*  *“Those discharged prematurely from psychiatric units, as a result of the rapid closure of the units to create additional* *bed space for covid19 physical health management”* |
| Subtheme: People who are difficult to contact in the community currently without usual visiting/outreach/face-to-face appointments | 81 | Patients with mobility issues, patients with long term physical conditions, patients at risk, patients who cannot use technology (due to lack of access/lack of knowledge about how to use it), patients severely isolated who are hard to engage without face-to-face contact all constitute areas of concern. Difficulties in online risk assessment and arising from staff re- deployment were also noted. | *“Chronically I'll patients whom are becoming acutely unwell and not being offered timely in person assessments from community mental health practitioners”*  *“Those in the middle of formulation work who have a partial, yet incomplete understanding of their difficulties. I am concerned this may lead to a belief that the work we have done so far is ineffective, thus risking disengagement. Also, clients in the middle of face-to-face therapy that cannot be continued over the phone - risks of reinforcing core beliefs around abandonment and rejection.”* |
| Subtheme: Problems related to particular medications e.g. clozapine, lithium, depot (where face-to-face contact and monitoring is needed) | 21 | Participants expressed concern for patients who take medications that need daily monitoring and specifically mentioned lithium and clozapine. | *“Depot, lithium and clozapine patients”* |

# Table 12: Are you seeing any mental health problems that seem to arise directly from the current pandemic? (Open-ended question)

Summary

We asked staff whether they felt they were seeing any types of mental health problems, or any symptoms, that seemed to arise directly from the current pandemic. The most frequently discussed, themes, as below, related to anxiety, psychosis, depression, and self-harm. Staff reported such presentations in current service users, people who had been out of contact with services for some time, and people new to services. Some had symptoms with explicitly COVID-related content, while others appeared to have major COVID-19-related triggers to their current mental health problems. Although we did not directly enquire about this, a number of participants reported improvements in mental health in some service users during the pandemic period.

| **Theme** | **Frequency** | **Theme description** | **Indicative Quotes** |
| --- | --- | --- | --- |
| Main Theme: Anxiety | 804 | The most prominent theme noted in responses was that staff were encountering increased anxiety that appeared to be linked to COVID-19, taking the following forms: | *-* |
| Subtheme: Health anxiety and OCD: COVID-19 specific | - | The most prominently discussed anxiety was where anxiety was linked directly to covid-19 with particular focus on OCD and health related presentations. These included new presentations in previously ‘well’ individuals as well as exacerbations or relapses in people with existing health anxiety or OCD. | *“Increase in OCD traits e.g. worrying thoughts of having COVID or spread COVID resulting in increased hand washing or cleaning rituals Fear of leaving the house, increasing social isolation”* |
| Subtheme: General anxiety and stress | - | An increase in generalised anxiety among service users was reported by many staff, not necessarily related to COVID-19. This was often attributed loss of usual supports, contacts and activities, which have served as healthy coping mechanisms. | *“Exacerbated anxiety in many clients, to a severe level Specifically around their loss of identity re past productivity, as they are struggling to redefine and adjust their personal expectations”* |
| Main Theme: Psychosis | 303 | Many participants answered this question in relation to impacts of current circumstances on psychosis. | *-* |

| **Theme** | **Frequency** | **Theme description** | **Indicative Quotes** |
| --- | --- | --- | --- |
| Subtheme: New cases of psychosis | - | There were multiple reports of people presenting to services for the first time with psychotic or manic states, sometimes with COVID-19 related content. | *“Appears to be increased 1st presentation psychosis and manic presentations”*  *“Several health care workers presenting with psychosis apparently directly related to Covid concerns; others with no history of MH problems”* |
| Subtheme: COVID-19 specific psychosis | - | There were also many reports of COVID-19 themes being incorporated in the symptoms of people with existing psychosis, for example presenting with delusions related to covid-19 infection, or paranoia around guidelines and actions being taken (e.g. being locked on the ward) | *“I have seen a number of clients who have incorporated covid19 in paranoid delusional belief systems”* |
| Main Theme: Depression | 276 | Staff reported seeing people with both new onset depression and depressive relapses that they felt likely to be related to the pandemic. Potential causes cited for this included grief, isolation, withdrawal of important services and loss of employment and hopes and plans for the future. This was particularly salient for young people related to fear for the future and the prospect of no hope. | *“Worsening depression due to isolation and uncertainty”*  *“Yes- increased depression due to isolation Hopelessness as a consequence of lack of activity, increasing debt, poverty”* |
| Main Theme: Self-harm | 104 | There were a number of reported increases in self-harm, linked to greater difficulties with emotional regulation in the absence of usual supports and activities. Challenges noted included reduced availability of usual services in A & E to manage consequences of self-harm, or reluctance to attend hospital for these. | *“People are contacting us who have not used self-harm for decades and are finding it is back in their lives which they find frightening”* |
| Main Theme: Improvements related to COVID-19 | 25 | Although this was not directly asked, some participants also described an improvement in the mental health of some service users following the onset of the pandemic. | *“Conversely seeing people feeling better - world is more attuned to people with anxiety and depression”* |

# Table 13: Sources of help in coping with COVID-19 at work by profession, in order of % rated ‘Very’ and ‘Extremely important’ combined for ‘All professions’ (n=2,180)

|  | | **Psych-**  **logist**  **(n=347)** | | **Nurse**  **(n=664)** | | **Occup-**  **ational**  **therapist**  **(n=171)** | | **Other**  **qualified**  **therapist**  **(n=189)** | | **Peer**  **support**  **worker**  **(n=80)** | | **Psychiatrist (n=254)** | | **Social**  **worker**  **(n=97)** | | **Manager,**  **no prof.**  **qual. (n=63)** | | **Other**  **worker**  **(n=307)** | | **All**  **professions**  **(n=2,180)** | |
| --- | --- | --- | --- | --- | --- | --- | --- | --- | --- | --- | --- | --- | --- | --- | --- | --- | --- | --- | --- | --- | --- |
|  |  | **n** | **%*** | **n** | **%** | **n** | **%** | **n** | **%** | **n** | **%** | **n** | **%** | **n** | **%** | **n** | **%** | **n** | **%** | **n** | **%** |
| Guidance from my employer on managing clinical and safety needs due to COVID-19 | 1. Not imp. | 10 | 2.9 | 12 | 1.8 | 1 | 0.6 | 11 | 5.8 | 2 | 2.5 | 6 | 2.4 | 1 | 1.0 | 4 | 6.4 | 17 | 5.5 | 66 | 3.0 |
|  | 2. Slightly | 57 | 16.4 | 55 | 8.3 | 13 | 7.6 | 19 | 10.1 | 9 | 11.3 | 27 | 10.6 | 7 | 7.2 | 9 | 14.3 | 43 | 14.0 | 240 | 11.0 |
|  | 3. Mod. | 80 | 23.1 | 135 | 20.3 | 34 | 19.9 | 32 | 16.9 | 18 | 22.5 | 71 | 28.0 | 24 | 24.7 | 10 | 15.9 | 55 | 17.9 | 459 | 21.1 |
|  | 4. Very | 121 | 34.9 | 212 | 31.9 | 50 | 29.2 | 61 | 32.3 | 25 | 31.3 | 90 | 35.4 | 31 | 32.0 | 18 | 28.6 | 94 | 30.6 | 704 | 32.3 |
|  | 5. Ex. imp. | 79 | 22.8 | 250 | 37.7 | 73 | 42.7 | 66 | 34.9 | 26 | 32.5 | 60 | 23.6 | 34 | 35.1 | 22 | 34.9 | 98 | 31.9 | 711 | 32.6 |
| Support and information from colleagues | 1. Not imp. | 5 | 1.5 | 10 | 1.5 | 1 | 0.6 | 6 | 3.2 | 3 | 3.8 | 3 | 1.2 | 1 | 1.0 | 0 | 0.0 | 8 | 2.6 | 37 | 1.7 |
|  | 2. Slightly | 48 | 13.9 | 46 | 7.0 | 13 | 7.6 | 18 | 9.5 | 8 | 10.0 | 25 | 9.8 | 10 | 10.3 | 6 | 9.5 | 23 | 7.6 | 198 | 9.1 |
|  | 3. Mod. | 91 | 26.3 | 148 | 22.4 | 30 | 17.5 | 40 | 21.2 | 19 | 23.8 | 76 | 29.9 | 27 | 27.8 | 21 | 33.3 | 77 | 25.3 | 532 | 24.5 |
|  | 4. Very | 116 | 33.5 | 216 | 32.7 | 63 | 36.8 | 61 | 32.3 | 23 | 28.8 | 93 | 36.6 | 32 | 33.0 | 23 | 36.5 | 95 | 31.3 | 723 | 33.3 |
|  | 5. Ex. imp. | 86 | 24.9 | 240 | 36.4 | 64 | 37.4 | 64 | 33.9 | 27 | 33.8 | 57 | 22.4 | 27 | 27.8 | 13 | 20.6 | 101 | 33.2 | 682 | 31.4 |
| Support and advice from my manager(s) | 1. Not imp. | 18 | 5.2 | 24 | 3.6 | 0 | 0.0 | 10 | 5.4 | 6 | 7.6 | 18 | 7.1 | 8 | 8.3 | 3 | 4.8 | 15 | 4.9 | 102 | 4.7 |
|  | 2. Slightly | 50 | 14.4 | 51 | 7.7 | 16 | 9.4 | 17 | 9.1 | 12 | 15.2 | 52 | 20.5 | 9 | 9.3 | 9 | 14.5 | 33 | 10.8 | 252 | 11.6 |
|  | 3. Mod. | 83 | 23.9 | 115 | 17.5 | 30 | 17.7 | 31 | 16.6 | 18 | 22.8 | 69 | 27.2 | 19 | 19.6 | 13 | 21.0 | 62 | 20.3 | 441 | 20.3 |
|  | 4. Very | 104 | 30.0 | 218 | 33.1 | 58 | 34.1 | 60 | 32.1 | 19 | 24.1 | 68 | 26.8 | 28 | 28.9 | 14 | 22.6 | 89 | 29.1 | 659 | 30.4 |
|  | 5. Ex. imp. | 92 | 26.5 | 251 | 38.1 | 66 | 38.8 | 69 | 36.9 | 24 | 30.4 | 47 | 18.5 | 33 | 34.0 | 23 | 37.1 | 107 | 35.0 | 715 | 33 |
| Adoption of new digital ways of working | 1. Not imp. | 13 | 3.8 | 45 | 6.8 | 6 | 3.6 | 6 | 3.2 | 6 | 7.5 | 8 | 3.2 | 6 | 6.3 | 1 | 1.6 | 26 | 8.5 | 119 | 5.5 |
|  | 2. Slightly | 34 | 9.9 | 80 | 12.1 | 23 | 13.8 | 18 | 9.6 | 10 | 12.5 | 21 | 8.3 | 10 | 10.4 | 5 | 8.2 | 33 | 10.8 | 234 | 10.8 |
|  | 3. Mod. | 59 | 17.1 | 167 | 25.3 | 46 | 27.5 | 27 | 14.4 | 22 | 27.5 | 66 | 26.0 | 20 | 20.8 | 17 | 27.9 | 66 | 21.6 | 490 | 22.6 |
|  | 4. Very | 112 | 32.5 | 164 | 24.8 | 42 | 25.2 | 50 | 26.6 | 21 | 26.3 | 74 | 29.1 | 28 | 29.2 | 15 | 24.6 | 86 | 28.2 | 595 | 27.5 |
|  | 5. Ex. imp. | 127 | 36.8 | 205 | 31.0 | 50 | 29.9 | 87 | 46.3 | 21 | 26.3 | 85 | 33.5 | 32 | 33.3 | 23 | 37.7 | 94 | 30.8 | 727 | 33.6 |
| Resilience and resourcefulness in adversity among service users and carers | 1. Not imp. | 5 | 1.4 | 18 | 2.7 | 4 | 2.3 | 6 | 3.2 | 6 | 7.5 | 14 | 5.5 | 5 | 5.2 | 3 | 4.8 | 18 | 5.9 | 80 | 3.7 |
|  | 2. Slightly | 42 | 12.1 | 67 | 10.1 | 19 | 11.1 | 17 | 9.0 | 7 | 8.8 | 42 | 16.5 | 10 | 10.3 | 7 | 11.1 | 31 | 10.1 | 242 | 11.1 |
|  | 3. Mod. | 89 | 25.7 | 174 | 26.2 | 40 | 23.4 | 40 | 21.2 | 21 | 26.3 | 78 | 30.7 | 16 | 16.5 | 14 | 22.2 | 76 | 24.8 | 549 | 25.2 |
|  | 4. Very | 112 | 32.3 | 185 | 27.9 | 49 | 28.7 | 57 | 30.2 | 23 | 28.8 | 67 | 26.4 | 24 | 24.7 | 19 | 30.2 | 84 | 27.4 | 623 | 28.6 |
|  | 5. Ex. imp. | 99 | 28.5 | 220 | 33.1 | 59 | 34.5 | 69 | 36.5 | 23 | 28.8 | 53 | 20.9 | 42 | 43.3 | 20 | 31.8 | 98 | 31.9 | 686 | 31.5 |

| **Sources of help by profession, in order of % rated ‘Very’ and ‘Extremely important’ combined for ‘All professions’ (n=2,180)** | | **Psych-**  **logist**  **(n=347)** | | **Nurse**  **(n=664)** | | **Occup-**  **ational**  **therapist**  **(n=171)** | | **Other**  **qualified**  **therapist**  **(n=189)** | | **Peer**  **support**  **worker**  **(n=80)** | | **Psychiatrist**  **(n=254)** | | **Social**  **worker**  **(n=97)** | | **Manager,**  **no prof.**  **qual. (n=63)** | | **Other**  **worker**  **(n=307)** | | **All**  **professions**  **(n=2,180)** | | |
| --- | --- | --- | --- | --- | --- | --- | --- | --- | --- | --- | --- | --- | --- | --- | --- | --- | --- | --- | --- | --- | --- | --- |
|  |  | **n** | **%*** | **n** | **%** | **n** | **%** | **n** | **%** | **n** | **%** | **n** | **%** | **n** | **%** | **n** | **%** | **n** | **%** | **n** | **%** |  |
| Guidance disseminated by the NHS or professional bodies | 1. Not imp. | 7 | 2.0 | 8 | 1.2 | 1 | 0.6 | 5 | 2.7 | 5 | 6.3 | 3 | 1.2 | 3 | 3.1 | 1 | 1.6 | 10 | 3.3 | 43 | 2.0 |  |
|  | 2. Slightly | 49 | 14.2 | 78 | 11.8 | 15 | 8.8 | 22 | 11.7 | 10 | 12.5 | 36 | 14.2 | 10 | 10.4 | 6 | 9.5 | 45 | 14.9 | 273 | 12.6 |  |
|  | 3. Mod. | 83 | 24.0 | 149 | 22.6 | 56 | 32.8 | 41 | 21.8 | 22 | 27.5 | 88 | 34.8 | 27 | 28.1 | 22 | 34.9 | 85 | 28.1 | 574 | 26.5 |  |
|  | 4. Very | 126 | 36.4 | 213 | 32.3 | 39 | 22.8 | 66 | 35.1 | 19 | 23.8 | 78 | 30.8 | 32 | 33.3 | 19 | 30.2 | 82 | 27.1 | 677 | 31.2 |  |
|  | 5. Ex. imp. | 81 | 23.4 | 211 | 32.0 | 60 | 35.1 | 54 | 28.7 | 24 | 30.0 | 48 | 19.0 | 24 | 25.0 | 15 | 23.8 | 81 | 26.7 | 600 | 27.7 |  |
| Being aware of public support for key workers | 1. Not imp. | 43 | 12.4 | 45 | 6.8 | 5 | 2.9 | 29 | 15.6 | 3 | 3.8 | 24 | 9.5 | 14 | 14.4 | 5 | 7.9 | 35 | 11.6 | 204 | 9.4 |  |
|  | 2. Slightly | 97 | 28.0 | 117 | 17.8 | 38 | 22.2 | 43 | 23.1 | 9 | 11.4 | 80 | 31.6 | 14 | 14.4 | 14 | 22.2 | 55 | 18.2 | 467 | 21.6 |  |
|  | 3. Mod. | 101 | 29.2 | 136 | 20.6 | 44 | 25.7 | 40 | 21.5 | 21 | 26.6 | 74 | 29.3 | 29 | 29.9 | 16 | 25.4 | 68 | 22.4 | 531 | 24.5 |  |
|  | 4. Very | 66 | 19.1 | 170 | 25.8 | 41 | 24.0 | 36 | 19.4 | 23 | 29.1 | 51 | 20.2 | 16 | 16.5 | 12 | 19.1 | 65 | 21.5 | 482 | 22.3 |  |
|  | 5. Ex. imp. | 39 | 11.3 | 191 | 29.0 | 43 | 25.2 | 38 | 20.4 | 23 | 29.1 | 24 | 9.5 | 24 | 24.7 | 16 | 25.4 | 80 | 26.4 | 481 | 22.2 |  |
| Staff well-being initiatives set up during COVID-19 in my workplace | 1. Not imp. | 62 | 18.0 | 69 | 10.5 | 11 | 6.5 | 32 | 17.3 | 10 | 12.5 | 57 | 22.7 | 15 | 15.5 | 6 | 9.5 | 46 | 15.2 | 309 | 14.3 |  |
|  | 2. Slightly | 106 | 30.8 | 138 | 20.9 | 43 | 25.4 | 41 | 22.2 | 7 | 8.8 | 72 | 28.7 | 24 | 24.7 | 12 | 19.1 | 58 | 19.1 | 501 | 23.2 |  |
|  | 3. Mod. | 73 | 21.2 | 139 | 21.1 | 43 | 25.4 | 36 | 19.5 | 27 | 33.8 | 76 | 30.3 | 18 | 18.6 | 12 | 19.1 | 67 | 22.1 | 495 | 22.9 |  |
|  | 4. Very | 59 | 17.2 | 147 | 22.3 | 33 | 19.5 | 33 | 17.8 | 15 | 18.8 | 27 | 10.8 | 20 | 20.6 | 22 | 34.9 | 64 | 21.1 | 420 | 19.4 |  |
|  | 5. Ex. imp. | 44 | 12.8 | 167 | 25.3 | 39 | 23.1 | 43 | 23.2 | 21 | 26.3 | 19 | 7.6 | 20 | 20.6 | 11 | 17.5 | 68 | 22.4 | 435 | 20.1 |  |
| National initiatives to support service users and carers, such as helplines and online peer support | 1. Not imp. | 69 | 20.1 | 55 | 8.4 | 13 | 7.8 | 31 | 16.7 | 7 | 8.9 | 57 | 22.8 | 14 | 14.4 | 10 | 16.1 | 44 | 14.4 | 302 | 14.0 |  |
|  | 2. Slightly | 94 | 27.4 | 149 | 22.6 | 36 | 21.6 | 37 | 19.9 | 17 | 21.5 | 93 | 37.2 | 18 | 18.6 | 16 | 25.8 | 60 | 19.6 | 522 | 24.2 |  |
|  | 3. Mod. | 84 | 24.5 | 147 | 22.3 | 44 | 26.4 | 43 | 23.1 | 15 | 19.0 | 61 | 24.4 | 24 | 24.7 | 18 | 29.0 | 70 | 22.9 | 508 | 23.6 |  |
|  | 4. Very | 61 | 17.8 | 147 | 22.3 | 40 | 24.0 | 36 | 19.4 | 19 | 24.1 | 23 | 9.2 | 20 | 20.6 | 8 | 12.9 | 61 | 19.9 | 415 | 19.3 |  |
|  | 5. Ex. imp. | 35 | 10.2 | 161 | 24.4 | 34 | 20.4 | 39 | 21.0 | 21 | 26.6 | 16 | 6.4 | 21 | 21.7 | 10 | 16.1 | 71 | 23.2 | 408 | 18.9 |  |
| New initiatives in NHS mental health services | 1. Not imp. | 50 | 14.5 | 54 | 8.2 | 7 | 4.2 | 30 | 16.0 | 9 | 11.5 | 34 | 13.5 | 18 | 18.6 | 8 | 12.9 | 49 | 16.1 | 260 | 12.1 |  |
|  | 2. Slightly | 103 | 29.9 | 129 | 19.7 | 45 | 26.8 | 39 | 20.7 | 20 | 25.6 | 64 | 25.4 | 22 | 22.7 | 7 | 11.3 | 52 | 17.1 | 481 | 22.3 |  |
|  | 3. Mod. | 96 | 27.8 | 173 | 26.4 | 51 | 30.4 | 42 | 22.3 | 12 | 15.4 | 93 | 36.9 | 22 | 22.7 | 24 | 38.7 | 87 | 28.6 | 602 | 27.9 |  |
|  | 4. Very | 62 | 18.0 | 154 | 23.5 | 35 | 20.8 | 42 | 22.3 | 16 | 20.5 | 39 | 15.5 | 20 | 20.6 | 12 | 19.4 | 53 | 17.4 | 435 | 20.2 |  |
|  | 5. Ex. imp. | 34 | 9.9 | 146 | 22.3 | 30 | 17.9 | 35 | 18.6 | 21 | 26.9 | 22 | 8.7 | 15 | 15.5 | 11 | 17.7 | 63 | 20.7 | 380 | 17.6 |  |

| **Sources of help by profession, in order of % rated ‘Very’ and ‘Extremely important’ combined for ‘All professions’ (n=2,180)** | | **Psych-**  **logist**  **(n=347)** | | **Nurse**  **(n=664)** | | **Occup-**  **ational**  **therapist**  **(n=171)** | | **Other**  **qualified**  **therapist**  **(n=189)** | | **Peer**  **support**  **worker**  **(n=80)** | | **Psychiatrist**  **(n=254)** | | **Social**  **worker**  **(n=97)** | | **Manager,**  **no prof.**  **qual. (n=63)** | | **Other**  **worker**  **(n=307)** | | **All**  **professions**  **(n=2,180)** | |
| --- | --- | --- | --- | --- | --- | --- | --- | --- | --- | --- | --- | --- | --- | --- | --- | --- | --- | --- | --- | --- | --- |
|  |  | **n** | **%*** | **n** | **%** | **n** | **%** | **n** | **%** | **n** | **%** | **n** | **%** | **n** | **%** | **n** | **%** | **n** | **%** | **n** | **%** |
| The support offered by local volunteers and mutual aid groups | 1. Not imp. | 80 | 23.2 | 87 | 13.2 | 16 | 9.4 | 42 | 22.2 | 13 | 16.3 | 61 | 24.2 | 8 | 8.3 | 8 | 12.9 | 53 | 17.4 | 372 | 17.2 |
|  | 2. Slightly | 114 | 33.0 | 149 | 22.6 | 38 | 22.2 | 48 | 25.4 | 16 | 20.0 | 83 | 32.9 | 22 | 22.7 | 15 | 24.2 | 58 | 19.1 | 544 | 25.1 |
|  | 3. Mod. | 71 | 20.6 | 153 | 23.3 | 37 | 21.6 | 33 | 17.5 | 17 | 21.3 | 64 | 25.4 | 20 | 20.6 | 13 | 21.0 | 58 | 19.1 | 467 | 21.6 |
|  | 4. Very | 45 | 13.0 | 131 | 19.9 | 43 | 25.2 | 35 | 18.5 | 15 | 18.8 | 29 | 11.5 | 24 | 24.7 | 10 | 16.1 | 70 | 23.0 | 404 | 18.7 |
|  | 5. Ex. imp. | 35 | 10.1 | 138 | 21.0 | 37 | 21.6 | 31 | 16.4 | 19 | 23.8 | 15 | 6.0 | 23 | 23.7 | 16 | 25.8 | 65 | 21.4 | 379 | 17.5 |
| Support and new initiatives from local voluntary sector organisations | 1. Not imp. | 88 | 25.4 | 97 | 14.8 | 16 | 9.5 | 41 | 21.9 | 11 | 13.8 | 69 | 27.6 | 11 | 11.3 | 8 | 12.9 | 56 | 18.2 | 401 | 18.6 |
|  | 2. Slightly | 113 | 32.7 | 156 | 23.8 | 36 | 21.4 | 44 | 23.5 | 18 | 22.5 | 84 | 33.6 | 17 | 17.5 | 13 | 21.0 | 58 | 18.9 | 541 | 25.0 |
|  | 3. Mod. | 67 | 19.4 | 141 | 21.5 | 42 | 25.0 | 34 | 18.2 | 19 | 23.8 | 57 | 22.8 | 25 | 25.8 | 15 | 24.2 | 55 | 17.9 | 456 | 21.1 |
|  | 4. Very | 49 | 14.2 | 136 | 20.7 | 34 | 20.2 | 36 | 19.3 | 14 | 17.5 | 26 | 10.4 | 24 | 24.7 | 9 | 14.5 | 72 | 23.5 | 401 | 18.6 |
|  | 5. Ex. imp. | 29 | 8.4 | 126 | 19.2 | 40 | 23.8 | 32 | 17.1 | 18 | 22.5 | 14 | 5.6 | 20 | 20.6 | 17 | 27.4 | 66 | 21.5 | 362 | 16.8 |
| National initiatives to support staff well-being | 1. Not imp. | 81 | 23.6 | 78 | 11.9 | 16 | 9.5 | 43 | 22.8 | 8 | 10.0 | 69 | 27.5 | 24 | 25.3 | 7 | 11.1 | 53 | 17.5 | 381 | 17.7 |
|  | 2. Slightly | 109 | 31.8 | 149 | 22.8 | 53 | 31.4 | 43 | 22.8 | 15 | 18.8 | 91 | 36.3 | 26 | 27.4 | 22 | 34.9 | 66 | 21.8 | 575 | 26.7 |
|  | 3. Mod. | 70 | 20.4 | 164 | 25.0 | 37 | 21.9 | 37 | 19.6 | 18 | 22.5 | 60 | 23.9 | 18 | 19.0 | 15 | 23.8 | 65 | 21.5 | 486 | 22.5 |
|  | 4. Very | 46 | 13.4 | 118 | 18.0 | 25 | 14.8 | 27 | 14.3 | 16 | 20.0 | 16 | 6.4 | 9 | 9.5 | 12 | 19.1 | 58 | 19.1 | 328 | 15.2 |
|  | 5. Ex. imp. | 37 | 10.8 | 146 | 22.3 | 38 | 22.5 | 39 | 20.6 | 23 | 28.8 | 15 | 6.0 | 18 | 19.0 | 7 | 11.1 | 61 | 20.1 | 386 | 17.9 |
| Information from the media or social media | 1. Not imp. | 33 | 9.5 | 119 | 18.0 | 16 | 9.5 | 24 | 12.7 | 10 | 12.7 | 21 | 8.3 | 4 | 4.2 | 9 | 14.5 | 59 | 19.3 | 297 | 13.7 |
|  | 2. Slightly | 106 | 30.6 | 205 | 31.1 | 55 | 32.5 | 65 | 34.4 | 21 | 26.6 | 76 | 29.9 | 30 | 31.3 | 22 | 35.5 | 89 | 29.1 | 670 | 30.9 |
|  | 3. Mod. | 131 | 37.9 | 220 | 33.3 | 62 | 36.7 | 55 | 29.1 | 23 | 29.1 | 93 | 36.6 | 34 | 35.4 | 21 | 33.9 | 86 | 28.1 | 730 | 33.7 |
|  | 4. Very | 61 | 17.6 | 78 | 11.8 | 22 | 13.0 | 29 | 15.3 | 17 | 21.5 | 48 | 18.9 | 21 | 21.9 | 9 | 14.5 | 46 | 15.0 | 331 | 15.3 |
|  | 5. Ex. imp. | 15 | 4.3 | 38 | 5.8 | 14 | 8.3 | 16 | 8.5 | 8 | 10.1 | 16 | 6.3 | 7 | 7.3 | 1 | 1.6 | 26 | 8.5 | 141 | 6.5 |
| *Percentages are of those who answered. The highest number of missing responses for a challenge was 25 (1.2% of total participants, n=2,180). This was for challenge: 'National initiatives to support service users and carers, such as helplines and online peer support', where n=2,155. | | | | | | | | | | | | | | | | | | | | | |
| ‘Not imp.’=’Not important’, ‘Mod.’=Moderately, ‘Ex. imp.’=’Extremely important’. | | | | | | | | | | | | | | | | | | | | | |

# Table 14: Sources of help by managerial role, in order of % rated ‘Very’ and ‘Extremely important’ combined for ‘All roles’ (n=2,180)

|  | | **Manager/ lead clinician (n=826)** | | **Non-manager/ lead clinician (n=1,350)** | | **All roles (n=2,180)** | |
| --- | --- | --- | --- | --- | --- | --- | --- |
|  |  | **n** | **%*** | **n** | **%** | **n** | **%** |
| Guidance from my employer on managing clinical and safety needs due to COVID-19 | 1. Not imp. | 29 | 3.5 | 37 | 2.7 | 66 | 3.0 |
|  | 2. Slightly | 75 | 9.1 | 165 | 12.2 | 240 | 11.0 |
|  | 3. Mod. | 152 | 18.4 | 306 | 22.7 | 459 | 21.1 |
|  | 4. Very | 259 | 31.4 | 445 | 33.0 | 704 | 32.3 |
|  | 5. Ex. imp. | 311 | 37.7 | 397 | 29.4 | 711 | 32.6 |
| Support and information from colleagues | 1. Not imp. | 12 | 1.5 | 25 | 1.9 | 37 | 1.7 |
|  | 2. Slightly | 78 | 9.5 | 119 | 8.8 | 198 | 9.1 |
|  | 3. Mod. | 198 | 24.1 | 333 | 24.7 | 532 | 24.5 |
|  | 4. Very | 273 | 33.2 | 449 | 33.4 | 723 | 33.3 |
|  | 5. Ex. imp. | 261 | 31.8 | 420 | 31.2 | 682 | 31.4 |
| Support and advice from my manager(s) | 1. Not imp. | 38 | 4.6 | 64 | 4.8 | 102 | 4.7 |
|  | 2. Slightly | 101 | 12.3 | 151 | 11.3 | 252 | 11.6 |
|  | 3. Mod. | 163 | 19.8 | 278 | 20.7 | 441 | 20.3 |
|  | 4. Very | 251 | 30.5 | 405 | 30.2 | 659 | 30.4 |
|  | 5. Ex. imp. | 270 | 32.8 | 444 | 33.1 | 715 | 33.0 |
| Adoption of new digital ways of working | 1. Not imp. | 29 | 3.5 | 90 | 6.7 | 119 | 5.5 |
|  | 2. Slightly | 67 | 8.2 | 167 | 12.5 | 234 | 10.8 |
|  | 3. Mod. | 167 | 20.3 | 322 | 24.0 | 490 | 22.6 |
|  | 4. Very | 232 | 28.3 | 361 | 26.9 | 595 | 27.5 |
|  | 5. Ex. imp. | 326 | 39.7 | 400 | 29.9 | 727 | 33.6 |
| Resilience and resourcefulness in adversity among service users and carers | 1. Not imp. | 20 | 2.4 | 60 | 4.4 | 80 | 3.7 |
|  | 2. Slightly | 74 | 9.0 | 167 | 12.4 | 242 | 11.1 |
|  | 3. Mod. | 220 | 26.6 | 328 | 24.3 | 549 | 25.2 |
|  | 4. Very | 218 | 26.4 | 404 | 29.9 | 623 | 28.6 |
|  | 5. Ex. imp. | 294 | 35.6 | 391 | 29.0 | 686 | 31.5 |
| Guidance disseminated by the NHS or professional bodies | 1. Not imp. | 14 | 1.7 | 29 | 2.2 | 43 | 2.0 |
|  | 2. Slightly | 73 | 8.9 | 200 | 14.9 | 273 | 12.6 |
|  | 3. Mod. | 215 | 26.1 | 357 | 26.6 | 574 | 26.5 |
|  | 4. Very | 250 | 30.4 | 426 | 31.8 | 677 | 31.2 |
|  | 5. Ex. imp. | 271 | 32.9 | 328 | 24.5 | 600 | 27.7 |
| Being aware of public support for key workers | 1. Not imp. | 72 | 8.8 | 132 | 9.9 | 204 | 9.4 |
|  | 2. Slightly | 170 | 20.7 | 295 | 22.0 | 467 | 21.6 |
|  | 3. Mod. | 200 | 24.4 | 329 | 24.6 | 531 | 24.5 |
|  | 4. Very | 178 | 21.7 | 304 | 22.7 | 482 | 22.3 |
|  | 5. Ex. imp. | 201 | 24.5 | 280 | 20.9 | 481 | 22.2 |
| Staff well-being initiatives set up during COVID-19 in my workplace | 1. Not imp. | 95 | 11.6 | 214 | 16.0 | 309 | 14.3 |
|  | 2. Slightly | 168 | 20.6 | 331 | 24.7 | 501 | 23.2 |
|  | 3. Mod. | 195 | 23.9 | 300 | 22.4 | 495 | 22.9 |
|  | 4. Very | 177 | 21.7 | 242 | 18.1 | 420 | 19.4 |
|  | 5. Ex. imp. | 182 | 22.3 | 253 | 18.9 | 435 | 20.1 |
| National initiatives to support service users and carers, such as helplines and online peer support | 1. Not imp. | 95 | 11.7 | 207 | 15.5 | 302 | 14.0 |
|  | 2. Slightly | 207 | 25.4 | 314 | 23.5 | 522 | 24.2 |
|  | 3. Mod. | 210 | 25.8 | 297 | 22.2 | 508 | 23.6 |
|  | 4. Very | 149 | 18.3 | 264 | 19.8 | 415 | 19.3 |
|  | 5. Ex. imp. | 153 | 18.8 | 255 | 19.1 | 408 | 18.9 |

| **Sources of help by managerial role, in order of % rated ‘Very’ and ‘Extremely important’ combined for ‘All roles’ (n=2,180)** | | **Manager/ lead clinician (n=826)** | | **Non-manager/ lead clinician (n=1,350)** | | **All roles (n=2,180)** | |
| --- | --- | --- | --- | --- | --- | --- | --- |
|  |  | **n** | **%** | **n** | % | **n** | **%*** |
| New initiatives in NHS mental health services | 1. Not imp. | 85 | 10.4 | 175 | 13.1 | 260 | 12.1 |
|  | 2. Slightly | 176 | 21.6 | 304 | 22.7 | 481 | 22.3 |
|  | 3. Mod. | 213 | 26.1 | 387 | 28.9 | 602 | 27.9 |
|  | 4. Very | 184 | 22.6 | 250 | 18.7 | 435 | 20.2 |
|  | 5. Ex. imp. | 157 | 19.3 | 223 | 16.7 | 380 | 17.6 |
| The support offered by local volunteers and mutual aid groups | 1. Not imp. | 133 | 16.2 | 238 | 17.8 | 372 | 17.2 |
|  | 2. Slightly | 210 | 25.6 | 334 | 24.9 | 544 | 25.1 |
|  | 3. Mod. | 186 | 22.6 | 279 | 20.8 | 467 | 21.6 |
|  | 4. Very | 154 | 18.7 | 250 | 18.7 | 404 | 18.7 |
|  | 5. Ex. imp. | 139 | 16.9 | 239 | 17.8 | 379 | 17.5 |
| Support and new initiatives from local voluntary sector organisations | 1. Not imp. | 149 | 18.2 | 252 | 18.9 | 401 | 18.6 |
|  | 2. Slightly | 214 | 26.1 | 326 | 24.4 | 541 | 25.0 |
|  | 3. Mod. | 178 | 21.7 | 276 | 20.6 | 456 | 21.1 |
|  | 4. Very | 147 | 17.9 | 253 | 18.9 | 401 | 18.6 |
|  | 5. Ex. imp. | 132 | 16.1 | 230 | 17.2 | 362 | 16.8 |
| National initiatives to support staff well-being | 1. Not imp. | 131 | 16.1 | 250 | 18.7 | 381 | 17.7 |
|  | 2. Slightly | 209 | 25.7 | 364 | 27.2 | 575 | 26.7 |
|  | 3. Mod. | 200 | 24.6 | 285 | 21.3 | 486 | 22.5 |
|  | 4. Very | 127 | 15.6 | 201 | 15.0 | 328 | 15.2 |
|  | 5. Ex. imp. | 147 | 18.1 | 239 | 17.9 | 386 | 17.9 |
| Information from the media or social media | 1. Not imp. | 111 | 13.5 | 185 | 13.8 | 297 | 13.7 |
|  | 2. Slightly | 246 | 29.9 | 422 | 31.4 | 670 | 30.9 |
|  | 3. Mod. | 305 | 37.1 | 424 | 31.6 | 730 | 33.7 |
|  | 4. Very | 114 | 13.9 | 217 | 16.2 | 331 | 15.3 |
|  | 5. Ex. imp. | 46 | 5.6 | 95 | 7.1 | 141 | 6.5 |
| *Percentages are of those who answered. The highest number of missing responses for a challenge was 25 (1.2% of total participants, n=2,180). This was for challenge: 'National initiatives to support service users and carers, such as helplines and online peer support', where n=2,155. | | | | | | | |
| ‘Not imp.’=’Not important’, ‘Mod.’=Moderately, ‘Ex. imp.’=’Extremely important’. | | | | | | | |

# Table 15: Staff-reported service changes

| **Setting** | **Question** | **Response option (top commonly reported changes, with frequencies)** | **n** | **%*** |
| --- | --- | --- | --- | --- |
| Crisis assessment services (n=308) | Have any changes occurred to your service’s hours and location? (n=250) | Yes | 133 | 53.2 |
|  |  | No | 117 | 46.8 |
|  | What changes have occurred (open-ended question)? Top commonly reported changes, with frequencies | Services reporting increased working hours or days (e.g. evening or weekend provision) (30) | - | - |
|  |  | New services being delivered (e.g. Mental Health Assessment Unit) (21) | - | - |
|  |  | Reduced service provision (e.g. reduced hours) (16) | - | - |
|  |  | Service has changed to be delivered completely remotely or digitally (4) | - | - |
|  | Are you continuing to visit service users at home? (n=254) | Yes, as usual | 31 | 15 |
|  |  | Yes, if strictly necessary | 161 | 77.8 |
|  |  | No | 15 | 7.3 |
|  |  | N/A - We don't usually do this | 47 | - |
|  | Has any new form of crisis service been established to meet local needs during the COVID-19 pandemic? (n=247) | Yes | 114 | 46.2 |
|  |  | No | 133 | 53.9 |

*Table 15 continues on next page*

| **Staff-reported service changes (continued)** | | | | |
| --- | --- | --- | --- | --- |
| **Setting** | **Question** | **Response option (top commonly reported changes, with frequencies)** | **n** | **%*** |
| Community teams and psychological treatment services (n=1,268) | Have any changes occurred to your service’s hours and location? (n=1,071) | Yes | 694 | 64.8 |
|  |  | No | 377 | 35.2 |
|  | What changes have occurred (open-ended question)? Top commonly reported changes, with frequencies | Service has changed to be delivered completely remotely or digitally (291) | - | - |
|  |  | Services reporting increased working hours or days (e.g. evening or weekend provision) (117) | - | - |
|  |  | Reduced service provision (e.g. no group programmes) (51) | - | - |
|  |  | New services being delivered (e.g. new child and adolescent mental health helpline) (16) | - | - |
|  | Are you continuing to visit clients? (n=1,075) | Yes, as usual | 21 | 2.4 |
|  |  | Yes, if strictly necessary | 544 | 60.8 |
|  |  | No | 330 | 36.9 |
|  |  | N/A - We don't usually do this | 180 | - |
|  | Are you continuing to meet face to face with clients? (n=1,077) | Yes, as usual | 16 | 1.5 |
|  |  | Yes, if strictly necessary | 588 | 55.2 |
|  |  | No | 462 | 43.4 |
|  |  | N/A - We don't usually do this | 11 | - |
|  | Are you offering psychological treatment by phone or by video call as a substitute for face to face appointments? (n=1,071) | Yes, aiming to conduct full psychological treatment by phone or video call | 518 | 55.1 |
|  |  | Yes, but in an abbreviated form | 352 | 37.5 |
|  |  | No, not usually | 70 | 7.5 |
|  |  | N/A | 131 | - |
|  | Have you started making more use of digital tools for assessment or treatment (internet-based or apps) as a substitute for face to face approaches? (n=1,072) | Yes | 525 | 49.0 |
|  |  | No | 547 | 51.0 |
| Community groups (n=155) | Are you providing any online group programme during the COVID-19 crisis? (n=87) | Yes | 45 | 51.7 |
|  |  | No | 42 | 48.3 |
|  | Are you continuing any other service or client contact during the current COVID-19 pandemic? (n=74) | Yes | 47 | 63.5 |
|  |  | No | 27 | 36.5 |
| *Percentages are of those who answered and did not provide a not applicable response. The missing rate was substantially higher for staff from community groups, where 68 (43.9%) and 81 (52.3%) out of 155 responses were missing. The question with next highest missing rate was 'Has any new form of crisis service been established to meet local needs during the COVID-19 pandemic?', where 61 (19.8%) responses were missing from 308 staff working in crisis assessment services. | | | | |

# Table 16: What are the main innovations and changes made to services as a result of the pandemic? (Open-ended questions)

**Summary**

The use of remote technologies, including video conferencing software and increased telephone contact, was the most frequently reported innovation. These have been used to enable staff to maintain contact with their teams and to conduct check-ins and therapeutic work (including virtual groups) with service users while working from home. Participants also described, albeit less frequently, that services had developed online webinars, activity packs, and COVID-19-related self-help guides for their service users. Other adaptations included extended service hours and electronic prescribing. Services in which in-person contact was unavoidable, such as in hospital, have adapted to promote social distancing and infection control, for example by marking 2m intervals for medication queues, introducing isolation wards, and adjusting staff rotas.

Participants also described the introduction of new services: new crisis support lines, helplines for members of the public, crisis hubs (which act as a single point of access), assessment hubs (which divert cases from A&E), and centralised referral points to triage cases across mental health services.

Innovations for staff welfare were frequently reported, including the introduction of ‘wobble’ rooms and staff helplines, increased supervision and wellbeing check-ins, and more use of informal supports (such as WhatsApp groups and virtual lunches). Participants described regular Trust briefings, team meetings, and information sessions relating to COVID-19.

| **Theme** | **Frequency*** | **Theme description** | **Indicative quotes** |
| --- | --- | --- | --- |
| Main Theme: Using Remote Technology* | 954 |  |  |
| Sub-theme: Use for communication with colleagues | 243 | Many participants reported being able to connect with their teams while working from home due to videoconferencing software. Also frequently mentioned was the benefit of this software as means of maintaining social contact (e.g. lunch breaks, end of day support meetings) with other staff when working remotely.  WhatsApp was described as a means of staff staying in contact with their teams and as a source of informal peer support within staff networks. | *“Shared team lunches on Microsoft teams.”*  *“Using WhatsApp to chat to colleagues”* |
| **Theme** | **Frequency*** | **Theme description** | **Indicative quotes** |
| Sub-theme: Use for communication with service users | 215 | Participants reported increased use of video calls and telephone contacts.  Video calls (n=182) were reported to be have been used for assessments, individual and family therapies, and ward rounds. Telephone calls (n=174) were used to provide therapies and welfare checks.  While many services had discontinued groups, new online groups (n=57) had been implemented by some services, including peer support group and psychoeducational groups. Participants also reported the introduction of webinars for service users. | *“[We are using] Attend Anywhere technology to allow virtual 'face to face ' with clients"”*  *‘As a team we have been having meeting daily via video so that we can keep abreast of a rapidly changing situation’* |
| Sub-theme: IT Infrastructure | 205 | Some participants reported having been provided with IT equipment to be able to work remotely, others had been required to rely on their own personal IT equipment.  Satisfaction with IT infrastructure varied, with some reporting that old equipment and poor connection speeds meant that software (e.g. MS Teams) could not be used or connections adequately maintained for provision of remote therapies.  Participants reported that additional technology (iPads etc.) had been acquired for wards so patients could stay in touch with family and friends. | *"Our Trust has been very poor at getting adequate IT set up for all staff to enable people to work from home. Therefore, it has been difficult for a lot of staff to work productively from home. After 6 + weeks, we are now set up to work remotely"*  *“Wards have ordered tablets to ensure clients can maintain contact with family and friends via Skype”* |

| **Theme** | **Frequency*** | **Theme description** | **Indicative quotes** |
| --- | --- | --- | --- |
| Main Theme: Adaptations to usual service provision | 119 | Participants described a range of changes to existing services. These included extended service hours and days; reduced waiting times (e.g. for IAPT); adapted referral pathways; changes to staff responsibilities to provide better support to community and inhouse teams; and electronic prescribing. Several described the introduction of COVID-19-related anxiety and wellbeing programmes and providing resources and self-help materials for staff and service users to access online.  Adaptations to support infection control included new shift patterns and staff rotas to minimise travel and crowding in offices; introduction of COVID-19 isolation wards; providing tablets on wards to allow service users to speak with family members while visits were not permitted; marking medication queues at 2m intervals; and displaying COVID-19 information posters. | *"24/7 access to crisis instead of 9-5"*  *"Closure of approx. 20% of our wards. This was a long term transformation plan that literally took 3 days to achieve. Needed to bolster crisis and community services to support these patients, plus considerable support from social care"* |
| Main Theme: Innovations for staff support | 93 | Participants described the introduction of initiatives to improve staff wellbeing. These included the staff ‘wobble’ or relaxation rooms, mindfulness activities, and buddy systems, and staff support helplines. Also reported was an increase in the frequency of clinical supervision and “check-ins”. Participants also welcomed having been provided with free meals and discounts. | *"Free meals for staff, 'rest and recharge' hub"*  *"We have brought in additional team meetings / reflective space for teams to be able to 're-connect' which has felt hugely important.”* |
| Main Theme: New and adapted services | 170 | - | **-** |
| Sub-theme: New services | 89 | New services included the introduction of mental health assessment and crisis hubs, centralised referral points to triage cases across mental health services, additional grief and bereavement support, and support for homeless people housed in hotels because of the pandemic. | *"A crisis hub to divert service users without physical health needs away from ED in the main hospital, particularly out of hours."*  *"A centralised referral team to triage all referrals to adult mental health"* |
| Sub-theme: New telephone services | 61 | Many participants reported the introduction of new phone services, including out-of-hours helplines and 24-hr crisis lines. Helplines were available for crisis support, support for carers, friends and family members, and support for general population wellbeing. Additional professional lines have been set up to support NHS staff and as a centralised point for referrals or patient information. | *"Setting up of 'wellbeing hub' for members of public to phone if they have covid anxieties."*  *"Central phone line for patient referrals and queries"* |
| **Theme** | **Frequency*** | **Theme description** | **Indicative quotes** |
| Main Theme: Organisational initiatives | 226 | - | **-** |
| Sub-theme: Changes in handovers and communication structures including improved working arrangements across team | 137 | Participants noted that using video conferencing software while working remotely had made meetings and multidisciplinary team working easier in some settings, allowing wider groups to be involved in care and to share learning.  Staff reported receiving daily updates on guidance and service changes, increased contact with management and lead clinicians, and in some cases, regular remote Q&As with Trust CEOs and senior leadership. Few participants reported issues around lack of guidance. Where problems were reported, they included a lack of a clear plans for implementing government guidelines at a local level, lack of guidance for supporting specific groups of service users and being deployed to other services without adequate training. | *"Regular updates and guidance for staff from senior management on current pandemic, NHS guidance and staff wellbeing programmes and support tools… Daily web catch ups with the team when working remotely to encourage and allow support/guidance with caseload.”*  *"No advice or guidance about daily practicalities provided by management, we have to take vague suggestion and generic guidance and innovate locally to manage both client and staff wellbeing.”* |
| Sub-theme: Reduced bureaucracy/ increased flexibility | 65 | Many participants noted the speed at which changes had been implemented, particularly the adoption of remote working, reflecting that such change had previously been difficult or slow.  Some reported that they were benefiting from the flexibility remote working allowed, and suggested patients may also benefit from staff working more flexibly. | *"It was great how in a few days we got permissions to work from home which previously we were told would take ages. There has been greater flexibility in this sense, and this has been refreshing”*  *"Improved means of interaction and implementation with partners … Resolution of deeply entrenched challenges"* |

| **Theme** | **Frequency*** | **Theme description** | **Indicative quotes** |
| --- | --- | --- | --- |
| Sub-theme: Management or leadership | 57 | Most comments regarding management and leadership related to the increased communication from or visibility of management. Some participants praised supportive management approaches and attention to staff welfare. A smaller number of participants reported that meaningful leadership was lacking, and that teams were compensating through informal networks. | *“Team working remains a priority despite staff being scattered… the result of a very well thought through and managed approach to service provision and staff wellbeing by leads within the team. I doubt this will be recognised, it has been done without clear guidance from the NHS and is a quiet but very important achievement by the local leads."*  *"Increased informal supervision in absence of meaningful leadership"* |
| Sub-theme: New joint working arrangements across teams | 26 | There were a small number of reports of local mental health teams having combined to share resources, prioritise cases, and streamline support. | *"Memory service and older adults CMHT have combined resources to prioritise urgent cases."*  *“Amalgamation of crisis and liaison and community intensive support team in to one service stream and linking in with CPN services to provide seamless support and access to ongoing post crisis work"* |
| Main Theme: Use of digital tools for therapy and assessment etc. | 104 | Some services reported recommending wellbeing apps and online CBT programmes. Other forms of digital tools used include self-help or “workbook” exercises are used in sessions with a clinician. | *“Rollout of SilverCloud digital support services both self-led and supported.”* |
| Main Theme: Social support and activities | 50 | - | ***-*** |

| **Theme** | **Frequency*** | **Theme description** | **Indicative quotes** |
| --- | --- | --- | --- |
| Sub-theme: Supporting clients with food, other immediate practical problems | - | Participants reported supporting service users through the provision of food (e.g. delivering food from food banks to those socially isolating) and the delivery of medications to those unable to pick up or order medication. | *"Coordinating food and medicine supplies for vulnerable current and recently discharged patients."* |
| Sub-theme: Help for clients to occupy themselves (e.g. resource packs) | - | Participants reported providing ‘isolation packs’ to service users who are self-isolating due to COVID-19, both in wards and in the community. On wards occupational therapists provided more activities for self-isolating service users and staff had been given examples of safe activities, such as gardening. In the community, staff reported having distributed activity packs to service users. | *"Activity packs given out to patients on wards. Gardening resources donated and our department handing these out to the wards."* |
| Sub-theme: Use/awareness of community and voluntary sector initiatives | - | Participants described increased signposting and use of community and voluntary services. Some had proactively contacted shops, networks, and services regarding their service users’ needs. | *"Engaging with volunteer buddy system for meds shopping and social contact via phone.”*  *"Signposting to community support services such as NHS volunteers, [it would be] very helpful to have this service all the time!"* |
| Main Theme: Adaptations for specific patient groups | - |  |  |
| Patient group: Dementia/ older adults | - | Examples included information having been sent out to service users by post, helplines established for carers, and psychological/welfare support provided to care homes and care home staff. | *“We are working via the CMHT and offering support by phone to those with a diagnosis of dementia and sending out information by post. Most clients are not familiar with technology beyond phone usage so anything via internet has to be arranged with their families”* |

| **Theme** | **Frequency*** | **Theme description** | **Indicative quotes** |
| --- | --- | --- | --- |
| Patient group: Eating disorders | - | Participants reported that service users were using apps to monitor food intake, and that high-risk clinics had been set up facilitate face to face appointments when necessary. | *" We have set up a high risk clinic for eating disorder patients requiring face to face review & physical heath checks. It is run on minimum staff with only 1 patient at any one time to reduce contact. Patients can get blood tests, ECGs, weighed, physical obs, psychiatric, nursing and dietician review all in one appointment. It is allowing us to manage risk as best we can in the current circumstances … Patients are using apps to monitor their food intake and emotions and seem to be finding it useful"* |
| Patient group: Families and young people | - | Participants reported the development of information booklets for parents with mental health problems, tailored support for schools, and advice for children, parents, and schools on transitioning back to school. | *"Within our Helping Families Team we have [developed] an information booklet for other staff to help them keep the child in mind and help understand the impact of having children home will have on parents mental health”* |
| Patient group: Forensic/prison | - | Adaptations included new telephone protocols to enable prisoners to access mental health support. | *“We have created a telephone contact protocol to enable prisoners to contact a designated clinician via their in-cell phone on their clinician's external work approved mobile number at a specific appointment time. The means that we can maintain contact as well as social distancing whilst supporting our clients”* |
| Patient group: Intellectual disabilities | - | Development of easy to read COVID-19 information was reported. | *“Creating easy read coronavirus information for people with learning disabilities, creating Makaton sign videos explaining the coronavirus and government guidance, creation of symbols to support difficult conversations around the pandemic (including end of life). Also, advice and guidance on common difficulties being faced for carers/supporters. All made accessible through our website”* |

| **Theme** | **Frequency*** | **Theme description** | **Indicative quotes** |
| --- | --- | --- | --- |
| Patient group: Perinatal | - | Increased use of telephone and video consultations for established service users, with a note that this could not be the only means of engagement as parent-infant bonding could not be assessed remotely. | *"Increased use of telephone or video consultations for (not new) established patients for whom physical access is difficult can be helpful in some circumstances and should count toward a clinical contact. However, assessing infant-parent bond fully cannot be done remotely so this can never be the only way that we engage with patients."* |
| Patient group: Psychosis | - | Development of tailored self-isolation recovery packs. | *"We have developed a self-isolation recovery pack for service users who have severe and enduring mental health needs - focussed particularly on psychosis"* |
| Patient group: Substance misuse | - | Daily attendance by drug service at hotel providing accommodation for homeless service users, including assessment and supervised methadone. | *“Hotel - funded by the Council - to house clients who are vulnerable and who need to be shielded but who were either street homeless or in hostels with shared facilities... The drug service attends the hotel daily to assess people with drug problems and to provide on-site daily supervised methadone 7 days a week. Mental health service, in-house GP services and the Find and Treat Team (testing people for COVID-19) are all present in the hotel. All residents are having daily temperature checks. By having all these services at the hotel, clients do not need to leave to attend to their medical or mental health needs.”* |

*****Responses often did not differentiate between use of remote technology to maintain contact with colleagues and with service users. Frequency counts include only examples where use could be attributed to contact with colleagues.

# Table 17: Remote working views by setting* (n=1,443)

|  | | **Crisis assessment services (n=308)** | | **Community teams** (n=1,268)** | | **Total (n=1,443)** | |
| --- | --- | --- | --- | --- | --- | --- | --- |
|  |  | **n** | **%** | **n** | **%** | **n** | **%***** |
| Are you using telephone calls or video consultations to replace some or all face to face meetings with clients? | Yes | 219 | 83.0 | 1011 | 94.1 | 1149 | 92.0 |
|  | No | 45 | 17.1 | 63 | 5.9 | 100 | 8.0 |
| Telephone calls are often a satisfactory way to make an initial assessment | 1. Strongly disagree | 29 | 13.8 | 154 | 15.9 | 169 | 15.2 |
|  | 2. Disagree | 73 | 34.8 | 357 | 37.0 | 408 | 36.6 |
|  | 3. Neither agree/disagree | 41 | 19.5 | 164 | 17.0 | 199 | 17.9 |
|  | 4. Agree | 57 | 27.1 | 234 | 24.2 | 274 | 24.6 |
|  | 5. Strongly agree | 10 | 4.8 | 57 | 5.9 | 64 | 5.8 |
| Video consultations are often a satisfactory way to way to make an initial assessment | 1. Strongly disagree | 12 | 5.7 | 73 | 7.6 | 80 | 7.2 |
|  | 2. Disagree | 45 | 21.5 | 250 | 26.0 | 276 | 24.9 |
|  | 3. Neither agree/disagree | 65 | 31.1 | 257 | 26.7 | 311 | 28.0 |
|  | 4. Agree | 71 | 34.0 | 312 | 32.4 | 364 | 32.8 |
|  | 5. Strongly agree | 16 | 7.7 | 70 | 7.3 | 78 | 7.0 |
| Telephone calls are often a satisfactory way to assess the progress of someone already known to the team | 1. Strongly disagree | 3 | 1.4 | 17 | 1.8 | 20 | 1.8 |
|  | 2. Disagree | 21 | 10.0 | 102 | 10.6 | 113 | 10.1 |
|  | 3. Neither agree/disagree | 30 | 14.3 | 153 | 15.8 | 177 | 15.9 |
|  | 4. Agree | 129 | 61.4 | 567 | 58.6 | 658 | 59.0 |
|  | 5. Strongly agree | 27 | 12.9 | 128 | 13.2 | 147 | 13.2 |
| Video consultations are often a satisfactory way to assess the progress of someone already known to the team | 1. Strongly disagree | 1 | 0.5 | 9 | 0.9 | 10 | 0.9 |
|  | 2. Disagree | 15 | 7.2 | 67 | 7.0 | 77 | 7.0 |
|  | 3. Neither agree/disagree | 42 | 20.1 | 171 | 17.8 | 201 | 18.2 |
|  | 4. Agree | 113 | 54.1 | 535 | 55.8 | 617 | 55.8 |
|  | 5. Strongly agree | 38 | 18.2 | 177 | 18.5 | 201 | 18.2 |
| Telephone calls are a reasonable way to conduct psychological treatment | 1. Strongly disagree | - | - | 106 | 4.9 | - | - |
|  | 2. Disagree | - | - | 294 | 13.7 | - | - |
|  | 3. Neither agree/disagree | - | - | 232 | 10.8 | - | - |
|  | 4. Agree | - | - | 221 | 10.3 | - | - |
|  | 5. Strongly agree | - | - | 41 | 1.9 | - | - |
| Video calls are a reasonable way to conduct psychological treatment | 1. Strongly disagree | - | - | 56 | 2.6 | - | - |
|  | 2. Disagree | - | - | 149 | 7.0 | - | - |
|  | 3. Neither agree/disagree | - | - | 257 | 12.0 | - | - |
|  | 4. Agree | - | - | 337 | 15.7 | - | - |
|  | 5. Strongly agree | - | - | 78 | 3.6 | - | - |
| I hope to meet clients face to face just as much as before when the COVID-19 pandemic has finished | 1. Strongly disagree | 7 | 3.4 | 20 | 2.1 | 20 | 1.8 |
|  | 2. Disagree | 34 | 16.7 | 129 | 13.4 | 146 | 13.2 |
|  | 3. Neither agree/disagree | 29 | 14.2 | 124 | 12.9 | 145 | 13.1 |
|  | 4. Agree | 64 | 31.4 | 323 | 33.6 | 367 | 33.2 |
|  | 5. Strongly agree | 70 | 34.3 | 366 | 38.1 | 427 | 38.6 |
| I am interested in making more use of video consultations than previously once the COVID-19 pandemic has finished | 1. Strongly disagree | 11 | 5.3 | 75 | 7.8 | 82 | 7.4 |
|  | 2. Disagree | 35 | 17.0 | 164 | 17.0 | 190 | 17.1 |
|  | 3. Neither agree/disagree | 39 | 18.9 | 166 | 17.2 | 197 | 17.8 |
|  | 4. Agree | 74 | 35.9 | 394 | 40.8 | 443 | 40.0 |
|  | 5. Strongly agree | 47 | 22.8 | 166 | 17.2 | 197 | 17.8 |
| I am interested in making more use of telephone calls than previously once the COVID-19 pandemic is finished | 1. Strongly disagree | 7 | 3.4 | 88 | 9.1 | 92 | 8.3 |
|  | 2. Disagree | 42 | 20.4 | 237 | 24.6 | 266 | 24.0 |
|  | 3. Neither agree/disagree | 57 | 27.7 | 222 | 23.0 | 262 | 23.6 |
|  | 4. Agree | 74 | 35.9 | 327 | 33.9 | 381 | 34.4 |
|  | 5. Strongly agree | 26 | 12.6 | 91 | 9.4 | 108 | 9.7 |

| **Remote working by setting* (n=1,443)** | | **Crisis assessment services (n=308)** | | **Community teams** (n=1,268)** | | **Total (n=1,443)** | |
| --- | --- | --- | --- | --- | --- | --- | --- |
|  |  | **n** | **%** | **n** | **%** | **n** | **%***** |
| Using phone rather than face to face contact is not too much of a problem for establishing a rapport | 1. Strongly disagree | 36 | 17.4 | 182 | 19.0 | 205 | 18.6 |
|  | 2. Disagree | 68 | 32.9 | 355 | 37.0 | 400 | 36.2 |
|  | 3. Neither agree/disagree | 49 | 23.7 | 160 | 16.7 | 196 | 17.7 |
|  | 4. Agree | 44 | 21.3 | 209 | 21.8 | 246 | 22.3 |
|  | 5. Strongly agree | 10 | 4.8 | 53 | 5.5 | 58 | 5.3 |
| Using video consultation rather than face to face contact is not too much of a problem for establishing a rapport | 1. Strongly disagree | 20 | 9.6 | 91 | 9.6 | 106 | 9.6 |
|  | 2. Disagree | 60 | 28.7 | 277 | 29.1 | 315 | 28.6 |
|  | 3. Neither agree/disagree | 60 | 28.7 | 260 | 27.3 | 306 | 27.8 |
|  | 4. Agree | 57 | 27.3 | 278 | 29.2 | 319 | 29.0 |
|  | 5. Strongly agree | 12 | 5.7 | 47 | 4.9 | 54 | 4.9 |
| The clients I see are sometimes easier to reach via phone or video consultation | 1. Strongly disagree | 20 | 9.9 | 106 | 11.0 | 119 | 10.8 |
|  | 2. Disagree | 46 | 22.7 | 236 | 24.5 | 268 | 24.3 |
|  | 3. Neither agree/disagree | 55 | 27.1 | 213 | 22.1 | 255 | 23.1 |
|  | 4. Agree | 67 | 33.0 | 338 | 35.1 | 383 | 34.7 |
|  | 5. Strongly agree | 15 | 7.4 | 69 | 7.2 | 80 | 7.2 |
| Offering remote rather than face-to-face contacts has meant some clients have not been seen | 1. Strongly disagree | 16 | 7.8 | 44 | 4.6 | 55 | 5.0 |
|  | 2. Disagree | 39 | 19.0 | 147 | 15.3 | 170 | 15.4 |
|  | 3. Neither agree/disagree | 30 | 14.6 | 134 | 14.0 | 153 | 13.9 |
|  | 4. Agree | 84 | 41.0 | 447 | 46.6 | 508 | 46.1 |
|  | 5. Strongly agree | 36 | 17.6 | 188 | 19.6 | 217 | 19.7 |
| Email or text messaging is the best way to keep in touch with some of my clients | 1. Strongly disagree | 34 | 16.5 | 124 | 12.9 | 149 | 13.48 |
|  | 2. Disagree | 45 | 21.8 | 206 | 21.4 | 229 | 20.72 |
|  | 3. Neither agree/disagree | 56 | 27.2 | 249 | 25.9 | 293 | 26.52 |
|  | 4. Agree | 61 | 29.6 | 320 | 33.3 | 365 | 33.03 |
|  | 5. Strongly agree | 10 | 4.9 | 62 | 6.5 | 69 | 6.24 |
| I have the necessary equipment and support to be able to carry out video consultations | 1. Strongly disagree | 23 | 11.3 | 120 | 12.5 | 131 | 11.84 |
|  | 2. Disagree | 47 | 23.0 | 175 | 18.2 | 208 | 18.81 |
|  | 3. Neither agree/disagree | 15 | 7.4 | 108 | 11.2 | 119 | 10.76 |
|  | 4. Agree | 90 | 44.1 | 406 | 42.2 | 474 | 42.86 |
|  | 5. Strongly agree | 29 | 14.2 | 153 | 15.9 | 174 | 15.73 |
| The clients I see are generally difficult to engage through phone or video consultations | 1. Strongly disagree | 7 | 3.4 | 31 | 3.2 | 33 | 3.0 |
|  | 2. Disagree | 38 | 18.5 | 235 | 24.5 | 250 | 22.6 |
|  | 3. Neither agree/disagree | 76 | 37.1 | 334 | 34.8 | 393 | 35.6 |
|  | 4. Agree | 53 | 25.9 | 259 | 27.0 | 302 | 27.3 |
|  | 5. Strongly agree | 31 | 15.1 | 102 | 10.6 | 127 | 11.5 |
| I feel confident in using video consultations for client contacts | 1. Strongly disagree | 21 | 10.2 | 82 | 8.6 | 97 | 8.8 |
|  | 2. Disagree | 44 | 21.5 | 218 | 22.8 | 246 | 22.3 |
|  | 3. Neither agree/disagree | 48 | 23.4 | 214 | 22.3 | 251 | 22.8 |
|  | 4. Agree | 70 | 34.2 | 356 | 37.2 | 404 | 36.7 |
|  | 5. Strongly agree | 22 | 10.7 | 88 | 9.2 | 104 | 9.4 |
| Conference calls are a good way of conducting meetings between staff | 1. Strongly disagree | 10 | 4.8 | 51 | 5.3 | 56 | 5.1 |
|  | 2. Disagree | 36 | 17.3 | 200 | 20.9 | 223 | 20.2 |
|  | 3. Neither agree/disagree | 29 | 13.9 | 181 | 18.9 | 200 | 18.1 |
|  | 4. Agree | 85 | 40.9 | 343 | 35.8 | 409 | 37.1 |
|  | 5. Strongly agree | 48 | 23.1 | 182 | 19.0 | 216 | 19.6 |
| Video meetings (e.g. on Microsoft Teams) are a good way of conducting meetings between staff | 1. Strongly disagree | 3 | 1.4 | 24 | 2.5 | 26 | 2.3 |
|  | 2. Disagree | 16 | 7.7 | 80 | 8.3 | 93 | 8.4 |
|  | 3. Neither agree/disagree | 29 | 13.9 | 154 | 16.0 | 175 | 15.8 |
|  | 4. Agree | 80 | 38.3 | 421 | 43.8 | 478 | 43.1 |
|  | 5. Strongly agree | 81 | 38.8 | 282 | 29.3 | 337 | 30.4 |

| **Remote working by setting* (n=1,443)** | | **Crisis assessment services (n=308)** | | **Community teams** (n=1,268)** | | **Total (n=1,443)** | |
| --- | --- | --- | --- | --- | --- | --- | --- |
|  |  | **n** | **%** | **n** | **%** | **n** | **%***** |
| Please give an estimate of the percentage of clients you see with whom you now mainly have contact by video call | 0-20% | 89 | 61.8 | 407 | 53.4 | 475 | 54.5 |
|  | 21-40% | 14 | 9.7 | 55 | 7.2 | 66 | 7.6 |
|  | 41-60% | 14 | 9.7 | 78 | 10.2 | 89 | 10.2 |
|  | 61-80% | 15 | 10.4 | 81 | 10.6 | 91 | 10.5 |
|  | 81-100% | 12 | 8.3 | 141 | 18.5 | 150 | 17.2 |
| *A participant may work in more than one setting (e.g. an inpatient service and a crisis assessment service). Only staff working in crisis assessment services and/or community teams were asked these questions. | | | | | | | |
| **Community teams includes psychological treatment services. | | | | | | | |
| ***Percentages are of those who answered. The highest number of missing responses for a statement was 343 (23.8% of total participants, n=1,443). This was for statement: 'Using video consultation rather than face to face contact is not too much of a problem for establishing a rapport', where n=1,100. | | | | | | | |

# Table 18: What is working well with remote working and technology use? (Open-ended question)

**Summary**

Further questions were asked to explore what participants felt was working well in remote technology use (a summary of what they felt was not working well appears in the next table). Remote working was described by many participants as an essential means of continuing to provide some degree of support to people using services throughout the pandemic and to maintain collaborative team interactions across a distance. Participants provided multiple examples of innovative service adaptations, including the adoption of video conferencing or telephone appointments for client contacts, digitising support resources and increased use of online therapeutic programs. Many participants felt that remote technologies were an efficient and effective means of organising team contacts, often freeing up more staff time to support people using services. This in turn enabled many services to provide support to a greater number of individuals and to reduce the time that many people were waiting to receive that support. Other key benefits of remote working included the ease and convenience of accessing digital technologies for many staff members and for some people using services, and opportunities for rapid information sharing with both colleagues and clients. However, many participants also stressed the importance of context in describing the positive aspects of remote working and particular client groups who may derive the greatest benefits through this form of work. Remote communication may specifically be a useful form of support for clients who are already known to a service, primarily with less complex needs, for “light-touch” interventions or low intensity therapeutic approaches and follow-up appointments. The importance of preference, access and experience with using digital technologies of people using the service also emerged as key elements determining the suitability of this approach and positive experiences of remote working.

| **Theme** | **Frequency** | **Theme description** | **Indicative quotes** |
| --- | --- | --- | --- |
| Main theme: Resource efficiency | 258 | The most frequently noted benefit of remote working was increased resource efficiency within services, particularly with relation to time and financial costs. Many participants noted the positive aspects of saving time on travel and meetings, which allowed more time for other work and for some, increased their availability for people using services. | ***-*** |
| Sub-theme: Timely response | - | Participants frequently noted that having more time available through reducing time on meetings and travel meant that services were often able to respond more rapidly to the needs of people using services. | *“Telephone assessments can work well for the less complex referrals we receive from GP's and speeds up the service patient receives”*  *“When it works, you aren’t travelling, so this gives you more time to assess, record and refer.”* |
| **Theme** | **Frequency** | **Theme description** | **Indicative quotes** |
| Sub-theme: Mutual convenience | - | Participants noted the often-unexpected ease of accessing digital technologies, primarily for staff though also for some people using services. The speed at which both meetings and client contacts could be arranged were often referred to as key benefits of remote working. There was also frequent reference to convenience of using digital communication from home, both for staff members and for some people using services. This had the added benefit of increasing staff attendance at meetings.  (Within the whole of the survey sample, this is a minority of participants and most say it’s not as good). | *“Virtual meetings are efficient & can be arranged quickly”*  *"Much easier/better than expected. Thought this would be extremely difficult, especially with vulnerable/LD population, but surprised at how well it works. It's quicker, more efficient, can often get same amount of information as face to face appts- especially for follow up appointments for people who are more stable. “* |
| Sub-theme: Increasing contacts | - | Although it was noted that not all people using services had access to digital technologies, the time saved on each contact and ease of access to digital technology for a number of staff members also increased the number of client contacts and waiting times for people newly referred to services. | *“i have always had phone calls with patients as an option and i think it helps in terms of access to them, reduced DNAs, easier for me to 'fit' into other parts of my days eg when working from home”*  *“Increased use of [online therapy programme] - helpful in reducing wait times”* |
| Main theme: Maintaining contact - the “best alternative” | 60 | Many participants reported feeling that remote appointments could not replace face-to-face work in terms of value for therapeutic relationships, but that digital contact was the best alternative in the context of the pandemic, and was currently crucial to keeping services going. | *-* |

| **Theme** | **Frequency** | **Theme description** | **Indicative quotes** |
| --- | --- | --- | --- |
| Sub-theme: Continuity of care | - | It was felt by a number of participants that remote appointments were an effective means of continuing to support their clients, while minimizing risks of infection to both staff and people using services. Many noted that a continuation of support would not otherwise have been possible. | *“With staff working from home means they can remain in contacts with clients: maintains some continuity of care and coverage of clients under the services”*  *“Being able to offer a service at this time. Being able to respond to the needs of our service users and carers”* |
| Sub-theme: Information sharing | - | Many participants reported that remote communication, enhanced by digital tools, was useful to share information and resources with both staff and clients across a distance. There were multiple references to digital tools that were found useful. | *“I have been sharing with my clients, useful and/or approved websites and guides recommended by my organization or the NHS Therapy service I work with. This contact and information has been well received and allowed me opportunities to engage with my client group.”*  *“[digital tool], it works well as we can use white board and share screen to share tools with patients. Some people that are not tech savvy struggle accessing support”* |
| Main theme: The importance of context | 37 | Many participants felt that remote communication was effective in specific contexts, ideally used selectively | *-* |
| Sub-theme: Clients known to services and/or less complex needs | **-** | For many participants, remote communication represented an effective means of contacting clients already known to the service. Remote consultation was often described as a useful tool for brief contacts as opposed to more in-depth therapeutic work or for clients with lower support needs. | *“We don't feel we can do a full mental health assessment like this, when we can't properly see and hear the subtle and non-verbal behaviours etc. it's much easier with people who already have an established relationship with us.”*  *“It works well with a few clients that are stable and already have rapport established with the team. Younger clients tend to respond better to this way of communicating.”* |
| **Theme** | **Frequency** | **Theme description** | **Indicative quotes** |
| Sub-theme: Ensuring access and experience of using digital tools | - | Participants most frequently noted the benefits of remote communication for clients with access to and experience of using remote technologies such as video calls: the importance of selectiveness in future use of video calls was emphasised, with such technology unlikely to be useful for everyone. | *“Patients continue to engage well with video calls if they have access to them.”*  *“Telephone consultations - very successful, although often with parents rather than the young people. Video calls can be very successful …for those who can manage the technology also very successful.”* |
| Sub-theme: Emerging preferences | **-** | Many participants also stressed that the benefits of remote communication also depended on the preferences of their clients for using these tools and methods. Remote approaches on the whole were seen as a positive alternative to face to face support for those who felt comfortable using these methods of communication. It was also felt to be important to allow service users to choose which forms of remote technology they were happy to use. | *“To try to see people digitally for the sake of monthly figures however some people do not like this means of communication and prefer telephone support. It should be led by service user preference and not service/ funding provider preference”*  *“both phone and video calling. It has been mixed, some service users do not want to access services using means other than face to face, others are happy to.”* |

# Table 19: What’s Not Working with Remote Working? (Open-ended question)

**Summary:**

Here we summarise answers to a question about what participants found was not working well with remote working (complementing answers in Table 18 about what is working well). Remote working referred to both home working and providing a service to service users from a work base but using remote working methods, such as telephone calls or video calling.

Home working issues included difficulties in accessing the necessary IT, internet connection to work from home, IT equipment being scarce, outdated and software being of lower quality and a barrier for many service users who used other apps. Remote working raised an accessibility issue in terms of service users with cognitive impairments, dementia or severely unwell. Management was criticised for lack of support of home working, distrust of employees, poor decision making and conflicting messages between management & executives. Practical issues included home distractions, lack of childcare, lack of adequate space, office furniture and potential to impact on mental and physical health. Feelings of isolation, blurring of home/work boundaries and feeling less effective were also mentioned.

Negative aspects of remote working from service users’ perspectives included difficulty accessing a service, due to issues with IT (lack of knowledge, lack of equipment, use of different platforms or apps than they were used to, lack, of privacy to make calls and lack of funds/access to Wi-Fi/data. Some service user prefer telephone, others video calling. Where a range is not offered some service users do not access a service. The therapeutic relationship with service users was also impacted, with impaired access to body language and eye contact and greater difficulty forming a rapport, especially when service users and staff did not already know each other.

| **Theme** | **Frequency** | **Theme description** | **Indicative quotes** |
| --- | --- | --- | --- |
| Impact of IT infrastructure | 273 | Impacts of IT infrastructure on remote working including challenges related to internet connectivity, lack of access to software and physical equipment, e.g. reliance on personal laptops. | *“It's not working - connectivity is poor & equipment not good enough”*  *“Managing Lockdown with a dated mobile & computer (no webcam for skype etc.”* |
| Impact on communication/ therapeutic relationship | 159 | Impacts of remote working that clinicians have noticed have impacted on the therapeutic relationship with service users. This was linked to the impact on the quality of contact with service users. Whilst more people could be contacted due to factors such as decreased travel time and shorter interactions, many participants commented on the diminished quality of these interactions. | *“Although we do everything online, I personally find it difficult because of difficulty to communicate efficiently with people w/MH problems due to body language, lack of eye contact, difficulty to maintain the therapeutic relationship intact, invasion to personal space (both mine and their) and bad signal which can cause disruption in the appointment.”* |
| **Theme** | **Frequency** | **Theme description** | **Indicative quotes** |
| Service users have difficulty accessing services (e.g. lack of devices) | 57 | Staff reported that service users faced similar challenges accessing remote services including lack of access to equipment, technical knowledge, etc. This was salient for particular groups such as older adults, those with childcare responsibilities and those with challenging living condition, e.g. homelessness. | *“Some of my patients have no access to mobile phones/IT equipment, some are isolated at home with family members/young children and do not have any privacy for appointments”* |
| Service users are not comfortable with remote working | 21 | Staff reported that some service user are not comfortable with using remote services. This includes a wide range of potential reasons, including own mental health (e.g. paranoia), lacking privacy, or wanting a different form of communication. | *“Some patients paranoid of these kinds of methods”*  *“despite [video calls] being offered as an option, most patients have declined and opted for telephone consultations.”* |
| Increased workload as a result | 13 | Some staff noted that the move to remote working was challenging as it has led to increased workload and demands on staff to provide services to more service users. Examples included more frequent telephone contact in an effort to emotionally contain/hold service users in whilst face to face appointments are not an option. | *“7 day CMHT on a 5 day budget.”*  *“Asked to do three assessments a day for assessment team. Unmanageable due to follow up work for each one, plus usual workload.”* |
| Lack of training | 6 | Some staff reported that they had not received sufficient training to allow them to use technology facilitating remote working. | *“Attend anywhere video conferencing. Probably due to lack of training and personal reluctance to use.”* |

# Table 20: Has any innovation or change been made in mental health care that you would like to remain in place after the pandemic subsides? (Open-ended question)

**Summary**

There were two strong themes that emerged from participants' feedback to which innovations and changes they would like to remain in place post-pandemic. One related to remote methods, both in use with service users for consultations, therapy, and regular 'check-in' contact, and also in use amongst staff for team meetings and training. In use for service user contact, many staff cited benefits around increasing access, especially in specific groups where barriers had previously prevented access, such as having a disability or where anxiety had hindered travel. However, there was caution that future implementation should be considered only in some cases where appropriate, and be offered as a choice, with some stating that face to face contact was preferable. The theme around using remote methods, such as Microsoft Teams, amongst staff was linked to the second strong theme, a wish to continue working from home at least some of the time. Staff valued the many benefits to their well-being, productivity, and efficiency in being able to conduct some of their tasks away from the office space, whether administrative or client contact. Other reasons cited were around environmental benefits from reduced travel, cost savings, and the ability to work across multi-agencies more effectively. Linked to some of these findings was another theme around working more flexibly and reducing bureaucracy, leading to a more agile way of working and a more responsive service in some cases. Staff well-being emerged as its own theme with some participants reporting that they would like the recent initiatives to put in place to support well-being during the pandemic to continue. There was also feedback from some participants that new services put in place to support service users during the pandemic, such as 24/7 crisis lines and alternatives to A&E, were developments that they would like to see continued. A final theme identified was the increased visibility and vital role played by wider community and voluntary sector resources in providing essential practical and well-being support to service users. Participants urged for better funding to ensure their continuation.

| **Theme** | **Frequency** | **Theme description** | **Indicative quotes** |
| --- | --- | --- | --- |
| Continued selective use of remote working with some service users | 635 | A large number of participants stated that they would like to see a continuation of offering service user appointments remotely, and this included telephone, video calls, and other online means. Key benefits noted were about increasing access, improving choice, and decreasing the number of missed appointments. A number of participants talked about how they had seen an increase in access to services by people who had struggled with one or more factors, such as social anxiety, travel issues, and co-morbidities or disabilities. Some mentioned specific groups where barriers to access had been reduced, such as young people with severe anxiety, people living with agoraphobia, and people with a disability. There was also mention of being able to involve families and carers more closely in someone's care. Many participants made it very clear that they wished use of remote technology to be an option rather than a default, citing the many limitations discussed in Table 19. | *“Remote access to young people - some are reporting that they like it better and some saying remote would fit in with their life better than coming to a clinic or a community setting”*  *“Telephone consultant clinics have previously been seen as second rate, however this would open services for people with co-morbidities from a distance who cannot easily access help”* |
| **Theme** | **Frequency** | **Theme description** | **Indicative quotes** |
| Continued use of technologies like Microsoft Teams for communication among professionals | 354 | There was considerable feedback from participants about the benefits of continuing to use technology for meetings and training amongst professionals. In many cases, this was also linked to the 'More working from home' theme. In both cases, participants talked about cost and time savings, less travel, higher productivity and flexibility, and a positive impact on wellbeing. Other benefits cited were around increased collaboration with teams spread across a distance or with those from other sectors, and being able to attend ward rounds remotely. A few participants also referred to the remote update briefings delivered by senior management and stated that it helped them feel more connected, and that senior staff appeared more approachable. | *“Greater cooperation / reduced barriers across organisations (including health and social care). Use of technology to facilitate collaboration by making it easier for people to communicate across distances”*  *“More remote consultations More acceptance of attending CPA or ward rounds for inpatients out of area remotely team meetings remotely allow more to access organisation regular briefings in Skype make CEO and directors seem more real and approachable and involved”* |
| More working from home | 245 | Many participants cited the benefits to them of being allowed to work from home at least at times. These included: a significant reduction in travel time, and a consequential environmental benefit, cost, and reduction in stress levels; having more time to carry out necessary tasks; a positive effect on their mental wellbeing and work/life balance; and working more efficiently. However, many participants also talked about “appropriate” use of home working and wished ideally to continue with a mix between office and home working. | *“Options to work from home/remotely need to continue primarily because it reduces the time wasted in commuting & expenses related to it”*  *“Remote working has been working well for us, as most of our clinical contacts take place over the phone; a mix of office and remote working would be helpful”* |
| Greater flexibility and less bureaucracy | 107 | Participants reported valuing the ability to work more flexibly and responsively. This related to internal teamwork, the ability to work flexible hours and from home, and in how they worked with patients. Some participants also mentioned how the reduction of administrative tasks and bureaucracy both freed up time and resulted in more patient-centred decisions and care, and a greater freedom to make changes that addressed needs. | *“more flexible ways to deliver appointments”*  *“Being more focused on doing what is right for the patient and less on wrapping ourselves in so much bureaucracy that we often miss what matters or struggle to make responsive decisions”* |

| **Theme** | **Frequency** | **Theme description** | **Indicative quotes** |
| --- | --- | --- | --- |
| Continued consideration of staff wellbeing needs | 60 | A sizeable number of participants mentioned how they would like recent staff well-being and support initiatives developed in response to the pandemic to continue. These included staff wellbeing hubs, online support groups for peers/colleagues, confidential staff helplines, and areas for rest and recuperation. Some also stated that there should be guidance around staff well-being, as well the continuation of a more friendly tone in communications from senior management, and a message of being valued. | *“Emails coming from senior managers and Trust directors have taken on a much more humane tone, and consequently seem far more genuine than the usual bland, generic corporate speak”*  *“Staff well-being hubs - organisation should place more emphasis on staff well-being”* |
| More easily accessible services | 59 | Some participants reported that they would like to see a continuation of new services set up for service users and carers beyond the current pandemic. These included mental health urgent assessment centres and other alternatives to the Accident and Emergency department, ambulance services specifically for mental health needs, and a centralised hub in the community that enabled a single point of access to a range of support offers. There were also a number of comments around continuing with 24-hour coverage in support services, including crisis and other helplines, and seven-day community services. | *“The creation of the MH urgent assessment centres which reduce the length of time a patient spends in A&E”*  *“The opportunities for people to access support (health, social, practical) from a centralised place - eg the establishment of local HUBS with one centralised contact number for an area. The NHS trusts new helpline for all patients and carers.”* |
| More use of voluntary sector and wider community resources | 13 | Several participants discussed the vital role played by the voluntary sector and other community-based resources in the current pandemic, including online provision, in supporting patients with practical matters, as well as those related to health and mental wellbeing. Some of these were user-led or offered peer support. There was a call to fund, value and integrate these support offers into future planning. | *“More understanding about the vital role undertaken by third sector within mental health We now need to see appropriate funding follow the client! Many small charities are doomed following this crisis”*  *“making more use of our amazing local communities, and providing more financial support, and including them more in all aspects of our work “* |

#

# Table 21: Are you particularly concerned about any potential long-term consequences from the current pandemic? (Open-ended question)

**Summary**

The most common themes, in order of frequency, were: concerns around a potential rise in the incidence of mental health problems – especially anxiety, grief, self-harm/suicide and OCD; concerns about the societal impacts of the pandemic (wider health inequalities, unemployment and homelessness); concerns about trauma, and PTSD being experienced by both NHS staff and the general population. Particular concern was raised about users and general population experiencing complex grief due to being unable to say goodbye to loved ones. Several responders were especially worried that people who are usually long-term service users, and others in need, have not sought professional support due to fears about catching COVID-19, and, as such, may present with more severe illness, and worse long-term outcomes, in the future. Numerous participants were afraid that changes made in response to this crisis would be used to justify further service cuts; increased pressure for staff to work timetables they would not normally agree to. Significant concern about staff burnout and local services not being sufficiently equipped to deal with a rise in demand. Losing office space was associated with losing informal supervision and support from colleagues, as well as difficulty building relationships with service users especially in e.g. independent mental health advocacy work. Mental health staff were exacerbated by poor management and lack of consultation on major workplace changes.

Concerns about the longer-term societal effect of the pandemic and its potential effects on mental health included the impact on mothers during the perinatal period, children, potential rise in inter-familial violence and other forms of abuse, disruption to children and young people’s education, particularly for BAME and poorer students whose predicted grades may not reflect their potential. Several participants were concerned about racism and xenophobia, both managing the disproportionate direct impact of the pandemic on BAME staff and the wider societal prejudices which have been exposed. There was a general theme of loss present in the data, loss of physical spaces, loss of jobs and staff, potential loss of life due to increase in suicides, and increase in complex grief due to loss of loved ones to COVID-19.

| **Theme** | **Frequency** | **Theme description** | **Indicative quotes** |
| --- | --- | --- | --- |
| Main theme: Consequences of trauma and adversity at the time of the pandemic | 564 | Participants anticipate rise in PTSD, anxiety disorders, depression in general population associated with complex grief & bereavement; increase in addiction & substance misuse; backwards steps or exacerbated difficulties for current service users; and staff burnout, both in frontline staff working on COVID-19 and in mental health staff. Client groups of particular concern were those with neurodevelopmental disorders including autism and learning disability. | *“The mental health issues in young people and their families will rise significantly when lockdown is lifted Issues around bereavement, financial pressures and trauma will be significant for at least two years but the virus will continue to present as an on-going risk”*  *“I believe we will be seeing a significant increase in PTSD in frontline staff which I don’t feel adequately trained/emotionally prepared to provide the support they will need”* |
| **Theme** | **Frequency** | **Theme description** | **Indicative quotes** |
| Main theme: Concern about a later surge in help-seeking | 347 | Some services have had referrals drop as people “push through” the immediate crisis or avoid seeking help to reduce NHS burden, and expect these to come through later.  Significant concern for older adults: memory services and dementia diagnostics have paused services.  A large number of participants explained that they were concerned about a potential rise in the incidence of mental health problems, due to direct (dealing with trauma, grief and anxiety) and the indirect effects of the pandemic (job loss, lack of professional help seeking). | *“Our service is currently closed to new referrals and we can't reduce our waiting lists because we are helping other areas I'm concerned by the likely high amount of people who will need urgent therapy in the coming months, and our ability to support them in a timely matter”*  *“Worried about incidence of mental health issues arising from people not presenting to service (e.g. are we not detecting first episode psychosis cases because they're not coming into contact with people?) Also of incidence of anxiety, depression, and trauma”* |
| Main theme: Societal impact of the pandemic | 343 | Multiple concerns were raised about the impact on the economy, people’s jobs and relationships, and how this may affect their mental health. The largest concern about societal impacts was economic recession and its effect on mental health. Increase in domestic abuse was also frequently mentioned, as well as the impact on children’s education, particularly for those already at a disadvantage. Some participants mentioned worries about homeless people losing accommodation provided during the pandemic. Some were worried that certain services may not resume, especially voluntary sector community projects provided by organisations that may collapse financially during the crisis. | *“I am worried about the adolescents I work with who are BME, looked-after or would be considered working-class because their GCSE grades are likely to be based on biased predictions which do not take their full potential into account (whilst children at private schools are likely to be predicted higher grades, for example) This may have a significant opportunity cost for them”.*  *“Widening health inequalities e.g. social deprivation linked to highest risk, lower paid workers being those more likely to be unable to work from home, impact if increasing numbers of people on universal credit and associated fears/struggles.”* |
| Main theme: Concerns relating to the pandemic and its requirements being prolonged | 219 | The major difficulty was uncertainty regarding the length of time for which the pandemic is likely to continue, the possibility of further peaks, and continuing restrictions over an extended period. This made it very difficult to plan future mental health care response, with fears that arrangements put in place on an emergency basis could not be sustained. There were also particular worries for staff who have been advised to shield, and concerns about practicalities of getting to work on public transport as lockdown eases. | *“Big setbacks for some people and their mental health Many services we usually refer onto have closed temporarily and this means that waiting times will only be longer when they do reopen”* |
| **Theme** | **Frequency** | **Theme description** | **Indicative quotes** |
| Sub theme: Impact on the family | - | Several participants said they were concerned about rises in domestic abuse, child abuse and other safeguarding concerns from the home, which may continue and have longer term repercussions, especially because of impacts on children not currently at school.  Within the sub theme of the impact on the family, concerns were also raised about the impact of the pandemic on children, their education and social development; mothers and their ability to form bonds of attachment with their children, and birth trauma. | *“Epigenetic effect of the increased stress in pregnancy on the unborn child. The increase in domestic violence and child violence. The delay in receiving treatment in the time critical perinatal period - long-standing impact on mother infant relationship and the attachment of the child to the mother "*  *“Impact on the well-being of children Very young children - at risk of DV/ poor attachment which we cannot properly support in lockdown Teenage boys - socialise very directly by doing together Boys of that age don't sit & chat - they play football, go out on bikes They are different to girls who seem to be having the same conversations remotely as they'd have at school I see my son getting sadder & lonelier despite us trying to support his social contacts This is not healthy & I fear the effect on less well-resourced teens It is different to the strains of war - where communities grew closer Isolation is bad for socialisation There are also the educational impacts With two parents with ongoing jobs we aren't able to properly support learning.”* |
| Sub theme: Impact on staff | - | There was a recurrent theme about the impact of the pandemic on staff burnout, mental health and well-being, staff morale and the training of junior staff/students. There was a fear that staff may have managed to respond relatively effective to the crisis, but that this may not be sustainable. | *“Long term impact on mental health of all professionals, impact in mental health staff seems to be forgotten, staff are scared, feel isolated and uninformed”*  *“Very concerned re impact on work force Our org employs 70% peer support workers They have actually so far been very resilient We are very experienced at managing staff with MH problems but most employers are not”* |

| **Theme** | **Frequency** | **Theme description** | **Indicative quotes** |
| --- | --- | --- | --- |
| Main theme: The continued use of telemedicine even where it does not work well | 205 | A few participants expressed their concern about the use of telemedicine being difficult to adapt to and concerns about it replacing face-to-face services. Participants were concerned that continuing to work remotely means less access to informal support and supervision from their team, as well as making it difficult to build rapport with service users. This was particularly challenging for advocacy and perinatal services. Several responses cited fears that telemedicine will become a way of cutting costs in the future and that it will be continued on a blanket basis even where it is significantly worse than face to face communication. | *“Referrers continuing to use remote therapy because it’s cheaper to outsource than face to face therapy”*  *“Worry that the savings (time and travel costs) made from increased patient contact via telephone and online will encourage trusts to push more for this patient contact, which I don't feel will ever give me the information I can gain from a home visit.”* |
| Main theme: Fewer resources if austerity is renewed | 95 | A few responders expressed their concerns about a lack of sufficient funding in the mental health services, especially if austerity measures were renewed. Participants were worried that money used to respond to COVID-19 will be clawed back from existing mental health budgets, or that COVID-19 induced recession will mean renewed austerity. Several participants felt that changes or service reductions introduced due to lockdown might be used to justify this.  Participants felt the impact of the pandemic on staff morale could be intensified by poor management within (principally NHS) teams. They worried that managers might use the way people have stepped up in response to the crisis to erode workers’ rights or demand major changes, such as shift patterns moving to 24/7 working without consultation. | *“The health care system is chronically underfunded and I am worried that in the wake of the pandemic, austerity and privatisation policies will be pushed further. The pandemic is likely to be highly politicised”*  *“Senior managers have taken the opportunity to make a lot of significant changes to services, often with limited oversight it seems, and wit little consultation with clients [or] staff.”* |
| Sub theme: Impact on service delivery and availability | - | The impact of the pandemic of service delivery was also a notable concern – namely concerns about service reductions, lack of resources, staff redeployment and underfunding and the effect that this would have on the NHS’s ability to response adequately in the future. These comments did not refer to these issues arising due to changes to potential future austerity measures (see the corresponding theme above). | *“Lack of capacity to support people struggling with their mental health, especially in the voluntary sector as a lot of charities are likely to close and the NHS relies on signposting people to us perhaps not realising how precarious our situation is We were only able to keep our national helpline open last year by crowdfunding and it is still possible that we will not be able to fund it this year - there will be a lot less to signpost to"*  *"Inpatient wards have significantly reduced number of beds, meaning much higher needs within community Concerned may be fewer beds permanently and that decision making regarding who gets bed may remain at this criteria"* |
| **Theme** | **Frequency** | **Theme description** | **Indicative quotes** |
| Main theme: Persisting negative effects on equality and rights | 59 | Various concerns were expressed regarding longer term loss of rights and increase in inequalities. Concerns in this area included loss of visiting rights for inpatients, lack of rights protections in inpatient services, the disproportionate impact of COVID-19 on BAME communities (staff and patients), and an increase in racism and xenophobia. There was also concern for LGBT service users locked down with unsupportive families and particularly for trans patients unable to access hormones or surgery, creating disproportionate trauma for an already traumatised community. | *“loss of jobs and income creating poverty and exacerbating existing poverty”*  *“Prejudice against racial minorities especially Chinese.”* |
| Main theme: Concerns about long-term lack of contact following the pandemic* | 29 | Concern that some service users might completely fall off the radar and/or be unable to seek urgent help from their GP or A & E in mental or physical health crisis, leading to major physical injury or death. Some participants were also worried about discharges being either delayed or taking place prematurely due to the pandemic rather than being based on service users’ needs. | *“I think bodies will be found in the homes months after they have completed suicide.”*  *“Government guidelines (social distancing etc) will cause a lasting impacting on many service users' decisions to engage with services like before when restrictions ease.”* |
| Sub theme: Worsening outcomes for service users | - | Some voiced their concerns that the pandemic may lead to worsening outcomes for service users with chronic mental health problems, namely due to disengagement/ lack of access to services/ delayed diagnosis and interventions and the subsequent effects on prognosis. | *“Increases in Duration untreated psychosis, affecting treatment response and prognosis”* |

# Table 22: Top five rated current work challenges* for participants in each speciality**

| **Older adults (including dementia) (n=853)** | | **n** | **n rated very or ex. rel.** | **% rated very or ex. rel.** |
| --- | --- | --- | --- | --- |
| 1 | Having to adapt too quickly to new ways of working (C) | 844 | 464 | 55.0 |
| 2 | Difficulties supporting clients who do not have their usual level of family support (A) | 532 | 264 | 49.6 |
| 3 | Increased pressures because of reduced levels of social care, primary care, physical health and other community services supporting older people (A) | 530 | 257 | 48.5 |
| 4 | Difficulty engaging remotely with people with cognitive or sensory impairments (A) | 527 | 245 | 46.5 |
| 5 | The risk I or my colleagues could be infected with COVID-19 at work (C) | 852 | 376 | 44.1 |
| **Drug & alcohol problems (n=456)** | | | | |
| 1 | Clients who have difficulty adhering to current guidance on social distancing, self-isolation or shielding (A) | 259 | 143 | 55.2 |
| 2 | Having to adapt too quickly to new ways of working (C) | 452 | 245 | 54.2 |
| 3 | Increased relapses under stresses of the current situation (A) | 256 | 122 | 47.7 |
| 4 | The risk I or my colleagues could be infected with COVID-19 at work (C) | 452 | 212 | 46.9 |
| 5 | Having to respond to additional mental health needs that appear to result from COVID-19 (C) | 455 | 195 | 42.9 |
| **Children & adolescents (n=443)** | | | | |
| 1 | Concerns resulting from withdrawal of the support and structure usually provided by schools (A) | 310 | 198 | 63.9 |
| 2 | Having to adapt too quickly to new ways of working (C) | 440 | 244 | 55.5 |
| 3 | Challenges arising from increased family tension and conflicts (A) | 307 | 164 | 53.4 |
| 4 | Greater difficulty in planning care because of reductions in other services, including health visiting and social care services (A) | 307 | 142 | 46.3 |
| 5 | Difficulty engaging children in remote appointments (A) | 308 | 126 | 40.9 |
| **Forensic (n=365)** | | | | |
| 1 | Having to adapt too quickly to new ways of working (C) | 363 | 212 | 58.4 |
| 2 | The risk I or my colleagues could be infected with COVID-19 at work (C) | 361 | 201 | 55.7 |
| 3 | Having to respond to additional mental health needs that appear to result from COVID-19 (C) | 364 | 152 | 41.8 |
| 4 | Pressures resulting from the need to support colleagues through the stresses associated with the pandemic (C) | 362 | 170 | 47.0 |
| 5 | Service users no longer getting an acceptable service due to service reconfiguration because of COVID-19 (C) | 362 | 144 | 39.8 |

| **Top five rated current work challenges for participants,* for each patient group**** | | | | |
| --- | --- | --- | --- | --- |
| **Perinatal (n=363)** | | **n** | **n rated very or ex. rel.** | **% rated very or ex. rel.** |
| 1 | Having to adapt too quickly to new ways of working (C) | 362 | 214 | 59.1 |
| 2 | The risk I or my colleagues could be infected with COVID-19 at work (C) | 359 | 117 | 32.6 |
| 3 | Having to respond to additional mental health needs that appear to result from COVID-19 (C) | 363 | 159 | 43.8 |
| 4 | Pressures resulting from the need to support colleagues through the stresses associated with the pandemic (C) | 360 | 146 | 40.6 |
| 5 | Service users no longer getting an acceptable service due to service reconfiguration because of COVID-19 (C) | 363 | 137 | 37.7 |
| **Intellectual disabilities (n=648)** | | | | |
| 1 | Having to adapt too quickly to new ways of working (C) | 644 | 343 | 53.3 |
| 2 | Increased need following the withdrawal of educational and support services in the community (A) | 395 | 206 | 52.2 |
| 3 | Difficulty maintaining adequate levels of support for those with significant and complex needs (A) | 399 | 200 | 50.1 |
| 4 | Difficulty for service users in comprehending the current crisis and the resulting requirements (A) | 398 | 198 | 49.8 |
| 5 | Difficulty engaging people with intellectual disabilities and/or autism with remote appointments (A) | 395 | 179 | 45.3 |
| **Eating disorders (n=451)** | | | | |
| 1 | Having to adapt too quickly to new ways of working (C) | 446 | 248 | 55.6 |
| 2 | The risk I or my colleagues could be infected with COVID-19 at work (C) | 448 | 193 | 43.1 |
| 3 | Having to respond to additional mental health needs that appear to result from COVID-19 (C) | 451 | 196 | 43.5 |
| 4 | Pressures resulting from the need to support colleagues through the stresses associated with the pandemic (C) | 450 | 177 | 39.3 |
| 5 | Service users no longer getting an acceptable service due to service reconfiguration because of COVID-19 (C) | 448 | 177 | 39.5 |
| *Includes 'current work challenges' (C) for participants from all settings and 'additional work challenges' (A) specific to the speciality. **Participants may work with more than one patient group (e.g. with adults of working age and forensic). ***The 'Additional work challenges' (A) sections appear in the survey after the 'Current work challenges' (C). Therefore, the reduced n for A compared to C represents participants who completed the first sections of the survey but then did not go on to complete the later branched sections of the survey. Ex. rel. = Extremely relevant. | | | | |

# Table 23: Current work challenges specific to participants working with older adults (including dementia), in order of % rated ‘Very’ and ‘Extremely relevant’ combined (n=853)

|  | | **n** | **%*** |
| --- | --- | --- | --- |
| Difficulties supporting clients who do not have their usual level of family support (n=532) | 1. Not relevant | 125 | 23.5 |
|  | 2. Slightly | 42 | 7.9 |
|  | 3. Moderately | 101 | 19.0 |
|  | 4. Very | 115 | 21.6 |
|  | 5. Extremely relevant | 149 | 28.0 |
| Increased pressures because of reduced levels of social care, primary care, physical health and other community services supporting older people (n=530) | 1. Not relevant | 128 | 24.2 |
|  | 2. Slightly | 49 | 9.3 |
|  | 3. Moderately | 96 | 18.1 |
|  | 4. Very | 102 | 19.3 |
|  | 5. Extremely relevant | 155 | 29.3 |
| Difficulty engaging remotely with people with cognitive or sensory impairments (n=527) | 1. Not relevant | 181 | 34.4 |
|  | 2. Slightly | 23 | 4.4 |
|  | 3. Moderately | 78 | 14.8 |
|  | 4. Very | 79 | 15.0 |
|  | 5. Extremely relevant | 166 | 31.5 |
| Challenges supporting clients and staff in nursing homes and care homes where there is or may be COVID-19 infection (n=525) | 1. Not relevant | 254 | 48.4 |
|  | 2. Slightly | 31 | 5.9 |
|  | 3. Moderately | 53 | 10.1 |
|  | 4. Very | 70 | 13.3 |
|  | 5. Extremely relevant | 117 | 22.3 |
| Difficulty reaching clients and carers who are self-isolating or shielded currently (n=532) | 1. Not relevant | 204 | 38.4 |
|  | 2. Slightly | 58 | 10.9 |
|  | 3. Moderately | 81 | 15.2 |
|  | 4. Very | 78 | 14.7 |
|  | 5. Extremely relevant | 111 | 20.9 |
| Increased need for involvement in end of life planning (n=530) | 1. Not relevant | 270 | 50.9 |
|  | 2. Slightly | 65 | 12.3 |
|  | 3. Moderately | 48 | 9.1 |
|  | 4. Very | 50 | 9.4 |
|  | 5. Extremely relevant | 97 | 18.3 |
| *Percentages are of those who answered. The highest number of missing responses for a challenge was 328 (38.5% of participants working with older adults, n=853). This was for challenge: 'Challenges supporting clients and staff in nursing homes and care homes where there is or may be COVID-19 infection' where n=525. | | | |

# Table 24: Current work challenges specific to participants working with children and adolescents, in order of % rated ‘Very’ and ‘Extremely relevant’ combined (n=443)

|  | | **n** | **%*** |
| --- | --- | --- | --- |
| Concerns resulting from withdrawal of the support and structure usually provided by schools (n=310) | 1. Not relevant | 39 | 12.6 |
|  | 2. Slightly | 18 | 5.8 |
|  | 3. Moderately | 55 | 17.7 |
|  | 4. Very | 82 | 26.5 |
|  | 5. Extremely relevant | 116 | 37.4 |
| Challenges arising from increased family tension and conflicts (n=307) | 1. Not relevant | 33 | 10.8 |
|  | 2. Slightly | 31 | 10.1 |
|  | 3. Moderately | 79 | 25.7 |
|  | 4. Very | 70 | 22.8 |
|  | 5. Extremely relevant | 94 | 30.6 |
| Greater difficulty in planning care because of reductions in other services, including health visiting and social care services (n=307) | 1. Not relevant | 53 | 17.3 |
|  | 2. Slightly | 42 | 13.7 |
|  | 3. Moderately | 70 | 22.8 |
|  | 4. Very | 79 | 25.7 |
|  | 5. Extremely relevant | 63 | 20.5 |
| Difficulty engaging children in remote appointments (n=308) | 1. Not relevant | 71 | 23.1 |
|  | 2. Slightly | 53 | 17.2 |
|  | 3. Moderately | 58 | 18.8 |
|  | 4. Very | 52 | 16.9 |
|  | 5. Extremely relevant | 74 | 24.0 |
| Greater than usual safeguarding issues (n=309) | 1. Not relevant | 56 | 18.1 |
|  | 2. Slightly | 48 | 15.5 |
|  | 3. Moderately | 80 | 25.9 |
|  | 4. Very | 80 | 25.9 |
|  | 5. Extremely relevant | 45 | 14.6 |
| Greater difficulty than usual in initiating and continuing child protection and children in need processes (n=304) | 1. Not relevant | 100 | 32.9 |
|  | 2. Slightly | 55 | 18.1 |
|  | 3. Moderately | 65 | 21.4 |
|  | 4. Very | 53 | 17.4 |
|  | 5. Extremely relevant | 31 | 10.2 |
| *Percentages are of those who answered. The highest number of missing responses for a challenge was 139 (31.4% of participants working with children and adolescents, n=443). This was for challenge: 'Greater difficulty than usual in initiating and continuing child protection and children in need processes' where n=304. | | | |

# Table 25: Current work challenges for participants working in perinatal services, in order of rated % ‘Very’ and ‘Extremely relevant’ combined (n=363)

|  | | | **n** | | **%*** | |
| --- | --- | --- | --- | --- | --- | --- |
| Challenges assessing mother and infant relationships because of lack of direct access (n=208) | | 1. Not relevant | 86 | | 41.4 | |
|  |  | 2. Slightly | 13 | | 6.3 | |
|  |  | 3. Moderately | 19 | | 9.1 | |
|  |  | 4. Very | 36 | | 17.3 | |
|  |  | 5. Extremely relevant | 54 | | 26.0 | |
| Difficulty planning and monitoring treatment due to reduced social care services (n=209) | | 1. Not relevant | 74 | | 35.4 | |
|  |  | 2. Slightly | 32 | | 15.3 | |
|  |  | 3. Moderately | 36 | | 17.2 | |
|  |  | 4. Very | 35 | | 16.8 | |
|  |  | 5. Extremely relevant | 32 | | 15.3 | |
| Safeguarding procedures are more difficult than usual to mobilise (n=211) | | 1. Not relevant | 84 | | 39.8 | |
|  |  | 2. Slightly | 29 | | 13.7 | |
|  |  | 3. Moderately | 36 | | 17.1 | |
|  |  | 4. Very | 26 | | 12.3 | |
|  |  | 5. Extremely relevant | 36 | | 17.1 | |
| Difficulty planning and monitoring treatment due to reduced community midwife and health visitor services (n=211) | | 1. Not relevant | 85 | | 40.3 | |
|  |  | 2. Slightly | 32 | | 15.2 | |
|  |  | 3. Moderately | 32 | | 15.2 | |
|  |  | 4. Very | 34 | | 16.1 | |
|  |  | 5. Extremely relevant | 28 | | 13.3 | |
| Reduced access to maternity units to carry out assessments (n=211) | | 1. Not relevant | 113 | | 53.6 | |
|  |  | 2. Slightly | 13 | | 6.2 | |
|  |  | 3. Moderately | 26 | | 12.3 | |
|  |  | 4. Very | 30 | | 14.2 | |
|  |  | 5. Extremely relevant | 29 | | 13.7 | |
| Referrals to our service not made or delayed because of the COVID-19 crisis (n=210) | | 1. Not relevant | 89 | | 42.4 | |
|  |  | 2. Slightly | 24 | | 11.4 | |
|  |  | 3. Moderately | 39 | | 18.6 | |
|  |  | 4. Very | 27 | | 12.9 | |
|  |  | 5. Extremely relevant | 31 | | 14.8 | |
| Reduced opportunities to admit to mother and baby units (n=206) | | 1. Not relevant | 119 | | 57.8 | |
|  |  | 2. Slightly | 28 | | 13.6 | |
|  |  | 3. Moderately | 22 | | 10.7 | |
|  |  | 4. Very | 18 | | 8.7 | |
|  |  | 5. Extremely relevant | 19 | | 9.2 | |
| Challenges arising from maternal or infant COVID-19 infection (n=207) | | 1. Not relevant | 114 | | 55.1 | |
|  |  | 2. Slightly | 37 | | 17.9 | |
|  |  | 3. Moderately | 21 | | 10.1 | |
|  |  | 4. Very | 13 | | 6.3 | |
|  |  | 5. Extremely relevant | 22 | | 10.6 | |
| Children are too readily taken into care because of obstacles to making other assessment and management plans at the present time (n=208) | 1. Not relevant | | 169 | 81.3 | |  |
|  | 2. Slightly | | 18 | 8.7 | |  |
|  | 3. Moderately | | 12 | 5.8 | |  |
|  | 4. Very | | 4 | 1.9 | |  |
|  | 5. Extremely relevant | | 5 | 2.4 | |  |
| *Percentages are of those who answered. The highest number of missing responses for a challenge was 157 (43.3% of participants working in perinatal services, n=363). This was for challenge: 'Reduced opportunities to admit to mother and baby units' where n=304. | | | | | |  |

# Table 26: Current work challenges for participants working in forensic services, in order of % rated ‘Very’ and ‘Extremely relevant’ combined (n=365)

|  | | **n** | **%*** |
| --- | --- | --- | --- |
| Delayed discharges from forensic units because of restrictions in community leave or in access to community placements (n=227) | 1. Not relevant | 74 | 32.6 |
|  | 2. Slightly | 34 | 15.0 |
|  | 3. Moderately | 23 | 10.1 |
|  | 4. Very | 33 | 14.5 |
|  | 5. Extremely relevant | 63 | 27.8 |
| Greater obstacles to transferring prisoners into and out of the legal system (n=228) | 1. Not relevant | 104 | 45.6 |
|  | 2. Slightly | 25 | 11.0 |
|  | 3. Moderately | 33 | 14.5 |
|  | 4. Very | 22 | 9.7 |
|  | 5. Extremely relevant | 44 | 19.3 |
| Obstacles to assessing and treating patients because of reduced functioning of the legal system (n=226) | 1. Not relevant | 87 | 38.5 |
|  | 2. Slightly | 33 | 14.6 |
|  | 3. Moderately | 41 | 18.1 |
|  | 4. Very | 23 | 10.2 |
|  | 5. Extremely relevant | 42 | 18.6 |
| Greater seclusion because patients are not able to follow guidance (n=229) | 1. Not relevant | 110 | 48.0 |
|  | 2. Slightly | 28 | 12.2 |
|  | 3. Moderately | 27 | 11.8 |
|  | 4. Very | 32 | 14.0 |
|  | 5. Extremely relevant | 32 | 14.0 |
| Restricted access to prisons to manage mental health conditions among prisoners (n=226) | 1. Not relevant | 133 | 58.9 |
|  | 2. Slightly | 11 | 4.9 |
|  | 3. Moderately | 20 | 8.9 |
|  | 4. Very | 22 | 9.7 |
|  | 5. Extremely relevant | 40 | 17.7 |
| Patients/prisoners using COVID-19 as a weapon (e.g. by intentionally coughing on staff or other patients) (n=228) | 1. Not relevant | 120 | 52.6 |
|  | 2. Slightly | 25 | 11.0 |
|  | 3. Moderately | 25 | 11.0 |
|  | 4. Very | 19 | 8.3 |
|  | 5. Extremely relevant | 39 | 17.1 |
| Increased mental health morbidity and self-harm in prisons related to the current COVID-19 epidemic and its management (n=222) | 1. Not relevant | 131 | 59.0 |
|  | 2. Slightly | 23 | 10.4 |
|  | 3. Moderately | 20 | 9.0 |
|  | 4. Very | 19 | 8.6 |
|  | 5. Extremely relevant | 29 | 13.1 |
| *Percentages are of those who answered. The highest number of missing responses for a challenge was 143 (39.2% of participants working in forensic services, n=365). This was for challenge: 'Increased mental health morbidity and self-harm in prisons related to the current COVID-19 epidemic and its management' where n=222. | | | |

# Table 27: Current work challenges for participants working with people with intellectual disabilities, in order of % rated ‘Very’ and ‘Extremely relevant’ combined (n=648)

|  | | **n** | **%*** |
| --- | --- | --- | --- |
| Increased need following the withdrawal of educational and support services in the community (n=395) | 1. Not relevant | 89 | 22.5 |
|  | 2. Slightly | 32 | 8.1 |
|  | 3. Moderately | 68 | 17.2 |
|  | 4. Very | 83 | 21.0 |
|  | 5. Extremely relevant | 123 | 31.1 |
| Difficulty maintaining adequate levels of support for those with significant and complex needs (n=399) | 1. Not relevant | 93 | 23.3 |
|  | 2. Slightly | 38 | 9.5 |
|  | 3. Moderately | 68 | 17.0 |
|  | 4. Very | 104 | 26.1 |
|  | 5. Extremely relevant | 96 | 24.1 |
| Difficulty for service users in comprehending the current crisis and the resulting requirements (n=398) | 1. Not relevant | 60 | 15.1 |
|  | 2. Slightly | 57 | 14.3 |
|  | 3. Moderately | 83 | 20.9 |
|  | 4. Very | 86 | 21.6 |
|  | 5. Extremely relevant | 112 | 28.1 |
| Difficulty engaging people with intellectual disabilities and/or autism with remote appointments (n=395) | 1. Not relevant | 100 | 25.3 |
|  | 2. Slightly | 39 | 9.9 |
|  | 3. Moderately | 77 | 19.5 |
|  | 4. Very | 81 | 20.5 |
|  | 5. Extremely relevant | 98 | 24.8 |
| Difficulty maintaining adequate support for families looking after a child/young person/adult with intellectual disabilities and/or autism who displays challenging behaviour (n=394) | 1. Not relevant | 136 | 34.5 |
|  | 2. Slightly | 33 | 8.4 |
|  | 3. Moderately | 56 | 14.2 |
|  | 4. Very | 66 | 16.8 |
|  | 5. Extremely relevant | 103 | 26.1 |
| Concerns about discrimination in access to physical health care for COVID-10 (n=394) | 1. Not relevant | 150 | 38.1 |
|  | 2. Slightly | 57 | 14.5 |
|  | 3. Moderately | 55 | 14.0 |
|  | 4. Very | 61 | 15.5 |
|  | 5. Extremely relevant | 71 | 18.0 |
| Lack of high quality and relevant information for people with intellectual disabilities and/or autism about the COVID-19 pandemics and the requirements that result (n=398) | 1. Not relevant | 103 | 25.9 |
|  | 2. Slightly | 79 | 19.9 |
|  | 3. Moderately | 83 | 20.9 |
|  | 4. Very | 72 | 18.1 |
|  | 5. Extremely relevant | 61 | 15.3 |
| *Percentages are of those who answered. The highest number of missing responses for a challenge was 254 (39.2% of participants working with people with intellectual disabilities, n=648). This was for challenges: 'Difficulty maintaining adequate support for families looking after a child/young person/adult with intellectual disabilities and/or autism who displays challenging behaviour' and 'Concerns about discrimination in access to physical health care for COVID-19' where n=222. | | | |

# Table 28: Current work challenges for participants working with people with eating disorders, in order of % rated ‘Very’ and ‘Extremely relevant’ combined (n=451)

|  | | **n** | **%*** |
| --- | --- | --- | --- |
| Exacerbation of eating disorders by current conditions during the COVID-19 epidemic, including changes in access to food and in meal routines (n=234) | 1. Not relevant | 20 | 8.6 |
|  | 2. Slightly | 7 | 3.0 |
|  | 3. Moderately | 71 | 30.3 |
|  | 4. Very | 75 | 32.1 |
|  | 5. Extremely relevant | 61 | 26.1 |
| Difficulty maintaining treatment programmes remotely (n=230) | 1. Not relevant | 18 | 7.8 |
|  | 2. Slightly | 19 | 8.3 |
|  | 3. Moderately | 81 | 35.2 |
|  | 4. Very | 74 | 32.2 |
|  | 5. Extremely relevant | 38 | 16.5 |
| Difficulty monitoring weight remotely (n=233) | 1. Not relevant | 23 | 9.9 |
|  | 2. Slightly | 15 | 6.4 |
|  | 3. Moderately | 84 | 36.1 |
|  | 4. Very | 65 | 27.9 |
|  | 5. Extremely relevant | 46 | 19.7 |
| *Percentages are of those who answered. The highest number of missing responses for a challenge was 221 (49.0% of participants working with people with eating disorders, n=451). This was for challenge 'Difficulty maintaining treatment programmes remotely' where n=233. | | | |

# Table 29: Current work challenges for all participants by profession, in order of % rated ‘Very’ and ‘Extremely relevant’ combined for ‘All professions’ (n=2,180)

|  | | **Psych-**  **ologist (n=347)** | | **Nurse**  **(n=664)** | | **Occup-**  **ational**  **therapist**  **(n=171)** | | **Other**  **qualified**  **therapist**  **(n=189)** | | **Peer**  **support**  **worker**  **(n=80)** | | **Psych-**  **iatrist**  **(n=254)** | | **Social**  **worker**  **(n=97)** | | **Manager,**  **no prof.**  **qual.**  **(n=63)** | | **Other**  **worker**  **(n=307)** | | **All prof-**  **essions**  **(n=2,180)** | |
| --- | --- | --- | --- | --- | --- | --- | --- | --- | --- | --- | --- | --- | --- | --- | --- | --- | --- | --- | --- | --- | --- |
|  |  | **n** | **%*** | **n** | **%** | **n** | **%** | **n** | **%** | **n** | **%** | **n** | **%** | **n** | **%** | **n** | **%** | **n** | **%** | **n** | **%** |
| Having to adapt too quickly to new ways of working | 1. Not rel. | 13 | 3.8 | 21 | 3.2 | 5 | 2.9 | 10 | 5.3 | 4 | 5.0 | 22 | 8.7 | 7 | 7.4 | 5 | 8.1 | 23 | 7.5 | 112 | 5.2 |
|  | 2. Slightly | 73 | 21.4 | 101 | 15.3 | 27 | 15.9 | 31 | 16.4 | 15 | 18.8 | 60 | 23.7 | 16 | 16.8 | 9 | 14.5 | 53 | 17.4 | 389 | 18.0 |
|  | 3. Mod. | 79 | 23.2 | 129 | 19.5 | 43 | 25.3 | 37 | 19.6 | 15 | 18.8 | 64 | 25.3 | 11 | 11.6 | 15 | 24.2 | 59 | 19.3 | 452 | 20.9 |
|  | 4. Very | 83 | 24.3 | 166 | 25.1 | 43 | 25.3 | 51 | 27.0 | 19 | 23.8 | 49 | 19.4 | 27 | 28.4 | 13 | 21.0 | 87 | 28.5 | 539 | 24.9 |
|  | 5. Ex. rel. | 93 | 27.3 | 245 | 37.0 | 52 | 30.6 | 60 | 31.8 | 27 | 33.8 | 58 | 22.9 | 34 | 35.8 | 20 | 32.3 | 83 | 27.2 | 673 | 31.1 |
| The risk I or my colleagues could be infected with COVID-19 at work | 1. Not rel. | 71 | 20.6 | 25 | 3.8 | 12 | 7.0 | 92 | 49.2 | 13 | 16.3 | 11 | 4.4 | 2 | 2.1 | 12 | 19.1 | 68 | 22.4 | 309 | 14.3 |
|  | 2. Slightly | 92 | 26.7 | 111 | 16.8 | 43 | 25.2 | 43 | 23.0 | 12 | 15.0 | 58 | 22.9 | 12 | 12.6 | 17 | 27.0 | 57 | 18.8 | 446 | 20.6 |
|  | 3. Mod. | 81 | 23.5 | 171 | 25.8 | 51 | 29.8 | 21 | 11.2 | 14 | 17.5 | 92 | 36.4 | 39 | 41.1 | 14 | 22.2 | 56 | 18.4 | 540 | 24.9 |
|  | 4. Very | 57 | 16.5 | 172 | 26.0 | 26 | 15.2 | 17 | 9.1 | 18 | 22.5 | 49 | 19.4 | 24 | 25.3 | 11 | 17.5 | 42 | 13.8 | 416 | 19.2 |
|  | 5. Ex. rel. | 44 | 12.8 | 183 | 27.6 | 39 | 22.8 | 14 | 7.5 | 23 | 28.8 | 43 | 17.0 | 18 | 19.0 | 9 | 14.3 | 81 | 26.6 | 456 | 21.0 |
| Having to respond to additional mental health needs that appear to result from COVID-19 | 1. Not rel. | 24 | 6.9 | 57 | 8.6 | 10 | 6.0 | 19 | 10.1 | 9 | 11.4 | 26 | 10.3 | 10 | 10.3 | 10 | 16.1 | 45 | 14.7 | 213 | 9.8 |
|  | 2. Slightly | 98 | 28.3 | 150 | 22.7 | 36 | 21.4 | 39 | 20.6 | 14 | 17.7 | 68 | 26.9 | 22 | 22.7 | 18 | 29.0 | 71 | 23.1 | 519 | 23.9 |
|  | 3. Mod. | 103 | 29.8 | 169 | 25.5 | 53 | 31.6 | 45 | 23.8 | 19 | 24.1 | 86 | 34.0 | 26 | 26.8 | 9 | 14.5 | 68 | 22.2 | 578 | 26.6 |
|  | 4. Very | 62 | 17.9 | 136 | 20.5 | 40 | 23.8 | 38 | 20.1 | 17 | 21.5 | 43 | 17.0 | 22 | 22.7 | 12 | 19.4 | 74 | 24.1 | 445 | 20.5 |
|  | 5. Ex. rel. | 59 | 17.1 | 150 | 22.7 | 29 | 17.3 | 48 | 25.4 | 20 | 25.3 | 30 | 11.9 | 17 | 17.5 | 13 | 21.0 | 49 | 16.0 | 416 | 19.2 |
| Pressures resulting from the need to support colleagues through the stresses associated with the pandemic | 1. Not rel. | 36 | 10.4 | 43 | 6.5 | 11 | 6.5 | 30 | 15.9 | 10 | 12.5 | 19 | 7.5 | 5 | 5.3 | 1 | 1.6 | 57 | 18.8 | 215 | 9.9 |
|  | 2. Slightly | 92 | 26.7 | 150 | 22.8 | 40 | 23.5 | 59 | 31.2 | 20 | 25.0 | 82 | 32.5 | 18 | 19.0 | 14 | 22.6 | 72 | 23.7 | 551 | 25.5 |
|  | 3. Mod. | 90 | 26.1 | 160 | 24.4 | 52 | 30.6 | 34 | 18.0 | 20 | 25.0 | 67 | 26.6 | 24 | 25.3 | 20 | 32.3 | 79 | 26.0 | 546 | 25.3 |
|  | 4. Very | 55 | 15.9 | 170 | 25.9 | 40 | 23.5 | 38 | 20.1 | 11 | 13.8 | 52 | 20.6 | 27 | 28.4 | 13 | 21.0 | 56 | 18.4 | 462 | 21.4 |
|  | 5. Ex. rel. | 72 | 20.9 | 134 | 20.4 | 27 | 15.9 | 28 | 14.8 | 19 | 23.8 | 32 | 12.7 | 21 | 22.1 | 14 | 22.6 | 40 | 13.2 | 388 | 18.0 |
| Service users no longer getting an acceptable service due to service reconfiguration because of COVID-19 | 1. Not rel. | 46 | 13.3 | 93 | 14.1 | 22 | 12.9 | 23 | 12.2 | 18 | 22.8 | 28 | 11.2 | 8 | 8.4 | 14 | 22.2 | 58 | 19.0 | 313 | 14.5 |
|  | 2. Slightly | 86 | 24.9 | 136 | 20.6 | 36 | 21.1 | 54 | 28.7 | 14 | 17.7 | 68 | 27.1 | 18 | 19.0 | 11 | 17.5 | 70 | 22.9 | 496 | 22.9 |
|  | 3. Mod. | 90 | 26.1 | 163 | 24.7 | 41 | 24.0 | 47 | 25.0 | 12 | 15.2 | 73 | 29.1 | 20 | 21.1 | 17 | 27.0 | 75 | 24.5 | 540 | 24.9 |
|  | 4. Very | 64 | 18.6 | 139 | 21.1 | 38 | 22.2 | 36 | 19.2 | 18 | 22.8 | 46 | 18.3 | 36 | 37.9 | 12 | 19.1 | 57 | 18.6 | 446 | 20.6 |
|  | 5. Ex. rel. | 59 | 17.1 | 129 | 19.6 | 34 | 19.9 | 28 | 14.9 | 17 | 21.5 | 36 | 14.3 | 13 | 13.7 | 9 | 14.3 | 46 | 15.0 | 371 | 17.1 |
| Having to learn to use new technologies too quickly and/or without sufficient training and support | 1. Not rel. | 37 | 10.7 | 109 | 16.5 | 21 | 12.4 | 24 | 12.7 | 17 | 21.3 | 39 | 15.4 | 11 | 11.6 | 13 | 21.3 | 81 | 26.5 | 355 | 16.4 |
|  | 2. Slightly | 94 | 27.2 | 140 | 21.2 | 44 | 26.0 | 38 | 20.1 | 20 | 25.0 | 84 | 33.2 | 18 | 19.0 | 11 | 18.0 | 66 | 21.6 | 517 | 23.9 |
|  | 3. Mod. | 80 | 23.1 | 145 | 22.0 | 43 | 25.4 | 43 | 22.8 | 20 | 25.0 | 47 | 18.6 | 24 | 25.3 | 16 | 26.2 | 65 | 21.2 | 485 | 22.4 |
|  | 4. Very | 74 | 21.4 | 128 | 19.4 | 36 | 21.3 | 35 | 18.5 | 12 | 15.0 | 47 | 18.6 | 17 | 17.9 | 12 | 19.7 | 58 | 19.0 | 420 | 19.4 |
|  | 5. Ex. rel. | 61 | 17.6 | 137 | 20.8 | 25 | 14.8 | 49 | 25.9 | 11 | 13.8 | 36 | 14.2 | 25 | 26.3 | 9 | 14.8 | 36 | 11.8 | 389 | 18.0 |

| **Current work challenges for all participants by profession, in order of % rated ‘Very’ and ‘Extremely relevant’ combined for ‘All professions’ (n=2,180)** | | **Psych-**  **ologist (n=347)** | | **Nurse**  **(n=664)** | | **Occup-**  **ational**  **therapist**  **(n=171)** | | **Other**  **qualified**  **therapist**  **(n=189)** | | **Peer**  **support**  **worker**  **(n=80)** | | **Psych-**  **iatrist**  **(n=254)** | | **Social**  **worker**  **(n=97)** | | **Manager,**  **no prof.**  **qual.**  **(n=63)** | | **Other**  **worker**  **(n=307)** | | **All prof-**  **essions**  **(n=2,180)** | |
| --- | --- | --- | --- | --- | --- | --- | --- | --- | --- | --- | --- | --- | --- | --- | --- | --- | --- | --- | --- | --- | --- |
|  |  | **n** | **%*** | **n** | **%** | **n** | **%** | **n** | **%** | **n** | **%** | **n** | **%** | **n** | **%** | **n** | **%** | **n** | **%** | **n** | **%** |
| The risk family and friends may be infected with COVID-19 through me | 1. Not rel. | 100 | 28.9 | 54 | 8.2 | 21 | 12.3 | 100 | 53.2 | 18 | 22.8 | 29 | 11.5 | 13 | 13.5 | 15 | 24.2 | 83 | 27.2 | 436 | 20.1 |
|  | 2. Slightly | 98 | 28.3 | 139 | 21.1 | 57 | 33.3 | 42 | 22.3 | 14 | 17.7 | 62 | 24.5 | 17 | 17.7 | 16 | 25.8 | 55 | 18.0 | 503 | 23.2 |
|  | 3. Mod. | 62 | 17.9 | 150 | 22.7 | 40 | 23.4 | 16 | 8.5 | 12 | 15.2 | 80 | 31.6 | 35 | 36.5 | 15 | 24.2 | 48 | 15.7 | 458 | 21.1 |
|  | 4. Very | 43 | 12.4 | 143 | 21.7 | 20 | 11.7 | 17 | 9.0 | 16 | 20.3 | 39 | 15.4 | 16 | 16.7 | 8 | 12.9 | 46 | 15.1 | 348 | 16.1 |
|  | 5. Ex. rel. | 43 | 12.4 | 174 | 26.4 | 33 | 19.3 | 13 | 6.9 | 19 | 24.1 | 43 | 17.0 | 15 | 15.6 | 8 | 12.9 | 73 | 23.9 | 423 | 19.5 |
| The risk that COVID-19 will spread between service users I'm working with | 1. Not rel. | 147 | 42.6 | 101 | 15.3 | 39 | 23.1 | 117 | 61.9 | 26 | 32.5 | 35 | 13.8 | 18 | 18.6 | 23 | 37.1 | 110 | 36.0 | 619 | 28.5 |
|  | 2. Slightly | 65 | 18.8 | 132 | 20.0 | 39 | 23.1 | 25 | 13.2 | 8 | 10.0 | 52 | 20.6 | 18 | 18.6 | 13 | 21.0 | 47 | 15.4 | 403 | 18.6 |
|  | 3. Mod. | 56 | 16.2 | 135 | 20.5 | 27 | 16.0 | 18 | 9.5 | 13 | 16.3 | 57 | 22.5 | 28 | 28.9 | 10 | 16.1 | 34 | 11.1 | 378 | 17.4 |
|  | 4. Very | 33 | 9.6 | 126 | 19.1 | 21 | 12.4 | 11 | 5.8 | 17 | 21.3 | 50 | 19.8 | 25 | 25.8 | 9 | 14.5 | 50 | 16.3 | 342 | 15.8 |
|  | 5. Ex. rel. | 44 | 12.8 | 166 | 25.2 | 43 | 25.4 | 18 | 9.5 | 16 | 20.0 | 59 | 23.3 | 8 | 8.3 | 7 | 11.3 | 65 | 21.2 | 427 | 19.7 |
| Being expected to use new technologies without reliable access to necessary tools and equipment | 1. Not rel. | 69 | 20.1 | 159 | 24.1 | 34 | 20.0 | 35 | 18.8 | 27 | 33.8 | 40 | 15.8 | 22 | 22.7 | 19 | 30.7 | 115 | 37.5 | 524 | 24.2 |
|  | 2. Slightly | 85 | 24.8 | 147 | 22.3 | 47 | 27.7 | 47 | 25.3 | 18 | 22.5 | 83 | 32.8 | 14 | 14.4 | 9 | 14.5 | 59 | 19.2 | 510 | 23.6 |
|  | 3. Mod. | 62 | 18.1 | 124 | 18.8 | 40 | 23.5 | 32 | 17.2 | 13 | 16.3 | 48 | 19.0 | 22 | 22.7 | 14 | 22.6 | 59 | 19.2 | 415 | 19.2 |
|  | 4. Very | 65 | 19.0 | 116 | 17.6 | 30 | 17.7 | 29 | 15.6 | 10 | 12.5 | 41 | 16.2 | 18 | 18.6 | 11 | 17.7 | 37 | 12.1 | 359 | 16.6 |
|  | 5. Ex. rel. | 62 | 18.1 | 113 | 17.2 | 19 | 11.2 | 43 | 23.1 | 12 | 15.0 | 41 | 16.2 | 21 | 21.7 | 9 | 14.5 | 37 | 12.1 | 357 | 16.5 |
| Problems resulting from lack of access to testing | 1. Not rel. | 129 | 37.3 | 132 | 20.1 | 31 | 18.2 | 99 | 52.9 | 22 | 27.9 | 47 | 18.6 | 14 | 14.4 | 21 | 33.9 | 114 | 37.3 | 614 | 28.4 |
|  | 2. Slightly | 69 | 19.9 | 142 | 21.6 | 32 | 18.8 | 27 | 14.4 | 14 | 17.7 | 52 | 20.6 | 21 | 21.7 | 10 | 16.1 | 58 | 19.0 | 427 | 19.7 |
|  | 3. Mod. | 72 | 20.8 | 128 | 19.5 | 54 | 31.8 | 22 | 11.8 | 9 | 11.4 | 57 | 22.5 | 24 | 24.7 | 13 | 21.0 | 37 | 12.1 | 416 | 19.2 |
|  | 4. Very | 34 | 9.8 | 109 | 16.6 | 29 | 17.1 | 19 | 10.2 | 20 | 25.3 | 45 | 17.8 | 19 | 19.6 | 8 | 12.9 | 43 | 14.1 | 327 | 15.1 |
|  | 5. Ex. rel. | 42 | 12.1 | 147 | 22.3 | 24 | 14.1 | 20 | 10.7 | 14 | 17.7 | 52 | 20.6 | 19 | 19.6 | 10 | 16.1 | 54 | 17.7 | 382 | 17.6 |
| Concern that physical health care received by service users I work with may not be adequate | 1. Not rel. | 108 | 31.4 | 109 | 16.5 | 21 | 12.4 | 73 | 39.0 | 22 | 27.5 | 25 | 10.0 | 9 | 9.4 | 26 | 41.9 | 107 | 35.2 | 505 | 23.4 |
|  | 2. Slightly | 83 | 24.1 | 140 | 21.2 | 59 | 34.7 | 36 | 19.3 | 13 | 16.3 | 47 | 18.7 | 19 | 19.8 | 8 | 12.9 | 56 | 18.4 | 461 | 21.3 |
|  | 3. Mod. | 76 | 22.1 | 153 | 23.2 | 37 | 21.8 | 35 | 18.7 | 22 | 27.5 | 69 | 27.5 | 24 | 25.0 | 14 | 22.6 | 60 | 19.7 | 492 | 22.8 |
|  | 4. Very | 42 | 12.2 | 147 | 22.3 | 39 | 22.9 | 25 | 13.4 | 9 | 11.3 | 57 | 22.7 | 31 | 32.3 | 9 | 14.5 | 47 | 15.5 | 407 | 18.8 |
|  | 5. Ex. rel. | 35 | 10.2 | 111 | 16.8 | 14 | 8.2 | 18 | 9.6 | 14 | 17.5 | 53 | 21.1 | 13 | 13.5 | 5 | 8.1 | 34 | 11.2 | 297 | 13.7 |
| Difficulty putting infection control measures into practice in the setting I work in | 1. Not rel. | 128 | 37.0 | 140 | 21.2 | 26 | 15.3 | 108 | 57.1 | 27 | 33.8 | 44 | 17.5 | 15 | 15.5 | 24 | 38.1 | 125 | 40.9 | 642 | 29.5 |
|  | 2. Slightly | 78 | 22.5 | 142 | 21.5 | 47 | 27.7 | 24 | 12.7 | 13 | 16.3 | 67 | 26.6 | 21 | 21.7 | 14 | 22.2 | 56 | 18.3 | 463 | 21.3 |
|  | 3. Mod. | 57 | 16.5 | 136 | 20.5 | 37 | 21.8 | 26 | 13.8 | 10 | 12.5 | 58 | 23.0 | 24 | 24.7 | 11 | 17.5 | 43 | 14.1 | 404 | 18.6 |
|  | 4. Very | 45 | 13.0 | 118 | 17.8 | 30 | 17.7 | 14 | 7.4 | 16 | 20.0 | 39 | 15.5 | 23 | 23.7 | 10 | 15.9 | 36 | 11.8 | 331 | 15.2 |
|  | 5. Ex. rel. | 38 | 11.0 | 126 | 19.0 | 30 | 17.7 | 17 | 9.0 | 14 | 17.5 | 44 | 17.5 | 14 | 14.4 | 4 | 6.4 | 46 | 15.0 | 333 | 15.3 |

| **Current work challenges for all participants by profession, in order of % rated ‘Very’ and ‘Extremely relevant’ combined for ‘All professions’ (n=2,180)** | | **Psych-**  **ologist (n=347)** | | **Nurse**  **(n=664)** | | **Occup-**  **ational**  **therapist**  **(n=171)** | | **Other**  **qualified**  **therapist**  **(n=189)** | | **Peer**  **support**  **worker**  **(n=80)** | | **Psych-**  **iatrist**  **(n=254)** | | **Social**  **worker**  **(n=97)** | | **Manager,**  **no prof.**  **qual.**  **(n=63)** | | **Other**  **worker**  **(n=307)** | | **All prof-**  **essions**  **(n=2,180)** | |
| --- | --- | --- | --- | --- | --- | --- | --- | --- | --- | --- | --- | --- | --- | --- | --- | --- | --- | --- | --- | --- | --- |
|  |  | **n** | **%*** | **n** | **%** | **n** | **%** | **n** | **%** | **n** | **%** | **n** | **%** | **n** | **%** | **n** | **%** | **n** | **%** | **n** | **%** |
| Greater workload than usual | 1. Not rel. | 93 | 26.8 | 144 | 21.7 | 58 | 33.9 | 64 | 33.9 | 17 | 21.3 | 72 | 28.4 | 27 | 27.8 | 11 | 17.5 | 97 | 31.6 | 587 | 26.9 |
|  | 2. Slightly | 51 | 14.7 | 120 | 18.1 | 29 | 17.0 | 40 | 21.2 | 17 | 21.3 | 52 | 20.5 | 14 | 14.4 | 1 | 1.6 | 57 | 18.6 | 381 | 17.5 |
|  | 3. Mod. | 97 | 28.0 | 175 | 26.4 | 49 | 28.7 | 50 | 26.5 | 21 | 26.3 | 69 | 27.2 | 25 | 25.8 | 21 | 33.3 | 82 | 26.7 | 590 | 27.1 |
|  | 4. Very | 57 | 16.4 | 137 | 20.6 | 22 | 12.9 | 20 | 10.6 | 17 | 21.3 | 34 | 13.4 | 21 | 21.7 | 15 | 23.8 | 51 | 16.6 | 375 | 17.2 |
|  | 5. Ex. rel. | 49 | 14.1 | 88 | 13.3 | 13 | 7.6 | 15 | 7.9 | 8 | 10.0 | 27 | 10.6 | 10 | 10.3 | 15 | 23.8 | 20 | 6.5 | 247 | 11.3 |
| Increased difficulty managing work-life balance, for example because of loss of childcare | 1. Not rel. | 108 | 31.2 | 307 | 46.2 | 75 | 43.9 | 67 | 35.5 | 39 | 49.4 | 112 | 44.4 | 42 | 43.8 | 30 | 47.6 | 153 | 50.0 | 940 | 43.2 |
|  | 2. Slightly | 71 | 20.5 | 108 | 16.3 | 33 | 19.3 | 36 | 19.1 | 13 | 16.5 | 44 | 17.5 | 16 | 16.7 | 8 | 12.7 | 52 | 17.0 | 381 | 17.5 |
|  | 3. Mod. | 54 | 15.6 | 87 | 13.1 | 19 | 11.1 | 33 | 17.5 | 7 | 8.9 | 41 | 16.3 | 18 | 18.8 | 4 | 6.4 | 38 | 12.4 | 301 | 13.9 |
|  | 4. Very | 55 | 15.9 | 75 | 11.3 | 27 | 15.8 | 27 | 14.3 | 8 | 10.1 | 23 | 9.1 | 11 | 11.5 | 9 | 14.3 | 25 | 8.2 | 261 | 12.0 |
|  | 5. Ex. rel. | 58 | 16.8 | 87 | 13.1 | 17 | 9.9 | 26 | 13.8 | 12 | 15.2 | 32 | 12.7 | 9 | 9.4 | 12 | 19.1 | 38 | 12.4 | 291 | 13.4 |
| Lack of protective clothing (PPE) and equipment needed for infection control | 1. Not rel. | 158 | 45.7 | 174 | 26.2 | 44 | 26.0 | 117 | 62.2 | 24 | 30.0 | 63 | 24.8 | 14 | 14.6 | 25 | 41.0 | 133 | 43.6 | 758 | 34.9 |
|  | 2. Slightly | 74 | 21.4 | 173 | 26.1 | 44 | 26.0 | 28 | 14.9 | 18 | 22.5 | 63 | 24.8 | 19 | 19.8 | 10 | 16.4 | 47 | 15.4 | 477 | 22.0 |
|  | 3. Mod. | 41 | 11.9 | 145 | 21.9 | 41 | 24.3 | 22 | 11.7 | 11 | 13.8 | 54 | 21.3 | 28 | 29.2 | 11 | 18.0 | 42 | 13.8 | 396 | 18.3 |
|  | 4. Very | 36 | 10.4 | 76 | 11.5 | 24 | 14.2 | 10 | 5.3 | 13 | 16.3 | 30 | 11.8 | 21 | 21.9 | 11 | 18.0 | 44 | 14.4 | 265 | 12.2 |
|  | 5. Ex. rel. | 37 | 10.7 | 95 | 14.3 | 16 | 9.5 | 11 | 5.9 | 14 | 17.5 | 44 | 17.3 | 14 | 14.6 | 4 | 6.6 | 39 | 12.8 | 274 | 12.6 |
| Safeguarding and other risk management processes cannot be adequately mobilised due to limited social care, legal or police response | 1. Not rel. | 115 | 33.3 | 152 | 23.1 | 37 | 21.8 | 62 | 32.8 | 29 | 36.3 | 48 | 19.0 | 9 | 9.4 | 26 | 41.3 | 125 | 40.9 | 609 | 28.1 |
|  | 2. Slightly | 106 | 30.7 | 152 | 23.1 | 49 | 28.8 | 59 | 31.2 | 17 | 21.3 | 56 | 22.1 | 23 | 24.0 | 15 | 23.8 | 72 | 23.5 | 550 | 25.4 |
|  | 3. Mod. | 63 | 18.3 | 158 | 24.0 | 46 | 27.1 | 36 | 19.1 | 13 | 16.3 | 68 | 26.9 | 25 | 26.0 | 11 | 17.5 | 50 | 16.3 | 471 | 21.7 |
|  | 4. Very | 40 | 11.6 | 112 | 17.0 | 26 | 15.3 | 20 | 10.6 | 18 | 22.5 | 51 | 20.2 | 25 | 26.0 | 4 | 6.4 | 37 | 12.1 | 333 | 15.4 |
|  | 5. Ex. rel. | 21 | 6.1 | 84 | 12.8 | 12 | 7.1 | 12 | 6.4 | 3 | 3.8 | 30 | 11.9 | 14 | 14.6 | 7 | 11.1 | 22 | 7.2 | 205 | 9.5 |
| Staff shortages (more than is usual in this setting) | 1. Not rel. | 134 | 38.6 | 174 | 26.4 | 42 | 24.6 | 104 | 55.0 | 29 | 36.7 | 42 | 16.7 | 20 | 20.6 | 17 | 27.9 | 123 | 40.3 | 690 | 31.8 |
|  | 2. Slightly | 99 | 28.5 | 171 | 26.0 | 57 | 33.3 | 44 | 23.3 | 17 | 21.5 | 75 | 29.8 | 28 | 28.9 | 12 | 19.7 | 67 | 22.0 | 572 | 26.4 |
|  | 3. Mod. | 64 | 18.4 | 134 | 20.4 | 35 | 20.5 | 22 | 11.6 | 12 | 15.2 | 68 | 27.0 | 24 | 24.7 | 9 | 14.8 | 51 | 16.7 | 419 | 19.3 |
|  | 4. Very | 31 | 8.9 | 91 | 13.8 | 19 | 11.1 | 10 | 5.3 | 11 | 13.9 | 34 | 13.5 | 15 | 15.5 | 11 | 18.0 | 35 | 11.5 | 258 | 11.9 |
|  | 5. Ex. rel. | 19 | 5.5 | 88 | 13.4 | 18 | 10.5 | 9 | 4.8 | 10 | 12.7 | 33 | 13.1 | 10 | 10.3 | 12 | 19.7 | 29 | 9.5 | 228 | 10.5 |
| Working longer hours than usual | 1. Not rel. | 124 | 35.7 | 213 | 32.1 | 84 | 49.1 | 90 | 47.6 | 36 | 45.0 | 86 | 33.9 | 36 | 37.1 | 10 | 15.9 | 159 | 52.0 | 842 | 38.7 |
|  | 2. Slightly | 62 | 17.9 | 123 | 18.6 | 32 | 18.7 | 30 | 15.9 | 11 | 13.8 | 52 | 20.5 | 22 | 22.7 | 4 | 6.4 | 53 | 17.3 | 390 | 17.9 |
|  | 3. Mod. | 85 | 24.5 | 155 | 23.4 | 28 | 16.4 | 37 | 19.6 | 17 | 21.3 | 61 | 24.0 | 23 | 23.7 | 28 | 44.4 | 52 | 17.0 | 487 | 22.4 |
|  | 4. Very | 39 | 11.2 | 101 | 15.2 | 15 | 8.8 | 20 | 10.6 | 8 | 10.0 | 30 | 11.8 | 6 | 6.2 | 6 | 9.5 | 30 | 9.8 | 255 | 11.7 |
|  | 5. Ex. rel. | 37 | 10.7 | 71 | 10.7 | 12 | 7.0 | 12 | 6.4 | 8 | 10.0 | 25 | 9.8 | 10 | 10.3 | 15 | 23.8 | 12 | 3.9 | 204 | 9.4 |

| **Current work challenges for all participants by profession, in order of % rated ‘Very’ and ‘Extremely relevant’ combined for ‘All professions’ (n=2,180)** | | **Psych-**  **ologist (n=347)** | | **Nurse**  **(n=664)** | | **Occup-**  **ational**  **therapist**  **(n=171)** | | **Other**  **qualified**  **therapist**  **(n=189)** | | **Peer**  **support**  **worker**  **(n=80)** | | **Psych-**  **iatrist**  **(n=254)** | | **Social**  **worker**  **(n=97)** | | **Manager,**  **no prof.**  **qual.**  **(n=63)** | | **Other**  **worker**  **(n=307)** | | **All prof-**  **essions**  **(n=2,180)** | |
| --- | --- | --- | --- | --- | --- | --- | --- | --- | --- | --- | --- | --- | --- | --- | --- | --- | --- | --- | --- | --- | --- |
|  |  | **n** | **%*** | **n** | **%** | **n** | **%** | **n** | **%** | **n** | **%** | **n** | **%** | **n** | **%** | **n** | **%** | **n** | **%** | **n** | **%** |
| Working in a different setting or with different clinical problems from usual | 1. Not rel. | 195 | 56.5 | 298 | 45.1 | 78 | 46.4 | 69 | 36.7 | 28 | 35.0 | 123 | 48.6 | 42 | 43.3 | 24 | 38.7 | 146 | 47.7 | 1,008 | 46.5 |
|  | 2. Slightly | 69 | 20.0 | 93 | 14.1 | 28 | 16.7 | 39 | 20.7 | 20 | 25.0 | 56 | 22.1 | 10 | 10.3 | 14 | 22.6 | 50 | 16.3 | 381 | 17.6 |
|  | 3. Mod. | 41 | 11.9 | 105 | 15.9 | 33 | 19.6 | 21 | 11.2 | 13 | 16.3 | 38 | 15.0 | 18 | 18.6 | 11 | 17.7 | 44 | 14.4 | 324 | 14.9 |
|  | 4. Very | 19 | 5.5 | 91 | 13.8 | 22 | 13.1 | 33 | 17.6 | 12 | 15.0 | 23 | 9.1 | 14 | 14.4 | 8 | 12.9 | 34 | 11.1 | 257 | 11.9 |
|  | 5. Ex. rel. | 21 | 6.1 | 74 | 11.2 | 7 | 4.2 | 26 | 13.8 | 7 | 8.8 | 13 | 5.1 | 13 | 13.4 | 5 | 8.1 | 32 | 10.5 | 198 | 9.1 |
| Feeling less able to do my job than usual because my own well-being has suffered through the stresses of the pandemic | 1. Not rel. | 73 | 21.2 | 212 | 32.3 | 39 | 22.8 | 54 | 28.7 | 19 | 24.1 | 104 | 41.3 | 25 | 25.8 | 25 | 40.3 | 96 | 31.3 | 651 | 30.1 |
|  | 2. Slightly | 145 | 42.0 | 181 | 27.6 | 64 | 37.4 | 57 | 30.3 | 18 | 22.8 | 84 | 33.3 | 29 | 29.9 | 18 | 29.0 | 83 | 27.0 | 681 | 31.4 |
|  | 3. Mod. | 70 | 20.3 | 126 | 19.2 | 34 | 19.9 | 38 | 20.2 | 19 | 24.1 | 36 | 14.3 | 19 | 19.6 | 13 | 21.0 | 56 | 18.2 | 413 | 19.1 |
|  | 4. Very | 40 | 11.6 | 68 | 10.4 | 20 | 11.7 | 27 | 14.4 | 12 | 15.2 | 12 | 4.8 | 15 | 15.5 | 5 | 8.1 | 41 | 13.4 | 240 | 11.1 |
|  | 5. Ex. rel. | 17 | 4.9 | 70 | 10.7 | 14 | 8.2 | 12 | 6.4 | 11 | 13.9 | 16 | 6.4 | 9 | 9.3 | 1 | 1.6 | 31 | 10.1 | 181 | 8.4 |
| Feeling under pressure from managers or colleagues to be less cautious about infection control than I would like | 1. Not rel. | 207 | 60.0 | 361 | 54.5 | 89 | 52.1 | 145 | 76.7 | 46 | 57.5 | 153 | 60.2 | 43 | 44.8 | 41 | 65.1 | 190 | 62.3 | 1,282 | 59.0 |
|  | 2. Slightly | 55 | 15.9 | 107 | 16.2 | 31 | 18.1 | 17 | 9.0 | 15 | 18.8 | 36 | 14.2 | 15 | 15.6 | 9 | 14.3 | 36 | 11.8 | 322 | 14.8 |
|  | 3. Mod. | 34 | 9.9 | 70 | 10.6 | 25 | 14.6 | 6 | 3.2 | 6 | 7.5 | 23 | 9.1 | 15 | 15.6 | 8 | 12.7 | 29 | 9.5 | 216 | 9.9 |
|  | 4. Very | 19 | 5.5 | 59 | 8.9 | 13 | 7.6 | 10 | 5.3 | 6 | 7.5 | 20 | 7.9 | 13 | 13.5 | 5 | 7.9 | 18 | 5.9 | 163 | 7.5 |
|  | 5. Ex. rel. | 30 | 8.7 | 65 | 9.8 | 13 | 7.6 | 11 | 5.8 | 7 | 8.8 | 22 | 8.7 | 10 | 10.4 | 0 | 0.0 | 32 | 10.5 | 190 | 8.7 |
| Not enough of the team I'm working with are permanently employed in this setting (lots of bank/locum and redeployed staff) | 1. Not rel. | 251 | 72.5 | 353 | 53.5 | 108 | 63.2 | 151 | 80.8 | 47 | 58.8 | 145 | 57.8 | 63 | 65.0 | 38 | 62.3 | 214 | 69.7 | 1,376 | 63.5 |
|  | 2. Slightly | 61 | 17.6 | 120 | 18.2 | 30 | 17.5 | 15 | 8.0 | 12 | 15.0 | 52 | 20.7 | 15 | 15.5 | 7 | 11.5 | 38 | 12.4 | 351 | 16.2 |
|  | 3. Mod. | 17 | 4.9 | 75 | 11.4 | 17 | 9.9 | 13 | 7.0 | 10 | 12.5 | 28 | 11.2 | 8 | 8.3 | 8 | 13.1 | 14 | 4.6 | 191 | 8.8 |
|  | 4. Very | 11 | 3.2 | 57 | 8.6 | 12 | 7.0 | 4 | 2.1 | 3 | 3.8 | 14 | 5.6 | 7 | 7.2 | 6 | 9.8 | 15 | 4.9 | 129 | 6.0 |
|  | 5. Ex. rel. | 6 | 1.7 | 55 | 8.3 | 4 | 2.3 | 4 | 2.1 | 8 | 10.0 | 12 | 4.8 | 4 | 4.1 | 2 | 3.3 | 26 | 8.5 | 121 | 5.6 |
| Pressure to accept redeployment to a setting where I don't feel happy to work | 1. Not rel. | 251 | 72.5 | 403 | 61.1 | 107 | 63.3 | 130 | 69.2 | 46 | 57.5 | 189 | 74.7 | 74 | 77.9 | 50 | 79.4 | 214 | 69.9 | 1,472 | 67.9 |
|  | 2. Slightly | 53 | 15.3 | 100 | 15.2 | 26 | 15.4 | 27 | 14.4 | 10 | 12.5 | 35 | 13.8 | 10 | 10.5 | 8 | 12.7 | 31 | 10.1 | 300 | 13.8 |
|  | 3. Mod. | 23 | 6.7 | 63 | 9.6 | 17 | 10.1 | 14 | 7.5 | 11 | 13.8 | 17 | 6.7 | 2 | 2.1 | 3 | 4.8 | 25 | 8.2 | 175 | 8.1 |
|  | 4. Very | 12 | 3.5 | 35 | 5.3 | 7 | 4.1 | 10 | 5.3 | 6 | 7.5 | 5 | 2.0 | 5 | 5.3 | 1 | 1.6 | 16 | 5.2 | 97 | 4.5 |
|  | 5. Ex. rel. | 7 | 2.0 | 59 | 8.9 | 12 | 7.1 | 7 | 3.7 | 7 | 8.8 | 7 | 2.8 | 4 | 4.2 | 1 | 1.6 | 20 | 6.5 | 124 | 5.7 |

| **Current work challenges for all participants by profession, in order of % rated ‘Very’ and ‘Extremely relevant’ combined for ‘All professions’ (n=2,180)** | | **Psych-**  **ologist (n=347)** | | **Nurse**  **(n=664)** | | **Occup-**  **ational**  **therapist**  **(n=171)** | | **Other**  **qualified**  **therapist**  **(n=189)** | | **Peer**  **support**  **worker**  **(n=80)** | | **Psych-**  **iatrist**  **(n=254)** | | **Social**  **worker**  **(n=97)** | | **Manager,**  **no prof.**  **qual.**  **(n=63)** | | **Other**  **worker**  **(n=307)** | | **All prof-**  **essions**  **(n=2,180)** | |
| --- | --- | --- | --- | --- | --- | --- | --- | --- | --- | --- | --- | --- | --- | --- | --- | --- | --- | --- | --- | --- | --- |
|  |  | **n** | **%*** | **n** | **%** | **n** | **%** | **n** | **%** | **n** | **%** | **n** | **%** | **n** | **%** | **n** | **%** | **n** | **%** | **n** | **%** |
| Problems commuting safely to work and back | 1. Not rel. | 271 | 78.1 | 506 | 76.2 | 136 | 79.5 | 154 | 81.5 | 58 | 72.5 | 190 | 74.8 | 72 | 74.2 | 42 | 66.7 | 247 | 80.5 | 1,684 | 77.3 |
|  | 2. Slightly | 34 | 9.8 | 59 | 8.9 | 15 | 8.8 | 16 | 8.5 | 7 | 8.8 | 28 | 11.0 | 7 | 7.2 | 10 | 15.9 | 14 | 4.6 | 190 | 8.7 |
|  | 3. Mod. | 20 | 5.8 | 39 | 5.9 | 7 | 4.1 | 8 | 4.2 | 4 | 5.0 | 17 | 6.7 | 9 | 9.3 | 7 | 11.1 | 15 | 4.9 | 126 | 5.8 |
|  | 4. Very | 10 | 2.9 | 29 | 4.4 | 9 | 5.3 | 1 | 0.5 | 8 | 10.0 | 11 | 4.3 | 4 | 4.1 | 3 | 4.8 | 14 | 4.6 | 89 | 4.1 |
|  | 5. Ex. rel. | 12 | 3.5 | 31 | 4.7 | 4 | 2.3 | 10 | 5.3 | 3 | 3.8 | 8 | 3.2 | 5 | 5.2 | 1 | 1.6 | 17 | 5.5 | 91 | 4.2 |
| *Percentages are of those who answered. The highest number of missing responses for a challenge was 18 (0.8% of total participants, n=2,180). This was for challenges: 'Pressures resulting from the need to support colleagues through the stresses associated with the pandemic' and 'Concern that physical health care received by service users I work with may not be adequate', where for each variables n=2,162. | | | | | | | | | | | | | | | | | | | | | |
| ‘Not rel.’=’Not relevant’, ‘Mod.’=Moderately, ‘Ex. rel.’=’Extremely relevant’. | | | | | | | | | | | | | | | | | | | | | |

# Table 30: Current work challenges for all participants by managerial role, in order of % rated ‘Very’ and ‘Extremely relevant’ combined for ‘All roles’ (n=2,180)

|  | | **Manager/ lead clinician (n=826)** | | | **Non-manager/ lead (n=1,350)** | | | **All roles (n=2,180)** | | |  |
| --- | --- | --- | --- | --- | --- | --- | --- | --- | --- | --- | --- |
|  |  |  |  |  |  |  |  |  |  |  |  |
|  |  | **n** | **%*** | | **n** | | **%** | **n** | | **%** |  |
| Having to adapt too quickly to new ways of working | 1. Not rel. | 39 | | 4.8 | 73 | 5.4 | | 112 | 5.2 | |  |
|  | 2. Slightly | 138 | | 16.8 | 250 | 18.6 | | 389 | 18.0 | |  |
|  | 3. Mod. | 171 | | 20.9 | 280 | 20.9 | | 452 | 20.9 | |  |
|  | 4. Very | 193 | | 23.5 | 345 | 25.7 | | 539 | 24.9 | |  |
|  | 5. Ex. rel. | 279 | | 34.0 | 393 | 29.3 | | 673 | 31.1 | |  |
| The risk I or my colleagues could be infected with COVID-19 at work | 1. Not rel. | 82 | | 10.0 | 227 | 16.9 | | 309 | 14.3 | |  |
|  | 2. Slightly | 188 | | 22.9 | 258 | 19.2 | | 446 | 20.6 | |  |
|  | 3. Mod. | 220 | | 26.8 | 319 | 23.8 | | 540 | 24.9 | |  |
|  | 4. Very | 159 | | 19.4 | 254 | 18.9 | | 416 | 19.2 | |  |
|  | 5. Ex. rel. | 172 | | 21.0 | 284 | 21.2 | | 456 | 21.0 | |  |
| Having to respond to additional mental health needs that appear to result from COVID-19 | 1. Not rel. | 78 | | 9.5 | 134 | 10.0 | | 213 | 9.8 | |  |
|  | 2. Slightly | 205 | | 24.9 | 311 | 23.1 | | 519 | 23.9 | |  |
|  | 3. Mod. | 233 | | 28.3 | 345 | 25.7 | | 578 | 26.6 | |  |
|  | 4. Very | 159 | | 19.3 | 286 | 21.3 | | 445 | 20.5 | |  |
|  | 5. Ex. rel. | 148 | | 18.0 | 268 | 19.9 | | 416 | 19.2 | |  |
| Pressures resulting from the need to support colleagues through the stresses associated with the pandemic | 1. Not rel. | 30 | | 3.7 | 185 | 13.8 | | 215 | 9.9 | |  |
|  | 2. Slightly | 163 | | 19.9 | 387 | 28.9 | | 551 | 25.5 | |  |
|  | 3. Mod. | 204 | | 24.9 | 341 | 25.5 | | 546 | 25.3 | |  |
|  | 4. Very | 221 | | 27.0 | 239 | 17.8 | | 462 | 21.4 | |  |
|  | 5. Ex. rel. | 200 | | 24.5 | 188 | 14.0 | | 388 | 18.0 | |  |
| Service users no longer getting an acceptable service due to service reconfiguration because of COVID-19 | 1. Not rel. | 115 | | 14.0 | 198 | 14.8 | | 313 | 14.5 | |  |
|  | 2. Slightly | 188 | | 22.9 | 307 | 22.9 | | 496 | 22.9 | |  |
|  | 3. Mod. | 209 | | 25.4 | 329 | 24.6 | | 540 | 24.9 | |  |
|  | 4. Very | 168 | | 20.4 | 278 | 20.8 | | 446 | 20.6 | |  |
|  | 5. Ex. rel. | 142 | | 17.3 | 228 | 17.0 | | 371 | 17.1 | |  |
| Having to learn to use new technologies too quickly and/or without sufficient training and support | 1. Not rel. | 126 | | 15.4 | 229 | 17.0 | | 355 | 16.4 | |  |
|  | 2. Slightly | 180 | | 22.0 | 336 | 25.0 | | 517 | 23.9 | |  |
|  | 3. Mod. | 193 | | 23.6 | 290 | 21.6 | | 485 | 22.4 | |  |
|  | 4. Very | 165 | | 20.2 | 255 | 19.0 | | 420 | 19.4 | |  |
|  | 5. Ex. rel. | 153 | | 18.7 | 235 | 17.5 | | 389 | 18.0 | |  |
| The risk family and friends may be infected with COVID-19 through me | 1. Not rel. | 127 | | 15.4 | 309 | 23.0 | | 436 | 20.1 | |  |
|  | 2. Slightly | 212 | | 25.8 | 291 | 21.7 | | 503 | 23.2 | |  |
|  | 3. Mod. | 195 | | 23.7 | 262 | 19.5 | | 458 | 21.1 | |  |
|  | 4. Very | 133 | | 16.2 | 213 | 15.9 | | 348 | 16.1 | |  |
|  | 5. Ex. rel. | 156 | | 19.0 | 266 | 19.8 | | 423 | 19.5 | |  |
| The risk that COVID-19 will spread between service users I'm working with | 1. Not rel. | 184 | | 22.4 | 435 | 32.3 | | 619 | 28.5 | |  |
|  | 2. Slightly | 178 | | 21.7 | 225 | 16.7 | | 403 | 18.6 | |  |
|  | 3. Mod. | 147 | | 17.9 | 229 | 17.0 | | 378 | 17.4 | |  |
|  | 4. Very | 147 | | 17.9 | 193 | 14.4 | | 342 | 15.8 | |  |
|  | 5. Ex. rel. | 164 | | 20.0 | 263 | 19.6 | | 427 | 19.7 | |  |
| Being expected to use new technologies without reliable access to necessary tools and equipment | 1. Not rel. | 181 | | 22.1 | 343 | 25.6 | | 524 | 24.2 | |  |
|  | 2. Slightly | 200 | | 24.4 | 309 | 23.0 | | 510 | 23.6 | |  |
|  | 3. Mod. | 157 | | 19.2 | 257 | 19.2 | | 415 | 19.2 | |  |
|  | 4. Very | 155 | | 18.9 | 204 | 15.2 | | 359 | 16.6 | |  |
|  | 5. Ex. rel. | 127 | | 15.5 | 228 | 17.0 | | 357 | 16.5 | |  |

| **Current work challenges for all participants by managerial role, in order of % rated ‘Very’ and ‘Extremely relevant’ combined for ‘All roles’ (n=2,180)** | | **Manager/ lead clinician (n=826)** | | **Non-manager/ lead (n=1,350)** | | **All roles (n=2,180)** | |  |
| --- | --- | --- | --- | --- | --- | --- | --- | --- |
|  |  | **n** | **%*** | **n** | **%** | **n** | **%** |  |
| Problems resulting from lack of access to testing | 1. Not rel. | 185 | 22.5 | 429 | 32.0 | 614 | 28.4 |  |
|  | 2. Slightly | 169 | 20.6 | 258 | 19.2 | 427 | 19.7 |  |
|  | 3. Mod. | 164 | 20.0 | 252 | 18.8 | 416 | 19.2 |  |
|  | 4. Very | 141 | 17.2 | 185 | 13.8 | 327 | 15.1 |  |
|  | 5. Ex. rel. | 162 | 19.7 | 217 | 16.2 | 382 | 17.6 |  |
| Concern that physical health care received by service users I work with may not be adequate | 1. Not rel. | 176 | 21.6 | 328 | 24.4 | 505 | 23.4 |  |
|  | 2. Slightly | 180 | 22.1 | 281 | 20.9 | 461 | 21.3 |  |
|  | 3. Mod. | 191 | 23.4 | 299 | 22.3 | 492 | 22.8 |  |
|  | 4. Very | 159 | 19.5 | 248 | 18.5 | 407 | 18.8 |  |
|  | 5. Ex. rel. | 110 | 13.5 | 186 | 13.9 | 297 | 13.7 |  |
| Difficulty putting infection control measures into practice in the setting I work in | 1. Not rel. | 216 | 26.2 | 426 | 31.7 | 642 | 29.5 |  |
|  | 2. Slightly | 195 | 23.7 | 268 | 19.9 | 463 | 21.3 |  |
|  | 3. Mod. | 165 | 20.0 | 239 | 17.8 | 404 | 18.6 |  |
|  | 4. Very | 120 | 14.6 | 209 | 15.5 | 331 | 15.2 |  |
|  | 5. Ex. rel. | 128 | 15.5 | 203 | 15.1 | 333 | 15.3 |  |
| Greater workload than usual | 1. Not rel. | 146 | 17.7 | 439 | 32.5 | 587 | 26.9 |  |
|  | 2. Slightly | 109 | 13.2 | 272 | 20.2 | 381 | 17.5 |  |
|  | 3. Mod. | 236 | 28.6 | 352 | 26.1 | 590 | 27.1 |  |
|  | 4. Very | 185 | 22.4 | 190 | 14.1 | 375 | 17.2 |  |
|  | 5. Ex. rel. | 150 | 18.2 | 97 | 7.2 | 247 | 11.3 |  |
| Increased difficulty managing work-life balance, for example because of loss of childcare | 1. Not rel. | 342 | 41.5 | 597 | 44.4 | 940 | 43.2 |  |
|  | 2. Slightly | 147 | 17.8 | 234 | 17.4 | 381 | 17.5 |  |
|  | 3. Mod. | 117 | 14.2 | 184 | 13.7 | 301 | 13.9 |  |
|  | 4. Very | 96 | 11.7 | 164 | 12.2 | 261 | 12.0 |  |
|  | 5. Ex. rel. | 122 | 14.8 | 167 | 12.4 | 291 | 13.4 |  |
| Lack of protective clothing (PPE) and equipment needed for infection control | 1. Not rel. | 269 | 32.7 | 489 | 36.4 | 758 | 34.9 |  |
|  | 2. Slightly | 182 | 22.1 | 294 | 21.9 | 477 | 22.0 |  |
|  | 3. Mod. | 151 | 18.4 | 245 | 18.2 | 396 | 18.3 |  |
|  | 4. Very | 110 | 13.4 | 153 | 11.4 | 265 | 12.2 |  |
|  | 5. Ex. rel. | 110 | 13.4 | 163 | 12.1 | 274 | 12.6 |  |
| Safeguarding and other risk management processes cannot be adequately mobilised due to limited social care, legal or police response | 1. Not rel. | 206 | 25.2 | 402 | 29.9 | 609 | 28.1 |  |
|  | 2. Slightly | 211 | 25.8 | 337 | 25.1 | 550 | 25.4 |  |
|  | 3. Mod. | 184 | 22.5 | 287 | 21.3 | 471 | 21.7 |  |
|  | 4. Very | 126 | 15.4 | 207 | 15.4 | 333 | 15.4 |  |
|  | 5. Ex. rel. | 92 | 11.2 | 112 | 8.3 | 205 | 9.5 |  |
| Staff shortages (more than is usual in this setting) | 1. Not rel. | 216 | 26.3 | 474 | 35.3 | 690 | 31.8 |  |
|  | 2. Slightly | 230 | 28.0 | 341 | 25.4 | 572 | 26.4 |  |
|  | 3. Mod. | 172 | 21.0 | 245 | 18.3 | 419 | 19.3 |  |
|  | 4. Very | 104 | 12.7 | 153 | 11.4 | 258 | 11.9 |  |
|  | 5. Ex. rel. | 99 | 12.1 | 129 | 9.6 | 228 | 10.5 |  |
| Working longer hours than usual | 1. Not rel. | 201 | 24.3 | 639 | 47.4 | 842 | 38.7 |  |
|  | 2. Slightly | 130 | 15.7 | 259 | 19.2 | 390 | 17.9 |  |
|  | 3. Mod. | 228 | 27.6 | 259 | 19.2 | 487 | 22.4 |  |
|  | 4. Very | 135 | 16.3 | 120 | 8.9 | 255 | 11.7 |  |
|  | 5. Ex. rel. | 132 | 16.0 | 71 | 5.3 | 204 | 9.4 |  |
| Working in a different setting or with different clinical problems from usual | 1. Not rel. | 414 | 50.3 | 592 | 44.2 | 1,008 | 46.5 |  |
|  | 2. Slightly | 135 | 16.4 | 245 | 18.3 | 381 | 17.6 |  |
|  | 3. Mod. | 128 | 15.6 | 195 | 14.5 | 324 | 14.9 |  |
|  | 4. Very | 82 | 10.0 | 175 | 13.1 | 257 | 11.9 |  |
|  | 5. Ex. rel. | 64 | 7.8 | 134 | 10.0 | 198 | 9.1 |  |

| **Current work challenges by managerial role, in order of % ‘Very’ and ‘Extremely relevant’ combined for ‘All roles’ (n=2,180)** | | **Manager/ lead clinician (n=826)** | | **Non-manager/ lead (n=1,350)** | | **All roles (n=2,180)** | |  |
| --- | --- | --- | --- | --- | --- | --- | --- | --- |
|  |  | **n** | **%*** | **n** | **%** | **n** | **%** |  |
| Feeling less able to do my job than usual because my own well-being has suffered through the stresses of the pandemic | 1. Not rel. | 287 | 35.0 | 363 | 27.0 | 651 | 30.1 |  |
|  | 2. Slightly | 278 | 33.9 | 402 | 29.9 | 681 | 31.4 |  |
|  | 3. Mod. | 142 | 17.3 | 271 | 20.2 | 413 | 19.1 |  |
|  | 4. Very | 68 | 8.3 | 170 | 12.7 | 240 | 11.1 |  |
|  | 5. Ex. rel. | 44 | 5.4 | 137 | 10.2 | 181 | 8.4 |  |
| Feeling under pressure from managers or colleagues to be less cautious about infection control than I would like | 1. Not rel. | 482 | 58.6 | 799 | 59.3 | 1,282 | 59.0 |  |
|  | 2. Slightly | 121 | 14.7 | 199 | 14.8 | 322 | 14.8 |  |
|  | 3. Mod. | 89 | 10.8 | 127 | 9.4 | 216 | 9.9 |  |
|  | 4. Very | 61 | 7.4 | 102 | 7.6 | 163 | 7.5 |  |
|  | 5. Ex. rel. | 69 | 8.4 | 120 | 8.9 | 190 | 8.7 |  |
| Not enough of the team I'm working with are permanently employed in this setting (lots of bank/locum and redeployed staff) | 1. Not rel. | 489 | 59.6 | 885 | 65.9 | 1,376 | 63.5 |  |
|  | 2. Slightly | 153 | 18.6 | 196 | 14.6 | 351 | 16.2 |  |
|  | 3. Mod. | 78 | 9.5 | 113 | 8.4 | 191 | 8.8 |  |
|  | 4. Very | 55 | 6.7 | 74 | 5.5 | 129 | 6.0 |  |
|  | 5. Ex. rel. | 46 | 5.6 | 75 | 5.6 | 121 | 5.6 |  |
| Pressure to accept redeployment to a setting where I don't feel happy to work | 1. Not rel. | 589 | 71.7 | 881 | 65.6 | 1,472 | 67.9 |  |
|  | 2. Slightly | 113 | 13.8 | 186 | 13.9 | 300 | 13.8 |  |
|  | 3. Mod. | 58 | 7.1 | 117 | 8.7 | 175 | 8.1 |  |
|  | 4. Very | 27 | 3.3 | 70 | 5.2 | 97 | 4.5 |  |
|  | 5. Ex. rel. | 34 | 4.1 | 89 | 6.6 | 124 | 5.7 |  |
| Problems commuting safely to work and back | 1. Not rel. | 635 | 76.9 | 1,047 | 77.6 | 1,684 | 77.3 |  |
|  | 2. Slightly | 81 | 9.8 | 109 | 8.1 | 190 | 8.7 |  |
|  | 3. Mod. | 50 | 6.1 | 76 | 5.6 | 126 | 5.8 |  |
|  | 4. Very | 32 | 3.9 | 56 | 4.2 | 89 | 4.1 |  |
|  | 5. Ex. rel. | 28 | 3.4 | 62 | 4.6 | 91 | 4.2 |  |
| *Percentages are of those who answered. The highest number of missing responses for a challenge was 18 (0.8% of total participants, n=2,180). This was for challenges: 'Pressures resulting from the need to support colleagues through the stresses associated with the pandemic' and 'Concern that physical health care received by service users I work with may not be adequate', where for each variables n=2,162. | | | | | | | |  |
